# Mass Spectrometric and Glycan Microarray–Based Characterization of the Filarial Nematode *Brugia malayi* Glycome Reveals Anionic and Zwitterionic Glycan Antigens

**DOI:** 10.1016/j.mcpro.2022.100201

**Published:** 2022-01-20

**Authors:** Laudine M.C. Petralia, Angela van Diepen, Lena A. Lokker, D. Linh Nguyen, Erliyani Sartono, Vishal Khatri, Ramaswamy Kalyanasundaram, Christopher H. Taron, Jeremy M. Foster, Cornelis H. Hokke

**Affiliations:** 1 Department of Parasitology, Leiden University – Center of Infectious Diseases, Leiden University Medical Center, Leiden, The Netherlands; 2 Division of Protein Expression & Modification, New England Biolabs, Ipswich, Massachusetts, USA; 3 Department of Biomedical Sciences, University of Illinois College of Medicine at Rockford, Rockford, Illinois, USA

**Keywords:** lymphatic filariasis, *Brugia malayi*, *N*-glycan, GSL glycan, glycan sequencing, glycan microarray, antiglycan response, antigenic glycans, antibodies, 2-AA, 2-aminobenzoic acid, ACN, acetonitrile, C18, octadecylsilane, DEC, diethylcarbamazine citrate, Gal, galactose, GLAD, GLycan Array Dashboard, GlcA, glucuronic acid, GSL, glycosphingolipid, HexA, hexuronic acid, HexNAc, *N*-acetylhexosamine, HF, hydrofluoric acid, Ig, immunoglobulin, L3, third-stage larvae, LDN, LacdiNAc, LF, lymphatic filariasis, mAb, monoclonal antibody, Man_x–y_, oligomannosidic structures with x to y mannose residues, MeOH, methanol, MF, microfilariae, MFI, median fluorescence intensity, MQ, milliQ water, MS, mass spectrometry, MS/MS, tandem MS, N_2_, nitrogen, NCFG, National Center for Functional Glycomics, NEB, New England Biolabs, PC, phosphorylcholine, PNGase F, peptide:N-glycosidase F, RP, reverse-phase, TFA, trifluoroacetic acid, UHPLC, ultra-HPLC, wpi, weeks postinfection

## Abstract

Millions of people worldwide are infected with filarial nematodes, responsible for lymphatic filariasis (LF) and other diseases causing chronic disablement. Elimination programs have resulted in a substantial reduction of the rate of infection in certain areas creating a need for improved diagnostic tools to establish robust population surveillance and avoid LF resurgence. Glycans from parasitic helminths are emerging as potential antigens for use in diagnostic assays. However, despite its crucial role in host–parasite interactions, filarial glycosylation is still largely, structurally, and functionally uncharacterized. Therefore, we investigated the glycan repertoire of the filarial nematode *Brugia malayi*. Glycosphingolipid and N-linked glycans were extracted from several life-stages using enzymatic release and characterized using a combination of MALDI-TOF-MS and glycan sequencing techniques. Next, glycans were purified by HPLC and printed onto microarrays to assess the host anti-glycan antibody response. Comprehensive glycomic analysis of *B. malayi* revealed the presence of several putative antigenic motifs such as phosphorylcholine and terminal glucuronic acid. Glycan microarray screening showed a recognition of most *B. malayi* glycans by immunoglobulins from rhesus macaques at different time points after infection, which permitted the characterization of the dynamics of anti-glycan immunoglobulin G and M during the establishment of brugian filariasis. A significant level of IgG binding to the parasite glycans was also detected in infected human plasma, while IgG binding to glycans decreased after anthelmintic treatment. Altogether, our work identifies *B. malayi* glycan antigens and reveals antibody responses from the host that could be exploited as potential markers for LF.

## Highlights

• Antigenic *B. malayi N*-linked and GSL glycans were structurally defined.
• IgG/IgM is induced to a subset of *B. malayi* glycans upon infection of rhesus macaques.
• Preferential IgG response to *B. malayi* glycans observed in chronically infected humans.
• Marked drop of anti-glycan IgG following treatment of individuals with anthelminthic.

## Graphical Abstract

**Figure:**
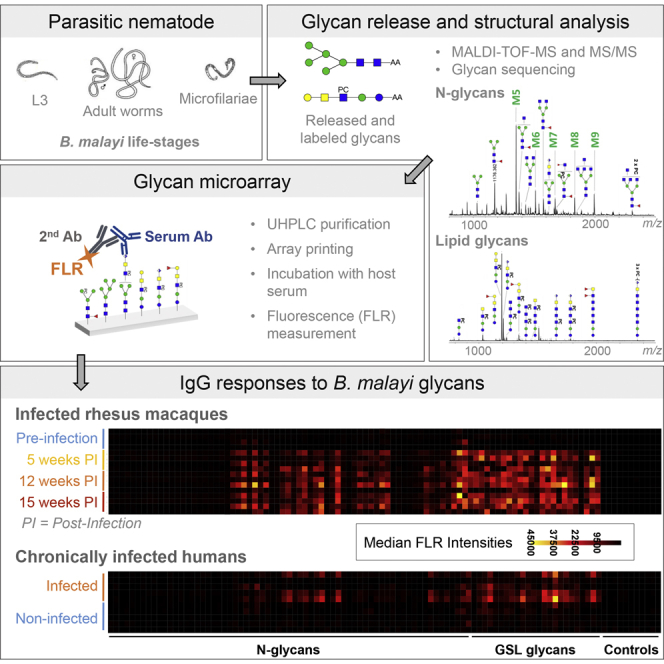

## In Brief

While parasite glycans form the basis of highly successful diagnostic assays, filarial glycosylation is largely unexplored. Therefore, we conducted a comprehensive structural characterization of *N*-linked and GSL glycans of *Brugia malayi*. Our work revealed anionic and zwitterionic glycan motifs as major antibody targets. Glycan microarray analysis showed the induction of IgG and IgM to these glycans in a rhesus macaque infection model as well as a specific IgG response associated with infection in individuals from a *Brugia malayi* endemic area.

Parasitic worms responsible for several neglected tropical diseases represent a global public health burden and cause disease in hundreds of millions of impoverished people (1, 2) (https://www.cdc.gov/parasites/resources/pdf/ntd_factsheet.pdf; https://www.cdc.gov/globalhealth/ntd/diseases/index.html; https://www.who.int/data/gho/data/themes/neglected-tropical-diseases). Three species of closely related filarial nematodes, *Wuchereria bancrofti* (*W. bancrofti*), *Brugia malayi* (*B. malayi*), and *Brugia timori*, cause lymphatic filariasis (LF). The disabling consequences of LF, including hydrocele, lymphedemas, and elephantiasis, form a daily life burden for at least 36 million people (https://www.who.int/news-room/fact-sheets/detail/lymphatic-filariasis). LF has been targeted for elimination since 2000 in the framework of the Global Program to Eliminate Lymphatic Filariasis (3). Substantial progress toward LF elimination has been made, but World Health Organization targets for 2020 have not been sufficiently met, and elimination of LF remains a target also in the new neglected tropical disease road map for 2021 to 2030 (1) (https://www.who.int/neglected_diseases/Ending-the-neglect-to-attain-the-SDGs--NTD-Roadmap.pdf?ua=1). The elimination program for LF relies on long-term community-wide distribution of a limited number of available drugs—namely ivermectin, albendazole, and diethylcarbamazine citrate (DEC) (https://www.who.int/publications/i/item/9789240696471) (4)—that is logistically difficult to achieve. In addition to preventive chemotherapy, diagnostic screens are also critical to the successful control, prevention, and treatment of LF. The standard method for diagnosing active infection is the microscopic identification of microfilariae (MF) in blood collected at night when these larval forms appear in the peripheral circulation (https://www.who.int/teams/control-of-neglected-tropical-diseases/lymphatic-filariasis/diagnosis-and-treatment; https://www.cdc.gov/parasites/lymphaticfilariasis/diagnosis.html). Recommended alternatives to cumbersome microscopy are serologic techniques including the Alere Filariasis test strip (Abbott) for the detection of *W. bancrofti* antigen in human blood and the Brugia Rapid test (Reszon Diagnostics International) that measures immunoglobulin G4 (IgG4) against *Brugia* spp. However, limitations of these tools have recently been highlighted. These include inaccuracy because of crossreactivity of the Alere Filariasis test strip in areas of coendemicity with *Loa loa* and generation of false positives in the Brugia Rapid test because of the presence of IgG4 antibodies up to 2 years after successful treatment of infection (5, 6). While these tools have been invaluable for endemicity mapping and mass drug administration monitoring, they may not be appropriate in terms of both sensitivity and specificity for future surveillance and informing decisions to stop community treatment (7). Thus, efforts are needed to find and validate new diagnostic targets and assays adequate for LF surveillance (https://www.who.int/teams/control-of-neglected-tropical-diseases/lymphatic-filariasis/diagnosis-and-treatment) to properly assess elimination and minimize risk of disease resurgence from human residual microfoci or animal reservoirs (https://www.who.int/neglected_diseases/Ending-the-neglect-to-attain-the-SDGs--NTD-Roadmap.pdf?ua=1). Potential, but so far unexplored, sources of new diagnostic targets for LF are specific glycan antigens expressed by each of the filarial nematode species. First, significant progress in this area has been made for other parasitic helminths, specifically for the schistosomes (8). Excretory glycans of schistosomes detectable in urine or blood form the basis of highly successful assays for diagnosis of active infections (9, 10, 11). In addition, assays for the detection of antibodies to specific schistosome glycan antigens are holding potential for novel diagnostic tools (12, 13, 14, 15). Second, based on studies of the model organism *Caenorhabditis elegans* (*C. elegans*), nematodes are known to express many of the enzymes for glycoconjugate biosynthesis found in higher animals (16). Filarial nematodes have the capacity to synthesize complex and antigenic glycans as protein modifications (17, 18, 19) as well as glycosphingolipid (GSL) (20). Despite conserved features in their biosynthetic pathways (21), nematodes show a significant diversity in their glycans (22). They can include species-specific elements (*e.g.*, the terminal tyvelose residue detected in *Trichinella spiralis N*-glycans (23)), whereas stage (24, 25) and sex (26) specificity have also been described. Third, several studies attest to the importance of filarial nematode carbohydrate components for the modulation of the host immune system (27, 28) often linked to the presence of phosphorylcholine (PC) substituents (29, 30). Excreted/secreted glycoproteins such as the well-studied *Acanthocheilonema viteae* ES-62, which modulates complement system activation and promotes Th2-type immune responses (31, 32), or membrane glycoproteins, such as the cuticular glutathione peroxidase (gp29) from *Brugia pahangi*, thought to protect parasite membranes from oxidative damage (33) appear to be particularly essential to the filarial nematode. Finally, the monoclonal antibodies (mAbs) used in the Alere Filariasis test strip to detect *W. bancrofti* circulating filarial antigen are now known to bind to a carbohydrate epitope (34, 35). However, despite these major roles of glycoconjugates in parasite biology, a broader understanding of filarial nematode glycomes is still lacking from both structural and functional perspectives.

Therefore, we investigated the glycan repertoire of the filarial nematode *B. malayi*. We used a mass spectrometry (MS)-based method in combination with enzymatic and chemical glycan sequencing techniques and tandem MS (MS/MS) to characterize the structures of Asn (N)-linked and GSL-derived glycans expressed by the parasite, in particular aiming to identify species-specific glycan elements that might constitute potential biomarkers for infection. To study glycan recognition by antiglycan antibodies from *B. malayi*-infected hosts, we generated glycan microarray–containing glycans isolated from the adult worms. We used a longitudinal serum collection from a cohort of *B. malayi*-infected rhesus macaques to characterize the appearance of antiglycan IgG and IgM during establishment of infection and a human plasma panel to study IgG response from chronically infected individuals both preanthelmintic and postanthelmintic treatments.

Our structural study gives a global overview of *B. malayi N*-linked and GSL glycans that not only corroborates previous literature but also exposes new features of *B. malayi* glycosylation. The glycan microarray evaluation highlights a strong recognition of a subset of *B. malayi* glycans by serum/plasma antibodies of the infected host. Thus, our work indicates that *B. malayi* glycans encompass antigenic motifs triggering a specific antibody response that could be exploited as potential diagnostic markers to detect LF.

## Experimental Procedures

### Structural Analysis of *B. malayi* Glycans

#### Enzymatic Release of *N*-glycans and GSL Glycans From Parasite Proteins and Lipids

*B. malayi* MF and adult worms (males and females) were purchased from TRS Labs Inc, from their life cycle maintained in gerbils and mosquitoes. Infective third-stage larvae (L3) were provided by the National Institutes of Health/National Institute of Allergy and Infectious Diseases Filariasis Research Reagent Resource Center (http://www.filariasiscenter.org/). Unless specified otherwise, reagents used for the experimental work were obtained from Sigma–Aldrich. A pellet of MF (∼0.5 ml), ∼1500 L3 larvae, 150 adult worms (mixed sex in a 1:1 ratio), and 50 adult males and 50 adult females were used as starting materials. In order to ensure that relative glycan abundance in each life stage was observed reproducibly, we performed two additional extractions on two separate batches of adult worms (n = ∼150, mixed sex in a 1:1 ratio) and on two separate batches of MF (∼0.5 ml pellet each time), on different occasions, generating three biological replicates for these two life stages. Samples were processed in parallel essentially following the protocol previously described in detail (36). Briefly, pools of parasite material were ground in a Potter-Elvehjem homogenizer in 1 ml of milliQ water (MQ) and then subjected to protein and lipid extraction by homogenization in methanol (MeOH) and chloroform in a final 4:7:13 ratio of MQ:MeOH:chloroform. Samples were sonicated and centrifuged at 4000 rpm for 5 min, and the upper phase was removed and replaced by 50% MeOH. This step was repeated twice, and (glyco-)lipids from the upper phase of extraction were subsequently purified using octadecylsilane (C18) cartridges (catalog no.: 7020-03; BAKERBOND spe, JT Baker). Cartridges were conditioned with MeOH and 10% MeOH before samples were applied to the columns and washed with MQ. Both flowthrough and wash were collected, combined, and used for an additional C18 purification on new C18 cartridges following the same procedure. Glycolipids were eluted from both cartridges with chloroform:MeOH:MQ (10:10:1), and elutions were combined and dried under nitrogen (N_2_) flow. Dried (glyco-)lipids were reconstituted in 500 μl 50 mM sodium acetate (pH 5) containing 0.1% natrium taurodeoxycholate, and lipid extracts were treated with 16 mU of recombinant endoglycoceramidase I (catalog no.: P0773; New England Biolabs [NEB]) for 48 h at 37 °C. After 24 h, another 16 mU of endoglycoceramidase I was added.

Simultaneously, (glyco-)proteins in the lower phase from the protein/lipid separation procedure described previously were pelleted using excess volumes of MeOH. Protein pellets were dried under N_2_ flow and then homogenized in PBS with 1.3% SDS and 0.1% β-mercaptoethanol. Denaturation was performed for 10 min at 95 °C, samples were cooled to room temperature, and Nondidet P-40 (1.3% final concentration) was added to each sample. *N*-linked glycans were released using 3500 units of peptide:N-glycosidase F (PNGase F) (catalog no.: P0709; NEB) and incubated at 37 °C for 24 h.

#### Cleanup of Released Glycans and Labeling With Anthranilic Acid (2-aminobenzoic Acid)

Following enzymatic release, *N*-linked and GSL glycans were purified sequentially on C18 and carbon cartridges (catalog no.: 57088; Sigma–Aldrich; Supelclean ENVI-Carb SPE). Released glycans were loaded on conditioned C18 cartridges as described previously. Flowthroughs from load and washes were combined for each sample and further purified using carbon solid phase extraction. Carbon cartridges were conditioned sequentially with acetonitrile (ACN), 50% ACN containing 0.1% trifluoroacetic acid (TFA), and MQ before applying the flowthroughs from C18 purifications. After washing with MQ, the glycans were eluted with subsequent addition of 25% ACN and then 50% ACN containing 0.1% TFA. Both elutions were pooled and dried using a rotational vacuum concentrator (Speedvac). The glycans were labeled with 2-aminobenzoic acid (2-AA) by reductive amination with sodium cyanoborohydride as described (37). Labeling was performed for 2 h at 65 °C. To remove labeling reagent excess, ACN was added to a final concentration of 75%, and the sample loaded onto Bio-Gel P10 Gel resin (catalog no.: 1504144; Bio-Rad) previously conditioned with 80% ACN. Glycans were eluted with MQ and dried using a Speedvac.

#### Elucidation of Glycan Structures

##### Hydrofluoric Acid Treatment

Hydrofluoric acid (HF) was used to selectively remove PC residues as well as α1–2/3/4-linked fucoses from the glycan backbone by mixing small aliquots of 2-AA-labeled glycans suspended in MQ with cold 48% HF in a 1:100 ratio. Samples were incubated at 4 °C for 48 h, and HF was then removed by evaporation under N_2_ flow. Multiple washes were performed by addition and subsequent N_2_ evaporation of MeOH. Finally, the HF-treated AA-labeled glycan samples were redissolved in MQ.

##### Exoglycosidase Digestions

A panel of exoglycosidases was used to selectively remove monosaccharides from glycans. All exoglycosidases were obtained from NEB except β-glucuronidase (Sigma–Aldrich). All enzymatic digestions with NEB enzymes were performed by digesting 1 to 2 μl of 2-AA-labeled glycans overnight at 37 °C using the recommended buffer (glycobuffer 1 or 4; NEB) in 10 μl total reaction volumes. The details of enzyme sources, reagents, and conditions for each reaction are listed in supplemental Table S1. For 10 μl digestions with β-glucuronidase, the enzyme was diluted 10-fold in 0.2% (w/v) sodium chloride in MQ, and 3.5 μl of this dilution was added to 2 μl of glycan together with 4.5 μl 50 mM sodium acetate, pH 5.0, and the reaction was incubated for 1 h at 37 °C. Enzyme removal was subsequently performed for all exoglycosidase digestions using C18 Millipore Zip-Tips (catalog no.: Z720046-960EA) as described previously (38).

#### MALDI-TOF–MS and MALDI-TOF–MS/MS Analysis

2-AA-labeled glycans released from different parasite life cycle stages and products of HF and exoglycosidase digestions were analyzed using MALDI-TOF–MS. MALDI-TOF–MS analysis was performed using an UltrafleXtreme mass spectrometer (Bruker Daltonics) equipped with 1 kHz Smartbeam II laser technology and controlled by the FlexControl 3.4 Build 119 (Bruker Daltonics) software. Samples were spotted on a 384-well steel polished target plate. 2-AA-labeled glycans solubilized in MQ were mixed on the plate with 2,5-dihydroxybenzoic acid matrix (catalog no.: 8201346; Bruker Daltonics; 20 mg/ml in 30% ACN), whereas products of exoglycosidase digestions were directly eluted onto the plate in 50% ACN and 0.1% TFA mixed with 2,5-dihydroxybenzoic acid (10 mg/ml) at the end of the enzyme removal with C18 Millipore Zip-Tips. All spectra were obtained in the negative-ion reflectron mode using Bruker peptide calibration mix (catalog no.: 8206195; Bruker Daltonics) for external calibration. Spectra were obtained over a mass window of *m/z* 700 to 3500 with ion suppression below *m/z* 700 for a minimum of 20,000 shots (2000 Hz) obtained by manual selection of “sweet spots”. The FlexAnalysis (version 3.4, Build 50; Bruker Daltonics) software was used for data processing, including smoothing of the spectra (Savitzky Golay algorithm, peak width: *m/z* 0.06, one cycle), baseline subtraction (Tophat algorithm), and manual peak picking. Peaks with a signal-to-noise ratio below 2 as well as known nonglycan peaks such as glucose polymers were excluded. Deprotonated masses of the selected peaks were assigned using the GlycoPeakfinder tool of the free software GlycoWorkBench (39) (version 3; June 29, 2007). The 2-AA label was taken into account as a fixed reducing-end modification, and possible glycan composition was based on available data in the literature for other related nematodes (16) (*i.e.*, 0–10 deoxyhexoses, 0–5 hexuronic acids [HexAs], 0–20 hexoses and *N*-acetylhexosamines [HexNAcs], 0–5 phosphoethanolamines, and 0–5 PC residues). A deviation of 300 ppm was allowed for initial assignment of compositions. MS/MS was performed for structural elucidation *via* fragmentation ion analysis by MALDI-TOF/TOF on selected ions using the UltrafleXtreme mass spectrometer in negative-ion mode. Information obtained from MS/MS profiles and from the various glycan sequencing procedures (*i.e.*, HF treatment and exoglycosidase digestions) were combined to determine the final glycan composition associated to the detected *m/z* values in the MALDI-TOF–MS spectra and characterize the corresponding glycan structures.

### Construction of a *B. malayi N*-linked and GSL Glycan Microarray

#### Glycan Purification Using Ultra-HPLC Fractionation

*N*-linked and GSL glycans from approximately 600 adult female worms were extracted, released, and labeled as described in the aforementioned Structural Analysis section and pooled in order to obtain a sufficient amount of glycan for microarray generation. 2-AA-labeled glycans were purified using the ultra-HPLC (UHPLC) Dionex UltiMate 3000 system from Thermo Fisher Scientific. Labeled glycans were first separated by normal phase UHPLC using hydrophilic interaction chromatography on a TSKgel Amide-80 column (4.6 mm × 15 cm, particle size 3 μm; catalog no.: 0021867; Tosoh). Eluent A consisted of 50 mmol/l formic acid (pH 4.4), whereas eluent B consisted of 100% ACN. A linear gradient from 78% to 46% eluent B was applied at a flow rate of 1 ml/min. Fluorescence detection was performed at λ_ex_–λ_em_ 250 to 425 nm. Fractions were collected, dried down in a Speedvac, redissolved in 50 μl of MQ, and analyzed by MALDI-TOF–MS as described. Then, 0.5 μl of all obtained fractions was used for analytical separation on reverse-phase (RP)-UHPLC using a C18 column (250 × 4 mm, particle size 4 μm, Superspher RP-18 endcapped; catalog no.: 116858; Millipore Sigma). Eluent A was 0.1% formic acid, whereas eluent B was 95% ACN and 0.1% formic acid. Flowrate was set at 0.5 ml/min, and gradient conditions were 5% eluent B for the first 5 min followed by a linear gradient ramping from 5 to 50% B for *t* = 5 min to *t* = 30 min. Fluorescence detection was performed at λ_ex_–λ_em_ 250 to 425 nm, and the amount of glycan in each fraction was estimated based on peak height. Fractions containing >20 pmol of glycan were subjected to a preparative RP-UHPLC separation using the same elution conditions as for the analytical run. As before, collected fractions were dried, redissolved in MQ, and analyzed by MALDI-TOF–MS.

#### Glycan Microarray Printing

A *B. malayi* glycan microarray was constructed as described previously (14, 40). To summarize, 20 pmol of collected glycan was aliquoted to 384-well V-bottom polypropylene plates (catalog no.: 784201; Greiner Bio-One). When available, 60, 200, and 600 pmol of each fraction were in addition aliquoted to the plate. The plate was evaporated in a vacuum centrifuge, and glycans were redissolved in 20 μl of 1× spotting buffer (Nexterion Spot, Nexterion; catalog no.: 1066029; Schott AG) with 10% dimethyl sulfoxide. A total of 108 glycan samples were printed in triplicate to epoxysilane-coated glass slides (Slide E, Nexterion; catalog no.: 1066643; Schott AG), leading to a total of 324 spots printed per array, using the Microgrid 600 microarrayer (Genomic Solutions) equipped with SMP3 pins that deposit 0.7 nl at each contact. Each array was printed eight times per glass slide with a 0.245 mm spacing between spots and 4.60 mm spacing between the printing areas of each array (41).

### Experimental Design and Statistical Rationale

Our newly generated glycan microarrays were screened with various samples. First, the array was validated using two mAbs before being incubated with plasma from humans and sera from rhesus macaques. Serum/plasma antibody binding from several *B. malayi*-infected and *B. malayi*-uninfected individuals was measured, and effects of infection on antibody responses were assessed using data visualization and Bayesian statistics for comparison of the different conditions.

#### mAbs

The 100-4G11-A mAb recognizing the terminal trimannosyl-branched motif Man(α1–6Man)α1–3Man present on the mannosidic glycans Man3 and Man5 was used as the first control. This mAb was produced in-house as described previously (42, 43), and its binding specificity has already been studied previously (41, 44). Next, the commercial anti-PC TEPC-15 clone (catalog no.: M1421; IgA from murine myeloma) was used to detect the filarial-specific substituent PC.

#### Biological Samples

##### Human Plasma

Human plasma studies were in accordance to the Declaration of Helsinki. Two different sets of human plasmas from individuals infected with *B. malayi* were obtained from previous studies (45, 46, 47). The study populations were residents of an Indonesian *B. malayi* endemic area. The purposes of the studies and procedures involved were explained to all participants, and only those granting informed consent were enrolled as study participants. In addition, clinical study and blood withdrawal were conducted in accordance with the guidelines of Indonesian Department of Health and Human services. Set 1 consisted of five individuals infected with *B. malayi* from the following study (45, 46, 47). Set 2 consisted of five individuals obtained from a different study (48, 49) in which patients were treated with DEC anthelminthic after initial sampling in 1990. Another blood sampling was performed almost 2 years later to address the infection status of the subjects. Thus, for each individual, we had access to two time points with different infection status: (1) subject infected (microfilariemic) before DEC treatment and (2) subject treated with DEC (amicrofilariemic). Microfilaremia data for set 1 and set 2 (pretreatment) are provided in supplemental Table S2*A*. In addition to these two sets of infection plasmas, we used plasma from five healthy European donors from Sanquin (Dutch blood bank) as nonendemic controls.

##### Rhesus Macaque Serum

A cohort of longitudinal serum samples from rhesus macaques (*Macaca mulatta*) from a previous study (50) was used to study antibody responses to *B. malayi* glycans during the course of infection. Use of macaques and the experimental procedures performed were reviewed and approved by The Institutional Animal Care and Use Committee at Bioqual, Inc and University of Illinois College of Medicine at Rockford. Humane use of animals was performed according to the guidelines for the care and use of laboratory animals and with the rules formulated under the Animal Welfare Act by the US Department of Agriculture. Additional information in terms of experimental procedures can be found in the original study (50). We used the sera of four of the animals from this cohort, which were infected by subcutaneous infection with 130 to 180 infective L3 of *B. malayi*. Serum was sampled preinfection and again at 5, 12, and 15 weeks postinfection (wpi). All infected animals showed MF in their blood by 12 wpi and impaired lymph flow when assessed at 16 wpi. These data are summarized in supplemental Table S2*B*.

#### Glycan Microarray Incubation and Data Analysis

Generated *B. malayi* glycan microarrays were first incubated with the mAbs for validation before human plasma, and rhesus macaque sera were next screened for antibody binding to glycans. The protocols for slide incubation, scanning, and data handling have been reported previously (40, 51). Briefly, residual epoxides on the glycan array slides were blocked using a solution of 2% bovine serum albumin and 50 mM ethanolamine in PBS. Slides were then incubated with plasma/sera at a 1:100 dilution or with mAbs—using a 1:500 dilution for 100-4G1 and 1:2000 for M1421. Binding of 100-4G1 to mannosidic *N*-glycans was detected with an antimouse IgM Alexa Fluor 555 (catalog no.: A-21426; Invitrogen) as secondary antibody. Antimouse Ig (polyclonal, nonconjugated; catalog no.: Z0259; Dako) and anti-rabbit IgG (heavy + light chains) Cy3 conjugate (catalog no.: A10520; Life) were preincubated for 45 min at room temperature on a shaker, and the obtained mixture was used to detect anti-PC mAb M1421. Antiglycan IgG and IgM binding from sera/plasma was detected using goat antihuman IgG (Fc-specific) Cy3 conjugate (catalog no.: C-2571; Sigma–Aldrich) and goat antihuman IgM (heavy chain) Alexa Fluor 647 (catalog no.: A-21249; Invitrogen) as secondary antibodies. For array screening with human plasma and mAbs, fluorescence was measured using a G2565BA scanner (Agilent Technologies) at 10 μm resolution using two laser channels at 532 and 633 nm and with the sensitivity level of the photomultiplier tube set at 10%. Microarray screening with the longitudinal serum from macaques was performed as described previously except imaging, which used the 4400A scanner (Genepix) with the following settings: photomultiplier tube set at 450, laser power at 70, and accuracy at 5 μm. Image analysis was processed with GenePix Pro 7.0 software (Molecular Devices) according to published methods (52). The acquired data were exported to Excel, and for each glycan-containing fraction, intensity of triplicates was averaged and corrected for background using average fluorescence intensity of “blank” spots, that is, spots printed from wells containing only spotting buffer—as a baseline. Finally, GLycan Array Dashboard (53), from the glycotoolkit Web site (glycotoolkit.com/Tools/GLAD/) hosted by the National Center for Functional Glycomics (NCFG), was used for data visualization, data mining, generation of heatmaps, and boxplot graphs.

#### Statistical Analysis

Background-corrected fluorescence intensities were normalized using log_2_ transformation, and all the subsequent statistical data analyses were conducted using the publicly available statistical programming language R (http://CRAN.R-project.org/; version 3.5.0). Significant differences between log_2_ normalized datasets were evaluated using the R/Bioconductor software package *limma*. This package, optimized for statistical analysis of microarray data, uses a linear model approach to analyze microarray experiments and empirical Bayesian methods to assess significant differences between groups (54, 55).

## Results

### Structural analysis of *B. malayi* glycans

**Fig. 1:**
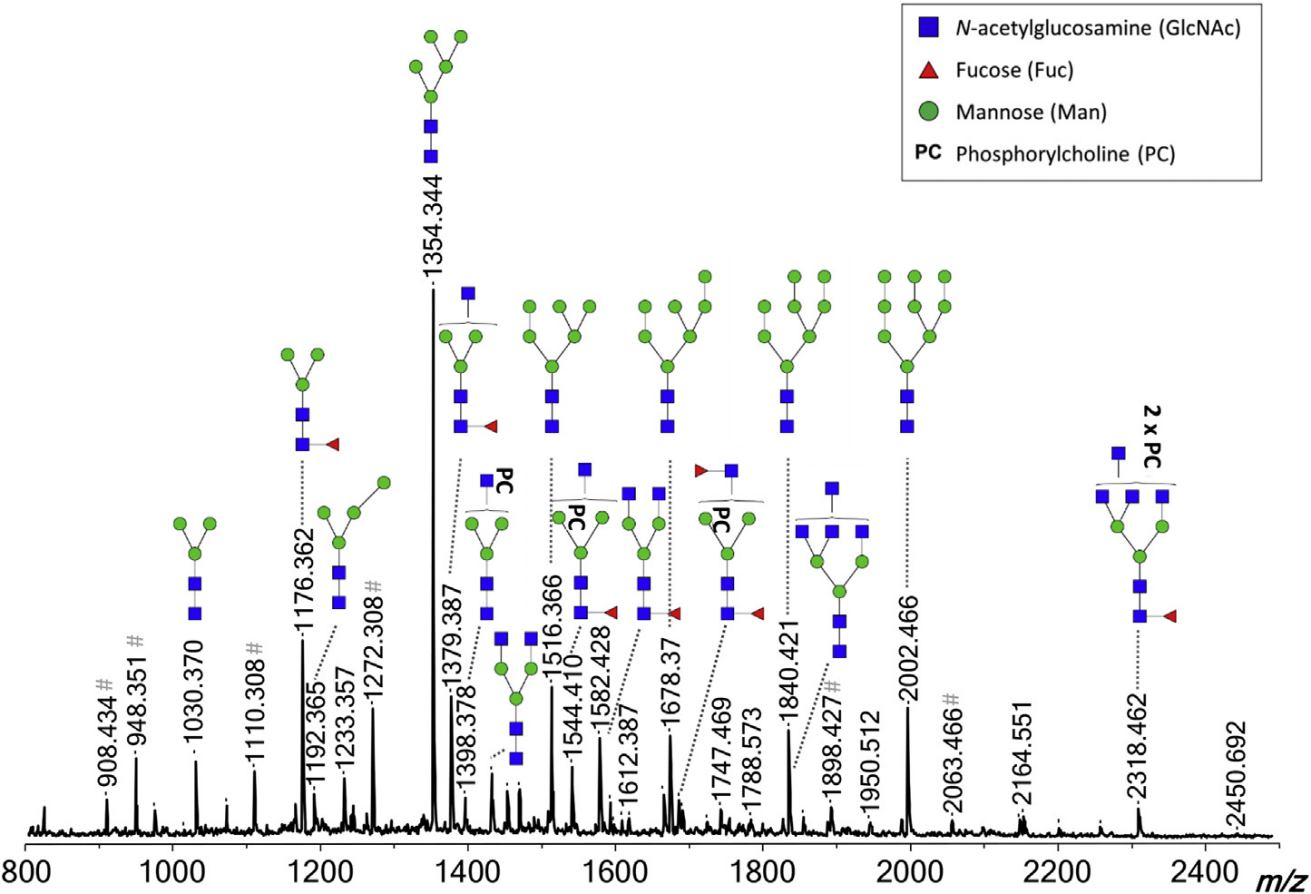
**MALDI-TOF–MS of *Brugia malayi* adult worm (mixed sex) *N*-linked glycans.** PNGase F-released glycans from *B. malayi* glycoproteins were labeled with 2-AA and analyzed using MALDI-TOF–MS in negative-ion reflectron mode. Monoisotopic masses of measured signals are indicated, and proposed glycan structures for ions with signal-to-noise ratios superior to 4 and intensities above 5000 are depicted using the Consortium for Functional Glycomics (CFG) nomenclature (see *symbol key inset*). Compositions and structures were deduced using a panel of glycan sequencing techniques in combination with MALDI-TOF–MS/MS fragmentation and information from the literature. 2-AA, 2-aminobenzoic acid; MS, mass spectrometry; MS/MS, tandem MS; PNGase F, peptide:*N*-glycosidase F.

**Fig. 2:**
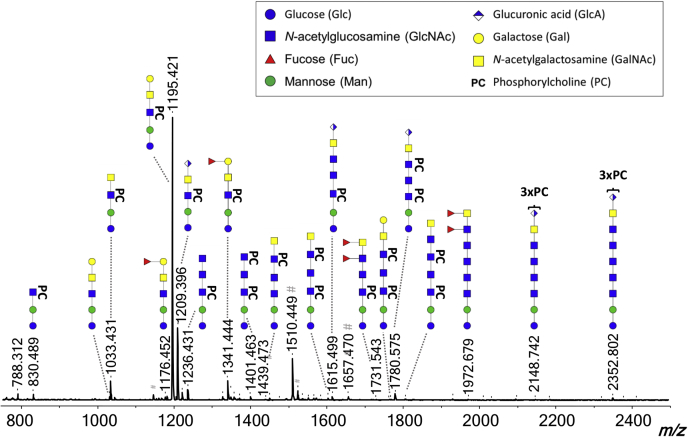
**Evidence of terminal glucuronic acid–containing *N*-glycans in *Brugia malayi*.** Negative ion reflectron mode MALDI-TOF–MS/MS of hexuronic acid–containing ions of *B. malayi N*-glycans (*A*). Ion species subjected to fragmentation analysis are indicated in the *upper left corner* of each panel. Resulting spectra are labeled with graphic representation of Y-type ions, unless indicated otherwise (B = B-type, C = C-type, and Z = Z-type). Loss of mass 59 Da from the parent ion in panel 2 is indicative of loss of a PC (56, 57) and is highlighted by a *blue double arrow*. MALDI-TOF–MS analysis of selected 2-AA-labeled *B. malayi* glucuronic acid–containing *N*-glycan before and after sequential digestion with exoglycosidases (*B*). β-glucuronidase was applied to a fraction containing UHPLC-purified *N*-glycans (undigested control in panel 1). Product of β-glucuronidase digestion was further digested with *N*-acetylglucosaminidase (panel 3) and *N*-acetylhexosaminidase (panel 4). *Blue arrows* are used to highlight digestion products. For both MALDI-TOF–MS/MS and MALDI-TOF–MS spectra, monoisotopic masses of AA-labeled glycans are indicated, and glycans are represented using the CFG nomenclature: *blue square* = GlcNAc, *green circle* = mannose, *white* and *blue diamond* = glucuronic acid, and *yellow square* = GalNAc. 2-AA, 2-aminobenzoic acid; CFG, Consortium for Functional Glycomics; MS/MS, tandem MS; PC, phosphorylcholine; UHPLC, ultra HPLC.

We analyzed *N*-glycans and GSL glycans isolated from *B. malayi* adult males, adult females, MF, and L3 larvae by MALDI-TOF–MS. Glycan compositions and structures were derived from the MALDI-TOF–MS spectra using the GlycoWorkBench software (39) in combination with selective degradation techniques, namely MS/MS fragmentation, exoglycosidase digestions, and HF treatment. Figures 1 and 2 indicate the predominant glycan structures in the mass spectra obtained for adult worm–derived (mixed sex) *N*-linked and GSL glycans, respectively. The mass spectra of all examined life stages and sexes, and a complete listing of all identified glycans and additional linkage information, are available in supplemental Fig. S1, supplemental Table S3 (*N*-glycans), and supplemental Table S4 (GSL glycans). *N*-glycan MALDI-TOF–MS profiles of the various life stages and sexes studied, particularly L3, show differences in terms of relative signal intensities (supplemental Fig. S1*A*). Although less marked, similar observations were made for GSL glycans (supplemental Fig. S1*B*). Spectra obtained for GSL glycans from L3 larvae are omitted because of the low signal intensity resulting from the low amount of starting material available. Other than in terms of relative amounts, we did not observe any major differences in the glycan structures detected. Since the glycan structures were common to various life stages, and thus, present in various spectra, we refer to glycan structures using theoretical masses throughout the article. Experimental masses from the MALDI-TOF–MS spectra can be observed in the figures and are listed in supplemental Tables S3 and S4. Highly reproducible MALDI-TOF–MS spectra were obtained for the three biological replicates studied for both adult worms and MF as shown in supplemental Fig. S1, *B* and *C* (*N*-glycans) and supplemental Fig. S1, *E* and *F* (GSL glycans). Raw data are also presented in supplemental Tables S3 and S4, respectively.

#### *N*-glycans

**Fig. 3:**
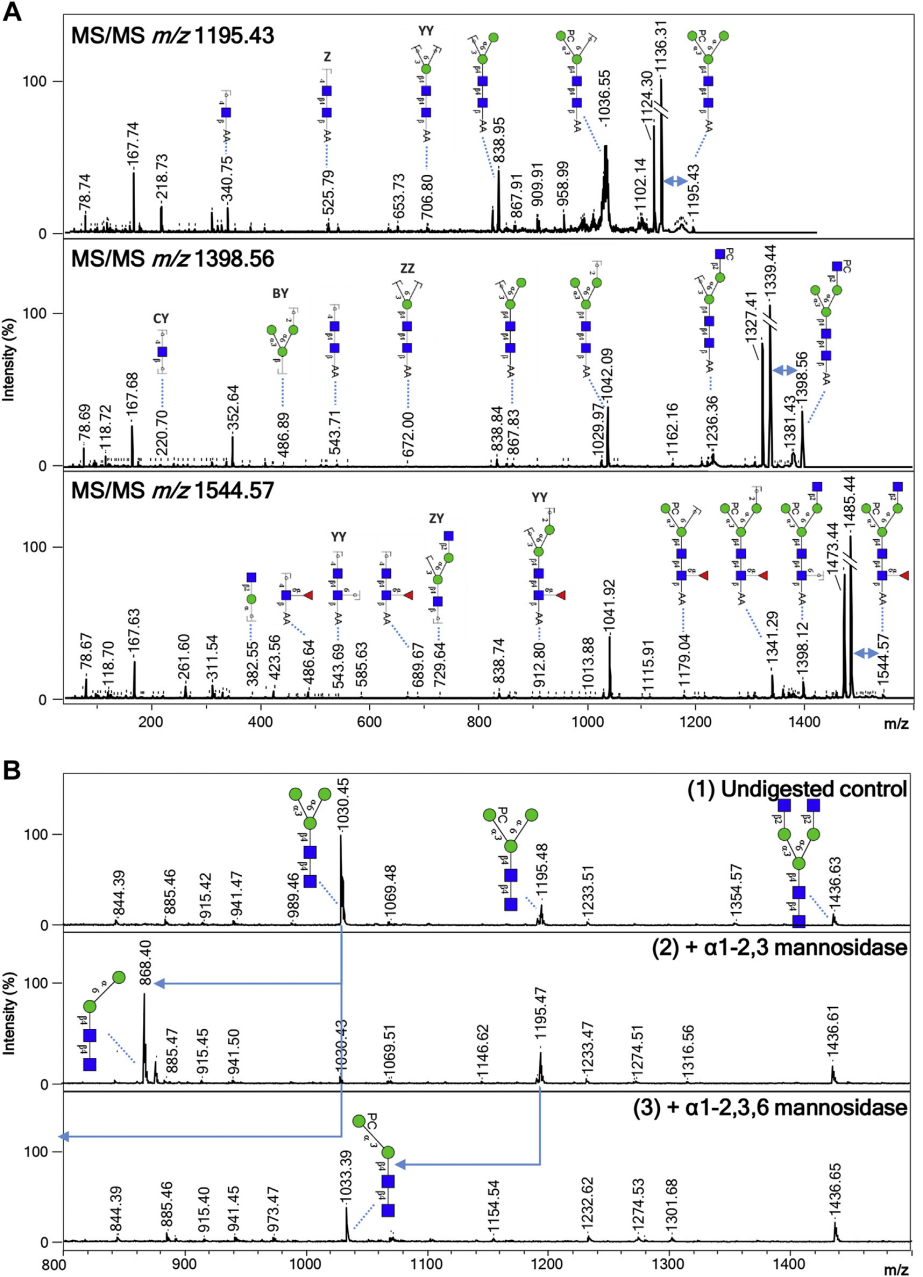
**MALDI-TOF–MS of *Brugia malayi* adult worm (mixed sex) GSL-derived glycans.** EGCase I-released glycans from *B. malayi* glycolipids were labeled with 2-AA and analyzed using MALDI-TOF–MS in negative-ion reflectron mode. Monoisotopic masses of measured signals are indicated, and proposed glycan structures for ions with signal-to-noise ratios superior to 4 and intensities above 5000 are depicted using the CFG nomenclature (see *symbol key inset*). Compositions and structures were deduced using a panel of glycan sequencing techniques in combination with MALDI-TOF–MS/MS fragmentation and information from the literature. 2-AA, 2-aminobenzoic acid; CFG, Consortium for Functional Glycomics; EGCase I, endoglycoceramidase I; GSL, glycosphingolipid; MS, mass spectrometry.

**Fig. 4:**
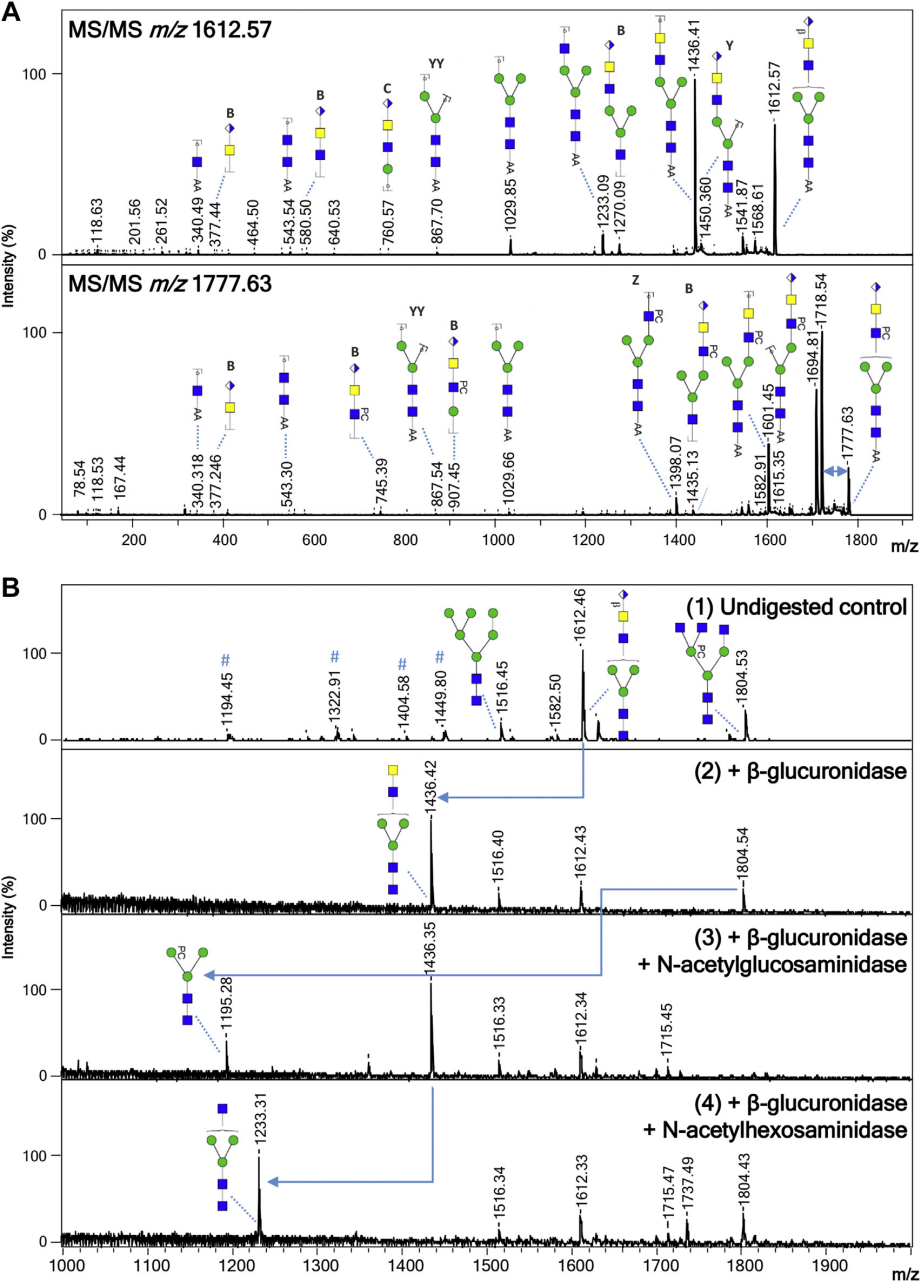
**Evidence of PC-substituted GlcNAc and core mannose in *Brugia malayi N*-glycans.** Negative ion reflectron mode MALDI-TOF–MS/MS of selected ions illustrating the different contexts of PC substitution on *B. malayi N*-glycans (*A*). Ion species subjected to fragmentation analysis are indicated in the *upper left corner* of each panel. Resulting spectra are labeled with graphic representation of Y-type ions, unless indicated otherwise (B = B-type, C = C-type, and Z = Z-type). Loss of mass 59 Da from the parent ion is indicative of a PC loss (56, 57) and highlighted by *blue double arrows*. Note that the peaks resulting from these losses were trimmed (as indicated by the *double bars*) in all three spectra because of their high intensity, to allow a better visualization of the ions in the lower *m/z* of the spectra. MALDI-TOF–MS analysis of selected 2-AA-labeled *B. malayi N*-glycans before and after digestion with the specific α1–2,3 mannosidase or the broad α1–2,3,6 mannosidase (*B*). *Blue arrows* are used to highlight digestion products. For both MALDI-TOF–MS/MS and MALDI-TOF–MS spectra monoisotopic masses of AA-labeled glycans are indicated, and glycans are represented using the CFG nomenclature: *blue square* = GlcNAc, *green circle* = mannose, and *red triangle* = fucose. 2-AA, 2-aminobenzoic acid; CFG, Consortium for Functional Glycomics; MS, mass spectrometry; MS/MS, tandem MS; PC, phosphorylcholine.

Forty-seven MALDI-TOF–MS peaks were assigned to *N*-glycan structures ranging in *m/z* values from ∼700 to 2500 [M − H]^−^ (supplemental Table S3). Most of the major signals were sensitive to mannosidase digestion (supplemental Fig. S2, *B* and *D*) and attributed to oligomannosidic structures (Man_3–9_) (*m/z* 1030.30, H_3_N_2_; 1192.42, H_4_N_2_; 1354.48, H_5_N_2_; 1516.53, H_6_N_2_; 1678.58, H_7_N_2_; 1840.64 H_8_N_2_; 2002.69, H_9_N_2_ [M − H]^−^) and fucosylated paucimannosidic structures (*m/z* 1014.38, F_1_H_2_N_2_ and 1176.43, F_1_H_3_N_2_ [M − H]^−^). Less abundant but clearly detectable were many glycans of compositions (F_1–2_)H_3_N_3–7_(PC_1–3_) (Fig. 1). Based on their sensitivity to treatment with β-*N*-acetylglucosaminidase, an enzyme able to cleave off terminal β(1–2,3,4,6) GlcNAc residues, all the terminal HexNac monosaccharides within these structures appeared to be GlcNAc (supplemental Fig. S2, *A* and *B*). Up to three of these terminal GlcNAc residues appeared to be directly linked to the trimannosyl core (*m/z* 1233.45, H_3_N_3_; 1379.51, F_1_H_3_N_3_; 1398.51, H_3_N_3_PC_1_; 1436.532, H_3_N_4_; 1525.54, F_2_H_3_N_3_; 1544.57, F_1_H_3_N_3_PC_1_; 1582.59, F_1_H_3_N_4_; 1601.59, H_3_N_4_PC_1_ 1639.61, 1690.62, H_3_N_5_; 1728.65, F_2_H_3_N_4_; 1747.65, F_1_H_3_N_4_PC_1_; 1785.67, F_1_H_3_N_5_; 1804.67, H_3_N_5_PC_1_, 1893.70, F_2_H_3_N_4_PC_1_; 1912.70, F_1_H_3_N_4_PC_2_; 1932.72, F_2_H_3_N_5_; 1950.73, F_1_H_3_N_5_PC_1_; 2096.78, F_2_H_3_N_5_PC_1_; 2115.78, F_1_H_3_N_5_PC_2_; 2261.84, F_2_H_3_N_5_PC_2_ and 2280.84 F_1_H_3_N_5_PC_3_ [M − H]^−^) as these structures were sensitive to the β(1–2,3,4,6)-*N*-acetylglucosaminidase but resistant to digestion with the β-*N*-acetylhexosaminidase that cleaves off terminal β(1–3,4,6) GlcNAc and β(1–4)-linked GalNAc monosaccharides from glycans (supplemental Table S3 and supplemental Fig. S2, *A*–*C* and *E*–*J*). The β-*N*-acetylhexosaminidase was, however, able to digest glycans with compositions (F_1–2_)H_3_N_6–7_(PC_1–3_) (*m/z* 1842.69, H_3_N_6_; 1988.75, F_1_H_3_N_6_; 2299.86, F_2_H_3_N_6_PC_1_; 2318.86, F_1_H_3_N_6_PC_2_; 2338.89, F_2_H_3_N_7_ and 2483.91, F_1_H_3_N_6_PC_3_ [M − H]^−^) by cleaving off one to two GlcNAc residues, indicating GlcNAcβ(1–4/6)–GlcNAc1 antenna within those structures (supplemental Table S3 and supplemental Fig. S2, *E* and *I*). The aforementioned glycan structures were often fucosylated with α(1–6) core fucoses (such as *m/z* 1379.51, F_1_H_3_N_3_ and 1582.59, F_1_H_3_N_4_ [M − H]^−^), which are unaffected by HF treatment (supplemental Fig. S2, *A* and *I*). On the contrary, sensitivity to HF treatment and MALDI-TOF–MS/MS analysis demonstrated the presence of outer arm fucoses α(1–3) linked to terminal GlcNAc(s) in several *N*-glycans (*e.g.*, *m/z* 1382.51, F_1_H_2_N_3_PC_1_; 1893.70, F_2_H_3_N_4_PC_1_; 2058.76, F_2_H_3_N_4_PC_2_ [M − H]^−^) (supplemental Fig. S2, *A*, *I*, *L*–*O*, and *Q*). Notably, all the core fucoses were concluded to be α(1–6) linked since all *N*-glycans were released using PNGase F. An additional experiment using PNGase A for glycan release did not reveal any additional structures, indicating that α(1–3)-linked core fucoses are not present in *B. malayi N*-glycans (data not shown). PC substitutions were detected for a broad range of *N*-glycans, often in combination with core fucosylation (*e.g.*, *m/z* 1341.49, F_1_H_3_N_2_PC_1_; 1544.57, F_1_H_3_N_2_PC_1_; 1747.65, F_1_H_3_N_4_PC_1_; 1912.70, F_1_H_3_N_4_PC_2_; 1950.73, F_1_H_3_N_5_PC_1_; 2115.78, F_1_H_3_N_5_PC_2_; 2280.84, F_1_H_3_N_5_PC_3_; 2483.92, F_1_H_3_N_6_PC_3_ [M − H]^−^) and with both core and outer-arm fucosylations (*e.g.*, *m/z* 1893.70, F_2_H_3_N_4_PC_1_; 2058.76, F_2_H_3_N_4_PC_2_; 2261.84, F_2_H_3_N_5_PC_2_ [M − H]^−^) (supplemental Table S3 and supplemental Fig. S2, *A* and *I*). When present, the PC substitution was largely found on the terminal GlcNAc residues (*e.g.*, *m/z* 1398.51, H_3_N_3_PC_1_; 1893.70, F_2_H_3_N_4_PC_1_; 1912.70, F_1_H_3_N_4_PC_2_; 2058.75, F_2_H_3_N_4_PC_2_; 2096.78, F_2_H_3_N_5_PC_1_; 2115.78, F_1_H_3_N_5_PC_2_; 2261.83, F_2_H_3_N_5_PC_2_ [M − H]^−^) (supplemental Table S3) as shown by resistance to β-*N*-acetylglucosaminidase digestion of the PC-bearing GlcNAc and MS/MS analysis (as exemplified for ion of *m/z* 1398.50 in Fig. 3*A*). Interestingly however, PC also appeared to be present on the trimannosyl core of several *N*-glycans (*e.g.*, *m/z* 1341.49, F_1_H_3_N_2_PC_1_; 1382.51, F_1_H_2_N_3_PC_1_; 1544.57, F_1_H_3_N_3_PC_1_; 1601.59, H_3_N_4_PC_1_; 1747.65, F_1_H_3_N_4_PC_1_; 1804.67, H_3_N_5_PC_1_; 1950.73, F_1_H_3_N_5_PC_1_ [M − H]^−^) as indicated by the complete digestion down to the trimannosyl core by β-*N*-acetylglucosaminidase treatment of these structures (exemplified for *m/z* 1544.57; 1601.59; 1747.65; 1804.67; 1950.73 [M − H]^−^ in supplemental Fig. S2, *E*, *G*, *H*, and *J*). The occurrence of PC on the *N*-glycan core was confirmed by subjecting the *m/z* 1195.43 [M − H]^−^, 1398.32 [M − H]^−^, and 1544.33 [M − H]^−^ ions to MALDI-TOF–MS/MS fragmentation analysis (Fig. 3*A*). For each of the three structures, we detected a marked loss of 59 Da, a fragment ion resulting from the neutral loss of N(CH_3_)_3_ observed for PC-modified peptides (56, 57), as well as the less visible losses of masses 165 Da (PC) and 183 Da (PC + H_2_O). The mass difference of 369 Da, which corresponds to the loss of a PC-substituted HexNAc, was only found in the fragmentation spectrum of the *m/z* 1398.32 [M − H]^−^ ion, and its direct loss from the parent mass indicated the terminal position of the HexNAc–PC group. However, for ion species 1195.43 [M − H]^−^ as well as 1544.33 [M − H]^−^, the observed loss of 328 Da matched the mass of a PC-substituted hexose. Its terminal position deduced from the fragmentation spectrum of 1195.43 [M − H]^−^ indicated that the PC substitutes one of the terminal mannoses. Digestions of selected structures with specific mannosidases indicated that the PC is linked to the α1–3-linked mannose of the *N*-glycan core (Fig. 3*B*). Finally, we identified two ions representing less abundant *N*-glycans, which contained a HexA residue (H_3_N_4_A_1_ with *m/z* 1612.56 and H_3_N_4_A_1_PC_1_ with *m/z* 1777.63 [M − H]^−^). MS/MS analysis (Fig. 4*A*) and resistance to both β-*N*-acetylglucosaminidase and β-*N*-acetylhexosaminidase of these structures (supplemental Fig. S2*J*) suggested that the HexA residue caps a HexNAc–HexNAc antenna, which most likely consists of a GalNAcβ1–4GlcNAc (LacdiNAc [LDN]) unit frequently observed in helminths (58, 59, 60). The HexA residue in H_3_N_4_A_1_ was sensitive to β-glucuronidase treatment, indicating that it is a β-linked glucuronic acid (GlcA) residue. The β-glucuronidase digestion product (*m/z* 1436.53, H_3_N_4_ [M − H]^−^) could be further digested to *m/z* 1233.31, H_3_N_3_ [M − H]^−^ with β-*N*-acetylhexosaminidase, but not with β-*N*-acetylglucosaminidase, confirming that the GlcA appears to be β-linked to an LDN motif (Fig. 4*B*). Finally, MS/MS analysis of the H_3_N_4_A_1_PC_1_ ion at *m/z* 1777.63 suggests that an additional PC substituent is attached to the mannose-linked GlcNAc residue in this structure (Fig. 4*A*). In summary, our structural characterization of *B. malayi N*-glycans highlights a number of common helminth traits, including truncated structures, core fucosylation, paucimannosidic glycans, and high-mannosidic glycans as well as some more filarial-characteristic features, such as PC substitution, GlcNAc-constituted antennae, and terminal GlcA.

#### GSL Glycans

**Fig. 5:**
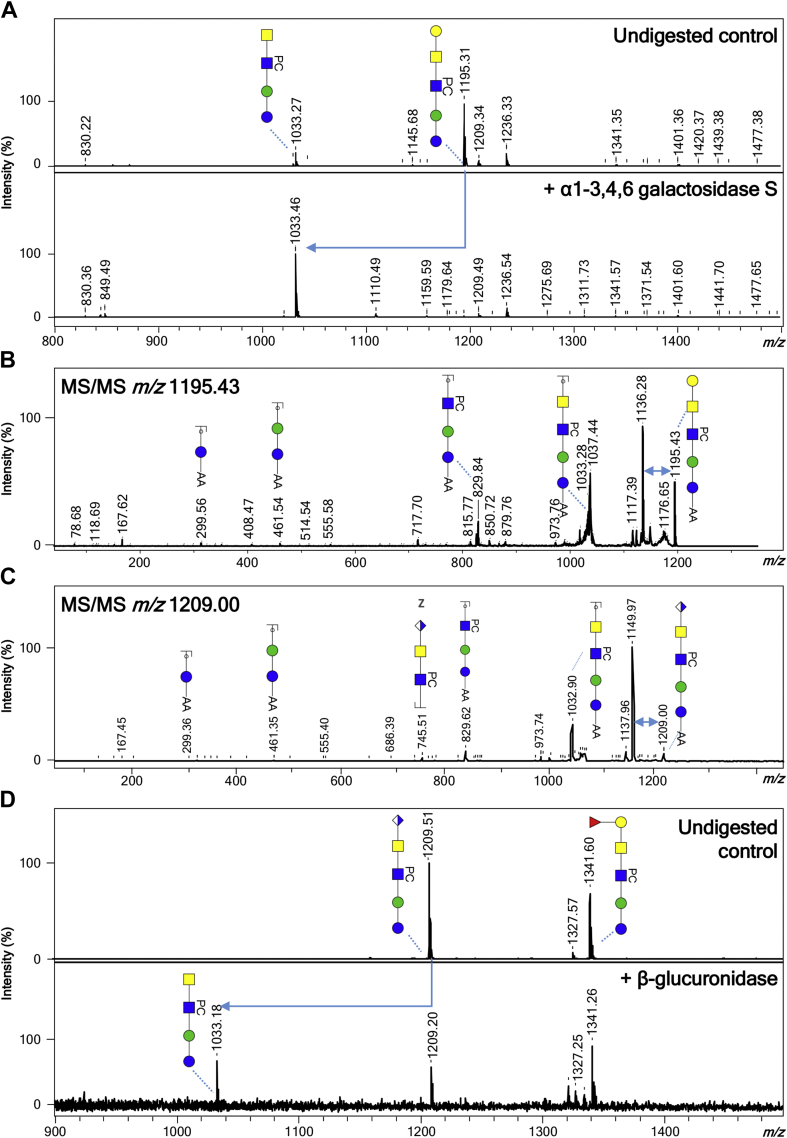
**MALDI-TOF–MS/MS analysis and exoglycosidase digestions of *Brugia malayi* most abundant GSL glycan species.** MALDI-TOF–MS analysis of 2-AA-labeled *B. malayi* major GSL glycan species subjected to galactosidase (*A*). Treatment with α1–3,4,6 galactosidase affected ion with *m/z* 1195.38 [M − H]^−^ resulting in the loss of a terminal α-galactose (Δ*m/z* = 162) as indicated by the *blue arrow*. MALDI-TOF–MS/MS of the two major GSL glycan ion species (*B* and *C*) ion species with *m/z* 1195.38 [M − H]^−^ (*B*) and 1209.36 [M − H]^−^ (*C*) were subjected to MS/MS fragmentation analysis. Resulting spectra are labeled with graphic representation of Y-type ions, unless indicated otherwise (B = B-type, C = C-type, and Z = Z-type). Loss of mass 59 Da from parent ion is indicative of a PC loss (56, 57) and is highlighted by *blue double arrows*. MALDI-TOF–MS analysis of 2-AA-labeled *B. malayi* major glucuronic acid–containing GSL glycan species subjected to β-glucuronidase. *D*, ion *m/z* 1209.36 [M − H]^−^ was partially affected by treatment with β-glucuronidase resulting in the loss of a terminal β-glucuronic acid (Δ*m/z* = 176) as indicated by the *blue arrow*. For both MALDI-TOF–MS and MS/MS spectra, measurements were acquired in negative-ion reflectron mode, signals are labeled with monoisotopic masses of AA-labeled glycans, and glycans are represented using the CFG nomenclature: *blue square* = GlcNAc, *green circle* = mannose, *red triangle* = fucose, *white and blue diamond* = glucuronic acid, *yellow circle* = galactose, and *yellow square* = GalNAc. 2-AA, 2-aminobenzoic acid; GSL, glycosphingolipid; MS, mass spectrometry; MS/MS, tandem MS; PC, phosphorylcholine.

GSL glycan structures were assigned to 47 different peaks in the *B. malayi* lipid glycan MALDI-TOF–MS spectrum in the range of *m/z* = 700 to 2500 [M − H]^−^ (Fig. 2 and supplemental Table S4). Based on the literature (16), filarial nematode GSLs are built on the GSL arthrotype core GlcNAcβ1–3Manβ1–4Glc where the glucose is attached to the ceramide portion. The *B. malayi* GSL glycan spectrum is dominated by a major ion of *m/z* 1195.43 [M − H]^−^ (Fig. 2) composed of three hexoses, two HexNAc residues, and a PC substituent (H_3_N_2_PC_1_). Digestion by α(1–3,4,6) galactosidase and resistance to digestion by other exoglycosidases tested, including α(1–3,6) galactosidase, suggests that the nonreducing terminal hexose is an α(1–4)-linked galactose (Gal) (Fig. 5*A* and supplemental Fig. S3*A*). The product of the α(1–3,4,6) galactosidase treatment could be further digested to a PC-substituted arthrotype core using β-*N*-acetylhexosaminidase but not using the more specific β(1–3,4,6)-*N*-acetylglucosaminidase, which confirms the presence of a terminal β(1–4)-linked GalNAc linked to GlcNAc (supplemental Fig. S3*E*). Moreover, these digestion data in combination with MS/MS fragmentation (Fig. 5*B*) showed that the PC substituent is on the GlcNAc of the arthrotype core. Altogether, this indicates that the major GSL structure is Galα1–4GalNAcβ1–4GlcNAcβ1–3Manβ1–4Glc with the GlcNAc substituted with PC. Truncated versions of this major structure were also found (*m/z* 830.30, H_2_N_1_PC_1_; 1033.38, H_2_N_2_PC_1_ [M − H]^−^) (Fig. 2 and supplemental Fig. S3, *A*, *B*, and *D*), as well as a structure with the same Galα1–4GalNAcβ1–4GlcNAcβ1–3Manβ1–4Glc backbone but without the PC substituent (*m/z* 1030.37, H_3_N_2_ [M − H]^−^) (supplemental Fig. S3*B*). In addition, treatment with HF (supplemental Fig. S3*F*) showed the presence of an α1–2/3-linked or 4-linked fucose in the structure of composition F_1_H_3_N_2_PC_1_ with *m/z* 1341.49 [M − H]^−^. This fucose substituent appeared to be attached to the terminal α1–4 Gal as shown by resistance to galactosidase digestions (supplemental Fig. S3, *A* and *C*) and MS/MS analysis of this ion species (supplemental Fig. S3*L*). Similar observations were made for *m/z* 1176.43 (F_1_H_3_N_2_) (supplemental Fig. S3*B*) and 1322.49 (F_2_H_3_N_2_) [M − H]^−^ in terms of resistance to galactosidase digestions suggesting fucosylation of the terminal α-Gal for these structures as well. This resulted in a difucosylated structure for *m/z* 1322.49, F_2_H_3_N_2_ [M − H]^−^ that presents a fucosylation of the backbone-GalNAc in addition of the fucosylated α-Gal as demonstrated by MS/MS fragmentation (supplemental Fig. S3*K*). Similar to *N*-glycans, HexA residues were also detected in the GSL glycans but as a predominant feature. Notably, HexA was found as the terminal monosaccharide on the second most major structure of the spectrum (*m/z* 1209.41 [M − H]^−^) using MS/MS fragmentation analysis (Fig. 5*C*). Its resistance to all exoglycosidases tested (supplemental Fig. S3*A*), except for β-glucuronidase (Fig. 5*D* and supplemental Fig. S3*F*), suggested that, as for the *N*-glycans, terminal β-linked GlcA is present. Although we did not obtain a complete enzymatic digestion of the GlcA residue—nor for the *N*-linked glycan (*m/z* 1612.56 [M − H]^−^, Fig. 4*B*) or for other GSL glycans—the absence of sensitivity to other exoglycosidases (supplemental Figs. S2*J* and S3, *A* and *F*) and the UHPLC profiles of the corresponding fractions (data not shown) do not suggest the presence of isomeric structures. These indications strengthen our assumption of this acidic monosaccharide invariably occupying the terminal position on the glycan backbone when present. Based on our observation that the GlcA is linked to a GalNAc residue in the *N*-glycans and that the GSLs contain terminal LDN motifs, we assigned the peak at *m/z* 1209.41 [M − H]^−^ to the GlcAβGalNAcβ1–4(PC)GlcNAcβ1–3Manβ1–4Glc structure. Several other peaks present in the spectrum appeared to contain terminal GlcA on various backbone lengths ranging from 2 (*m/z* 1209.41, H_2_N_2_PC_1_A_1_ [M − H]^−^) to up to seven HexNAc stretches (*m/z* 2370.86 H_2_N_7_PC_1_A_1_ [M − H]^−^). These HexNAc residues were also frequently substituted with PC (*e.g.*, *m/z* 1615.57, H_2_N_4_PC_1_A_1_; 1780.62, H_2_N_4_PC_2_A_1_; 1818.65, H_2_N_5_PC_1_A_1_; 1945.68, H_2_N_4_PC_3_A_1_; 2021.73, H_2_N_6_PC_1_A_1_; 2351.84, H_2_N_6_PC_3_A_1_ [M − H]^−^) (supplemental Fig. S3, *F*, *I*, *J*, *M*, and *N* and supplemental Table S4), and sometimes with fucoses (*m/z* 2332.84, F_1_H_2_N_6_PC_2_A_1_; 2370.86, F_1_H_2_N_7_PC_1_A_1_ [M − H]^−^) (supplemental Table S4). In addition, we detected GSL glycans of composition H_2_N_3–8_, containing linear HexNAc stretches not terminated by GlcA, α-Gal, or fucosylated α-Gal motifs (Fig. 2 and supplemental Table S4). Comparison of treatments with both β-*N*-acetylglucosaminidase and β-*N*-acetylhexosaminidase indicated that some of these structures contained HexNAc stretches of unsubstituted GlcNAc monosaccharides (*m/z* 1477. 56, H_2_N_5_ [M − H]^−^) (supplemental Fig. S3*H*), most likely β(1–4)-linked, but more often these GlcNAc, stretches were substituted with one or several PCs such as in glycans with composition H_2_N_3–5_PC_1–2_ observed at *m/z* 1236.40, H_2_N_3_PC_1_; 1401.41, H_2_N_3_PC_2_ and 1642.61, H_2_N_5_PC_1_ [M − H]^−^ (supplemental Fig. S3, *C*, *G*, and *H*). Some GlcNAc residues were substituted with terminal fucoses (*e.g.*, *m/z* 2378.91, F_2_H_2_N_8_; 2467.95, F_4_H_2_N_7_ [M − H]^−^), blocking the β-*N*-acetylhexosaminidase activity, and a combination of both PC and fucose was detected on *m/z* 1788.67, F_1_H_2_N_5_PC_1_ [M − H]^−^ (supplemental Table S4). Finally, these HexNAc chains could also be terminated by β(1–4)-linked GalNAc as inferred from resistance to β-*N*-acetylglucosaminidase digestion and sensitivity to β-*N*-acetylhexosaminidase (supplemental Fig. S3, *B*, *C*, *G*, and *H*), confirming the presence of terminal LDN motifs mentioned previously in *B. malayi* GSL glycans. These terminal LDN-containing structures also encompassed (terminal) fucosylations (*m/z* 1420.54, F_1_H_2_N_4_; 1566.60, F_2_H_2_N_4_; 1623.62, F_1_H_2_N_5_; 1769.67, F_2_H_2_N_5_; 1826.70, F_1_H_2_N_6_; 2118.11, F_3_H_2_N_6_ [M − H]^−^) (supplemental Table S4) or PC substitutions (*m/z* 1439.53, H_2_N_4_PC_1_; 1604.59, H_2_N_4_PC_2_; 1807.67, H_2_N_5_PC_2_; 2010.75, H_2_N_6_PC_2_ [M − H]^−^) (supplemental Fig. S3, *G*, *H*, and *O* and supplemental Table S4), or both (*m/z* 1585.59, F_1_H_2_N_4_PC_1_; 1731.65, F_2_H_2_N_4_PC_1_; 1750.65, F_1_H_2_N_4_PC_2_; 1953.75, F_1_H_2_N_5_PC_2_; 2302.86, F_2_H_2_N_4_PC_2_ [M − H]^−^) (supplemental Table S4). A combination of MALDI-TOF–MS/MS (exemplified in Fig. 5 for structures with *m/z* 1195.43 [M − H]^−^ and 1209.36 [M − H]^−^) and hexosaminidase digestions was used to identify PC positions on the HexNAc backbones. Additional MALDI-TOF–MS/MS spectra can be found in supplemental Fig. S3, *K*–*O*.

### Glycan Microarray

#### Microarray Construction and Validation

2-AA-labeled *N*-glycans and GSL glycans obtained from 600 adult *B. malayi* worms were purified through a two-dimensional UHPLC protocol consisting of a first separation using hydrophilic interaction chromatography followed by a C18 RP separation. After RP-UHPLC fractionation, 36 *N*-glycan fractions and 17 GSL glycan fractions contained sufficient material (>20 pmol of total glycan) for inclusion on the microarray. Glycans present in each fraction were confirmed by MALDI-TOF–MS in combination with glycomic analysis data outlined previously. Glycan concentration was based on the calibrated fluorescence signal for each RP fraction (see Experimental Procedures for details). In addition, aliquots of four GSL glycan fractions treated with HF to remove α1–2,3,4-linked fucoses and PC residues, leaving only the unsubstituted GSL glycan backbones, were added to the array selection. All fractions thus obtained were printed on epoxy-coated glass slides at a glycan concentration of 1 μM, and when sufficient material was available also at higher concentrations of 3, 10, and 30 μM. All samples were printed on the array in triplicate. Detailed content of each fraction and the various concentrations of glycan printed are provided in supplemental Table S5. Validation of the array was performed using two mAbs. First, 100-4G11 clearly bound all *N*-glycan fractions containing Man3 (supplemental Fig. S4*A*). Recognition of Man5 was also observed, while no binding to the rest of the array was detected, consistent with the mAb specificity (41, 44). PC substituents were detected in abundance on *B. malayi* glycans, in particular on GSL glycans so that all GSL fractions contained PC-bearing structures. This was confirmed by the binding of the mAb M1421 to virtually the totality of the GSL fractions except the ones treated with HF, that is, with PC removed (supplemental Fig. S4*B*). For the *N*-glycans, binding was also very consistent with our structural findings. Interestingly, the mAb M1421 was able to bind to *N*-glycans both with PC-substituted GlcNAc(s) and with PC-substituted mannose. However, certain *N*-glycan fractions containing PC-substituted mannose were not recognized by the mAb (namely fractions Ng10, Ng13, Ng15, Ng17, Ng23, Ng30, Ng31, Ng32, and Ng33). This can be explained by the fact that all these fractions have a content of PC-substituted structures lower than 25% of the total glycan content (supplemental Table S5), showing the necessity of a minimal amount/purity for the mAb to bind to the PC substituent on the array. Overall, this validation clearly confirmed the print quality of both glycan types. Raw experimental data can be found in supplemental Table S6.

#### Antibodies Against *B. malayi*-derived Glycans Are Induced in Infected Hosts

##### IgG and IgM in the Course of Infection of *B. malayi*-infected Macaques

**Fig. 6:**
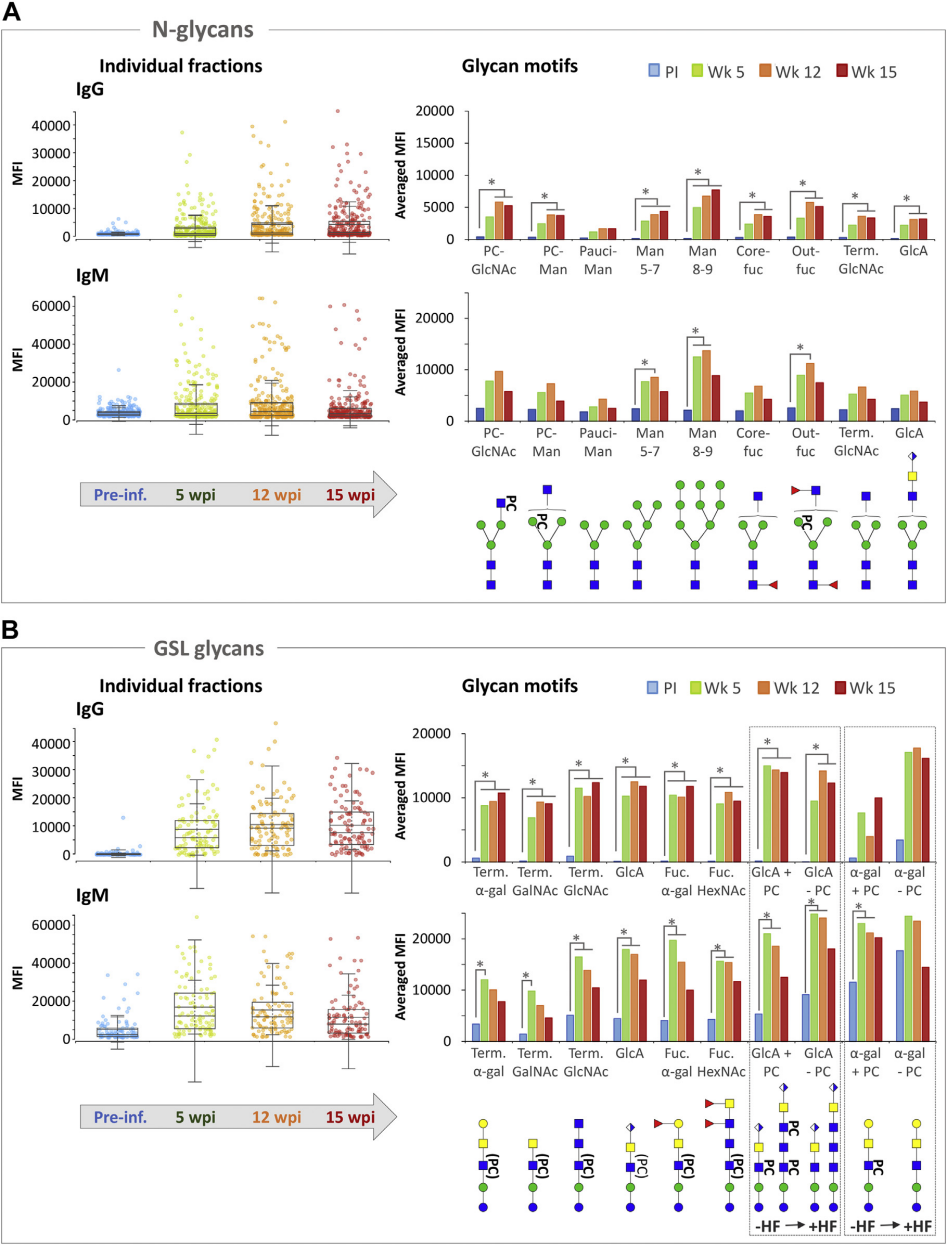
**Serum IgG and IgM responses to parasite glycans from infected rhesus macaques during establishment of *Brugia malayi* infection.** Glycan microarrays were screened with serum from a cohort of four rhesus macaques at four different time points, namely preinfection, 5 weeks post-infection (wpi), 12 wpi, and 15 wpi. IgG and IgM binding to *B. malayi* printed *N*-glycans (*A*) and GSL glycans (*B*) were measured for all four animals at each time point, and background-corrected median fluorescence intensities (MFIs) are shown. Boxplots on the *left* show the IgG (*top graphs*) and IgM (*bottom graphs*) responses to glycans at each time point, according to the following color code: *blue* = preinfection, *green* = 5 wpi, *orange* = 12 wpi, and *red* = 15 wpi. Each single dot corresponds to the Ig binding to a particular GSL or *N*-glycan-containing fraction at a specific concentration for one animal. Thus, n_dots_ = 256 (64 fractions &times 4 animals) for *N*-glycans and n_dots_ = 100 (25 × 4) for GSL glycans. Averaged MFIs to specific glycan motifs, based on glycan content of the fractions, were calculated for all time-points and are shown on the graphs on the right. Fraction groups are shown on the *x*-axis and named after the corresponding glycan motif present in the fractions: Core-fuc, α1–6 core fucosylated *N*-glycans; Fuc. α-gal, fucosylated (terminal) α-1–4 Gal; Fuc-HexNAc, fucosylated HexNAc; Man x–y, Man_x–y_ (high-mannosidic *N*-glycans carrying 5–7 or 8–9 mannose residues); Out-fuc, α1–3 fucose attached to terminal GlcNAc; pauciman, paucimannosidic *N*-glycans; PC-GlcNAc, PC-substituted GlcNAc; PC-Man, PC-substituted mannose; Term. α-Gal, terminal α1–4 Gal; Term. GlcNAc, terminal GlcNAc; Term. GalNAc, terminal GalNAc; Term. GlcA, terminal GlcA. A representative glycan structure of each category is shown below the *x*-axis to illustrate the glycan feature in question. *Gray insets* on the right compare averaged MFIs of selected GSL fractions with and without HF treatment, that is, with PC substituents removed as illustrated below the *x*-axis, where structures contained in the fractions are represented. Significant differences between time points were assessed using Bayesian statistics and are represented using *gray asterisks* and connecting lines. GSL, glycosphingolipid; Ig, immunoglobulin; PC, phosphorylcholine.

To investigate the induction of specific antiglycan IgM and IgG upon *B. malayi* infection, we incubated the constructed glycan microarrays with sera from rhesus macaques sampled during primary exposure and infection with *B. malayi* (50). We incubated sera from four different animals collected before infection (preinfection time point) and at 5, 12, and 15 wpi with *B. malayi* L3 larvae. All four animals were microfilariemic at 12 and 15 wpi—but not at 5 wpi (supplemental Table S2*B*). After data processing and correction for background, substantial median fluorescence intensity (MFI) values were detected for IgM, but not for IgG, to the printed *B. malayi*-derived glycans at the preinfection time point (Fig. 6 and supplemental Fig. S5*A*), similar to previous observations made for *Schistosoma*-infected rhesus macaques (61). Signals for both IgM and IgG however increased substantially at 5 wpi compared with baseline, as shown in Figure 6, *A* and *B*. This was observed for all animals (supplemental Fig. S5*A* and supplemental Table S6) and confirmed using Bayesian statistics of the R package *limma* for comparison of a limited number of different time points. Indeed, at 5 wpi, IgG binding was significantly higher (with α = 0.05) than preinfection for 12 different glycan fractions (supplemental Fig. S5*B*), whereas IgM binding to 17 different fractions was significantly higher (supplemental Fig. S5*C*). The number of glycans to which IgG was triggered by infection seemed to expand gradually over time, with 32 fractions becoming positive for IgG at 15 wpi. In addition, fluorescence signals to fractions already positive at 5 wpi further increased up to the 15 wpi time point (Fig. 6 and supplemental Fig. S5*B*). For IgM against *B. malayi* glycans, the highest fluorescence levels were observed earlier than the maximum IgG response, with a peak at 5 wpi (Fig. 6). Although MFI values were still higher for 15 fractions at 12 wpi compared with preinfection, IgM returned close to preinfection levels at 15 wpi (supplemental Fig. S5*C*). While a large proportion of the GSL glycans on the array became positive for both IgG and IgM, a smaller proportion of *N*-glycans was bound by the antibodies of *B. malayi*-infected rhesus macaques. This indicates that most GSL glycans, but only few *N*-glycans, are antigenic. To investigate which glycan motifs contained in the *B. malayi* glycans were specifically targeted by the host antibodies, we grouped the fractions according to their glycan structural features. For the *N*-glycans, we pooled data of glycans that are substituted with PC (with a distinction between PC-substituted mannose and PC-substituted GlcNAc), core fucosylated, fucosylated on the outer arm, terminated with GlcNAc, terminated with GlcA or containing paucimannosidic (Man_2–4_), and high-mannosidic glycans (split between Man_5–7_ and Man_8–9_) (Fig. 6*A*). For the GSL glycans, we combined data of those bearing terminal α-Gal, terminal GalNAc, terminal GlcNAc, terminal GlcA, fucosylated terminal α-Gal, and fucosylated terminal HexNAc residue(s) (Fig. 6*B*). Note that one fraction can belong to more than one category when the specific glycan contained in the fraction presents more than one of the aforementioned traits or when the printed fraction contains more than one glycan structure (supplemental Table S5). As mentioned previously, all GSL fractions contained PC-bearing structures. Thus, in order to evaluate the contribution of PC to antibody recognition of GSL glycans, we treated four fractions with HF to remove PC, three fractions containing terminal GlcA, and one fraction containing terminal α-Gal. Averaged MFI values of all fractions in a particular category are given in Figure 6. In all defined GSL categories, we observed the same dynamics as previously described, with a clear induction of IgG and IgM at 5 wpi and a gradual decline for IgM at the later time points, whereas IgG showed a further increase at 12 and 15 wpi for all antigenic motifs. In addition, for both IgG and IgM binding to the GlcA-containing glycan fractions, we observed only limited differences between native and HF-treated glycans, as shown in Figure 6*B* (*boxed insets* at the right of the graphs). This indicates that in these glycans, the PC substituents are not immunodominant and that IgG and IgM most likely bind the terminal GlcA epitopes. Also for the α-Gal-containing fraction, appreciable IgG and IgM binding was still observed after HF treatment, indicating the presence of antibodies against the terminal α-Gal motif. However, there were no significant differences between preinfection and infection time points, suggesting that antiglycan antibodies binding to this α-Gal motif, and particularly IgMs, were also present in uninfected rhesus macaque plasma and are not (solely) induced upon *B. malayi* infection.

For the *N*-glycans, we observed a weaker and more diverse Ig response than for the GSL glycans. IgG binding to most *N*-glycan motifs, including antenna with fucosylation and PC substitution, appeared later during infection with significant differences to the preinfection time point only from 12 wpi. A response to high-mannosidic glycans (Man_5–9_) was already observed at 5 wpi. IgM response to Man_5–9_ and structures with fucosylated antennae also gave significantly higher fluorescence signals at 12 wpi. Both IgG and IgM responses to *N*-glycans appeared at later time points than to GSL glycans. For *N*-glycans containing PC-substituted GlcNAc, we observed slightly higher MFIs than for the ones containing PC-substituted mannose, which might be attributed to a difference in substituent accessibility, although this trend was not clearly observed for the PC-specific mAb M1421. Hence, while higher binding to GSL glycans may be attributed to better accessibility of substituents on a linear GSL backbone than to branched *N*-glycans, it appeared to be caused mainly by the abundance of terminal antigenic motifs such as GlcA and α1–4 Gal, not present or similarly represented in *N*-glycans. This interpretation is corroborated by the similar range of MFI levels observed for binding of the mAb M1421 to *N*-linked and GSL glycans.

##### IgG Responses in Human *B. malayi* Infection

**Fig. 7:**
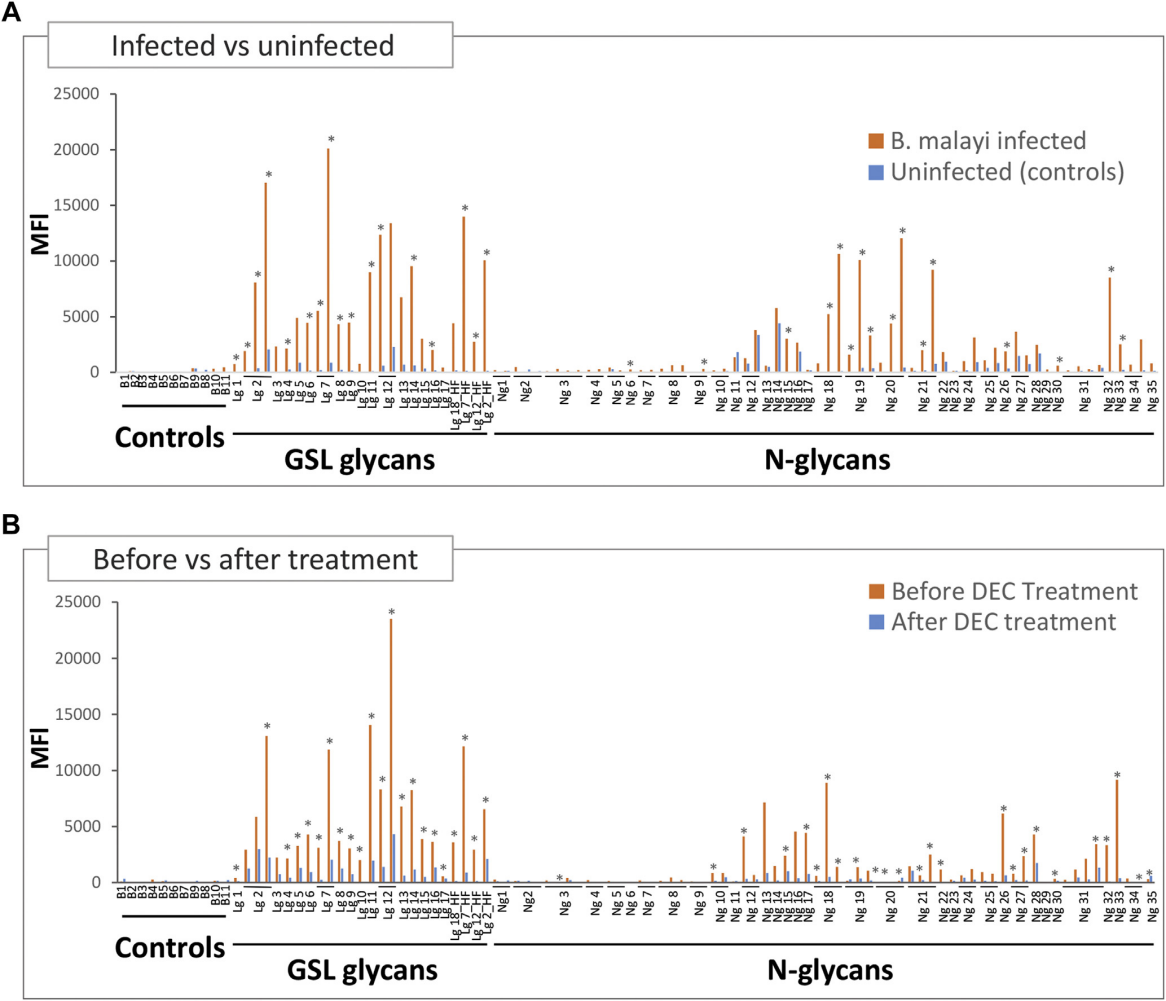
**Plasma IgG responses to *Brugia malayi* glycans of healthy, treated, and infected individuals.** Glycan microarray was screened with plasma from uninfected donors (n = 5) and set 1 of plasma from *B. malayi*-infected individuals (n = 5) (*A*) and with set 2 of plasma from *B. malayi*-infected individuals (n = 5) obtained pretreatment and after DEC chemotherapy (*B*). Median fluorescence intensities (MFIs) from IgG binding were corrected for background and averaged for each group of individuals (*A*) and for the two different time points (*B*). Each bar corresponds to antibody binding to individual glycan fractions printed on the glycan microarray. Fraction names are shown on the *x*-axis. When several concentrations were printed on the array, they are grouped under the fraction name and ranked from lowest to highest. Refer to supplemental Table S5 for exact fraction compositions and printed concentrations. The type of glycan content in the fraction is indicated below the fraction names as follows: controls = negative controls (no glycans printed), GSL glycans, or *N*-glycans. Significant differences between groups were assessed using Bayesian statistics. *p* Values <0.05 that indicate a significant difference in MFI values between groups of individuals (*A*) and between pretreatment and post-treatment time points (*B*) are represented using *gray asterisks*. DEC, diethylcarbamazine citrate; GSL, glycosphingolipid; Ig, immunoglobulin.

In order to study human antibody responses to *B. malayi* glycans, we screened our glycan microarray with a set of human *B. malayi* infection plasmas (set 1, see Experimental Procedures section). Focusing on IgG, and applying the R package *limma* for statistical analysis, responses were significantly higher in the *B. malayi*-infected group compared with the population of healthy donors used as controls (Fig. 7*A* and supplemental Table S7). The statistic *F* test highlighted that IgG fluorescence signals to 16 *N*-glycan samples out of the 64 printed on the microarray, and 17 GSL glycan samples out of 25, show significant differences between *B. malayi*-infected individuals and the uninfected donors (with significance set at *p* < 0.05) (Fig. 7*A*).

The same analysis was then conducted to study human IgM binding to *B. malayi* glycans. Overall, antiglycan IgM responses did not exceed background levels, and no clear differences between control and infected individual profiles could be observed (data not shown). Consequently, we decided to focus on human IgG responses to *B. malayi* glycans for the rest of the study.

**Fig. 8:**
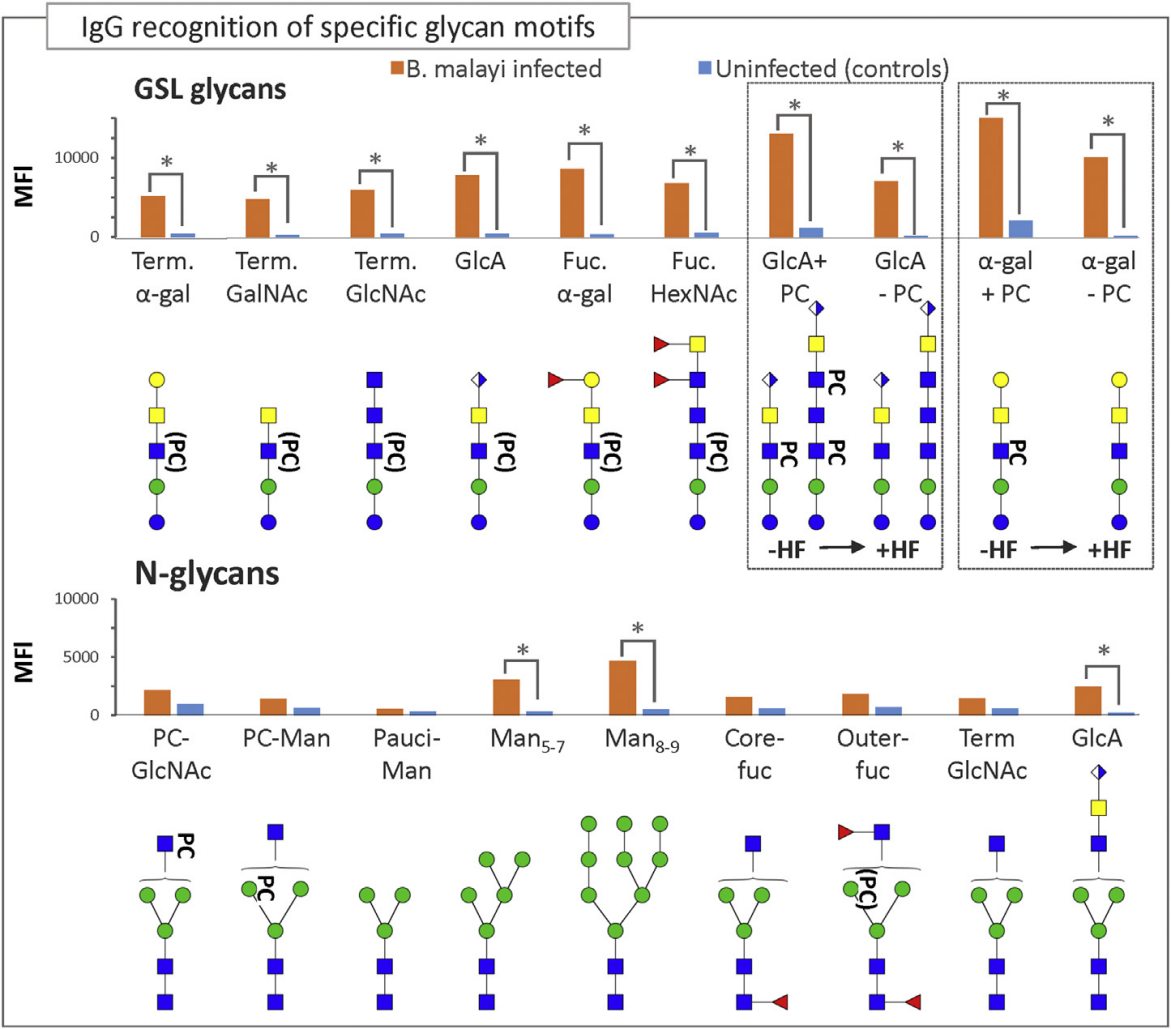
**Plasma IgG responses to the different glycan categories of *Brugia malayi*-infected and uninfected individuals.** Averaged MFIs to specific glycan motifs, based on glycan content of the fractions, were calculated for *B. malayi*-infected (Set 1) and uninfected individuals. Fraction groups are shown on the *x*-axis and named after the corresponding glycan motif present in the fractions: core-fuc, α1–6 core fucosylated *N*-glycan; Fuc. α-gal, fucosylated (terminal) α-1–4 Gal; Fuc-HexNAc, fucosylated HexNAc; Man x–y, Man_x–y_ (high-mannosidic *N*-glycans carrying 5–7 or 8–9 mannose residues); outer-fuc, α1–3 fucose attached to terminal GlcNAc; pauciman, paucimannosidic *N*-glycans; PC-GlcNAc, PC-substituted GlcNAc; PC-Man, PC-substituted mannose; Term. α-Gal, terminal α1–4 Gal; Term. GalNAc, terminal GalNAc; Term. GlcA, terminal GlcA; Term. GlcNAc, terminal GlcNAc. A representative glycan structure of each category is shown below the *x*-axis to illustrate the glycan feature in question. *Gray insets* on the *right* compare averaged MFIs of selected GSL fractions with and without HF treatment, that is, with PC substituents removed as illustrated below the *x*-axis, where structures contained in the fractions are represented. Significant differences between groups were assessed using Bayesian statistics. *p* Values <0.05 that indicate a significant difference in MFI values between groups of individuals are represented using gray asterisks and connecting lines. HF, hydrofluoric acid; Ig, immunoglobulin; MFI, median fluorescence intensity; PC, phosphorylcholine.

Next, we screened our microarrays with a second set of plasmas from *B. malayi*-infected patients who were treated with the anthelminthic DEC (set 2, see Experimental Procedures section). Interestingly, the IgG responses from *B. malayi*-infected individuals dropped markedly following treatment (Fig. 7*B*). Paired sample analysis (a moderated paired *t* test allowing for sib-pair effects in the linear model) confirmed a significant drop (*p* < 0.05) for IgG binding to 23 printed *N*-glycan samples and 22 printed GSL glycan samples after DEC treatment. Moreover, most of the fractions that were no longer recognized post-treatment were the same as those that showed significant differences between infected and healthy individuals (Fig. 7, *A* and *B*, supplemental Fig. S6, and supplemental Table S8). We noted that similar to our findings for infected rhesus macaques, higher IgG levels were observed for GSL glycans than *N*-glycans. GSL glycan-containing fractions indeed yielded very high MFI levels in both sets of *B. malayi*-infection plasmas (Fig. 7, *A* and *B*). We performed the same analysis as for the rhesus macaque cohort, grouping the glycan fractions by categories in order to investigate whether antiglycan IgGs in human infection plasma are directed toward specific glycan structures. We calculated statistical differences between the *B. malayi*-infected individuals (set 1) and uninfected individuals for each category of glycan (Fig. 8). Infected individuals showed specific IgG binding to all *B. malayi* GSL glycan categories when compared with uninfected donors. Similarly, binding to *N*-glycans was significantly higher to high-mannosidic (both Man_5–7_ and Man_8–9_ categories) and GlcA containing structures, whereas MFI values to the other *N*-glycan categories were relatively low for both groups and did not show any statistically significant differences. These results were similar to the antiglycan IgG responses observed for rhesus macaques when comparing preinfection and postinfection time points. HF treatment of selected fractions lead to a clear decrease in MFI values for *B. malayi*-infected individuals although not statistically significant. Moreover, IgG binding to the fractions after removal of PC was still significantly higher for infected than uninfected individuals for GlcA-containing fractions as well as for the α-Gal-containing one. This suggests that, in *B. malayi*-infected individuals, an appreciable proportion of IgGs are directed toward the GlcA and α-Gal epitopes from the GSL glycans.

## Discussion

While known to play a crucial role in many features of parasitism, particularly in modulation of the host immune responses (27, 62, 63) and as targets of the antibody response (28, 59, 64), knowledge of parasitic filarial nematode glycans is still poor. Here, we conducted the first comprehensive characterization of *B. malayi N*-linked and GSL glycans using a MS-based approach including orthogonal glycan sequencing techniques, in combination with glycan microarray assessment of their antigenicity. We observed various types of *N*-glycans, the most abundant being paucimannosidic and high-mannosidic structures. Core fucosylated glycans were also found, as well as structures with up to five GlcNAc residues in addition to the two of the chitobiose core. Up to three of these GlcNAc residues are directly attached to the trimannosyl core possibly extended by additional β(1–4/6)-linked GlcNAc elements resulting in *N*-glycan structures with a maximum of three antennae, as described for *C. elegans* (65). In addition, we did not observe in *B. malayi N*-glycans containing up to 15 GlcNAc residues detected in both *A. viteae* and *Onchocerca volvulus* (19, 31). We also did not detect α1–3-linked core fucose known to be present for instance in *C. elegans* and *Haemonchus contortus N*-glycans (65, 66). However, we did observe a subset of *N*-glycans containing PC-substituted GlcNAc residues, which have been reported previously in filarial nematodes (67), including on the *A. viteae* excretory glycoprotein ES-62 (68), and which are known to be associated with a plethora of immunomodulatory properties (29). Surprisingly, we also found PC as substitution on the α1–3-linked mannose of the pentasaccharide core of some of the *N*-glycans. Previously, PC had only been described as a GlcNAc substituent in parasitic nematodes (30, 63, 67).

In the GSL glycans, PC substitutions of GlcNAc residues were detected as well. Notably, the major structure in *B. malayi* GSL glycans is a PC-containing pentasaccharide (*m/z* 1195.43 [M − H]^−^), which, in terms of monosaccharide composition, is also known to be expressed by *O. volvulus*. This zwitterionic glycolipid is thought to be a highly conserved antigenic structure through the nematode phylum, and it was elucidated to be Gal(α1–3)GalNAc(β1–4)[PC-6]GlcNAc(β1–3)Man(β1–4)Glc(1–1)ceramide for *O. volvulus* (20). In *B. malayi*, however, our exoglycosidase digestion data indicate that the terminal Gal is present in an α1–4 linkage to GalNAc rather than α1–3. Another interesting trait of *B. malayi* glycans is the presence of structures containing a GlcA sugar, which is particularly abundant as a terminal motif of GSL glycans (Figs. 2 and 5) but to a lesser extent also detected in *N*-glycans (Fig. 4). GlcA was recently reported in the terminal position of the *N*-glycans of *Dirofilaria immitis* (the canine heartworm filarial nematode) (60) as an extension of LDN motifs. Our study suggests a similar context for this monosaccharide in *B. malayi*, based on sequential β-*N*-acetylglucosaminidase and β-*N*-acetylhexosaminidase digestions of the product of the β-glucuronidase treatment of GlcA-containing *N*-glycans. The presence of GlcA in parasitic nematode glycans is a relatively new finding, and our study is, to the best of our knowledge, the first report of its presence in parasitic nematode GSL glycans. It is also interesting to note that the free-living nematode *C. elegans* is known to synthesize neither glucuronated *N*-linked glycans nor GSL glycans (62). These findings raise the question of the potential biological role of this residue. GlcA-containing motifs are a major feature of insect glycans (17, 69, 70) and one of the most recurrent anionic modifications of invertebrate *N*-glycans where it is hypothesized to be a capping equivalent of the ubiquitous terminal sialic acid residues of mammalian *N*-glycans (71). In addition, GlcA residues are present in abundance in the proteoglycans of many species (72), including humans. Here, they are known to be involved in immune processes (73, 74), which might suggest a role of glucuronated glycans in the stimulation or modulation of immune interactions, although this is yet to be further investigated. Moreover, it has been reported that the mAb used in the rapid diagnostic test for *W. bancrofti* circulating filarial antigen binds to a carbohydrate-containing β-linked GlcA (35) attesting to the presence of this residue on excreted/secreted components of this filarial nematode and thus, supporting the idea of a role in host–parasite interaction. Finally, another characteristic observed in this study is the negligible stage and sex specificity of glycan expression that has been reported for some other helminth parasites such as schistosomes (36, 75). A few differences in terms of relative abundance were observed, for instance, L3s seem to express a higher amount of highly mannosylated *N*-glycans than the other stages (supplemental Fig. S1), but we did not observe any clear qualitative structural differences between the various life stages we examined. This was surprising considering that *B. malayi* proteomic studies demonstrated sex-specific and stage-specific protein expression (76, 77). It is also worth mentioning that we focused on the parasite stages present in the human host, and that more marked differences in *N*-linked and GSL glycans might be observed in the insect stages.

To assess the antigenicity of the observed *B. malayi* glycans during infection and pinpoint defined glycan targets of the antibody response, we characterized antiglycan antibodies in serum/plasma from *B. malayi*-infected rhesus macaques and humans by applying glycan microarray technology. It is well known that infection with helminths leads to strong antibody responses directed against the parasite's glycans (13, 58, 61, 64, 78), but for filarial nematodes, this was yet to be investigated. In infected rhesus macaques, IgM, and to a lesser extent IgG, was present at the preinfection time point. This has already been observed in other infectious diseases (61, 79) and might be explained by the fact that both IgM and IgG at baseline are most likely triggered by glycans from previous exposures to infectious agents, commensals, insect bites, or other environmental triggers, leading to recognition of a subset of cross-reactive glycans. Clearly, several of the identified and printed *B. malayi*-derived glycans and glycan motifs contained therein are not unique to the parasite but shared with other nematodes, invertebrates, plants, or mammals. IgM signals to such cross-reactive glycans may be more abundant because of the pentavalent nature of IgM, which increases its avidity in the glycan array assay (79, 80). Nonetheless, the preinfection levels were strikingly lower when compared with postinfection MFI levels, especially for GSL glycans, showing a very strong induction of IgG and IgM to those glycans upon infection, while binding to *N*-glycans remained relatively weak, particularly for IgM. This corroborates our structural findings since *B. malayi* arthrotype GSL glycans are not only different from mammalian GSLs in the core structure (belonging to the isoglobo, globo, and gangliosides series in mammals (81)), they are in addition extended by antigenic motifs, such as PC, GlcA, and (fucosylated) α-Gal. In contrast, *B. malayi N*-glycans present more similarities to mammalian ones as shown by our structural data and confirmed by array screening with the mAb 100-4G11 that is specific for the trimannosyl epitope present on Man3 and Man5. *N*-glycan structures to which most antibody responses were observed were high-mannosidic glycans, already known to be recognized by antiglycan antibodies in other parasitic (12, 61, 82) or viral (83) infections. In addition to the recognition of high-mannosidic *N*-glycans, IgG binding to fucosylated, GlcA-bearing, and PC-substituted *N*-glycans was also observed to a lesser extent. Comparison of fluorescence levels obtained for native GSL fractions and for the same fractions post-HF treatment (*i.e.*, with PC removed) clearly showed that a large part of IgG and IgM responses was primarily directed toward the backbone of the GSL structures and that PC substitution was not the main or sole motif responsible for Ig binding.

Our finding is similar to observations made for antibody binding from *W. bancrofti* infection sera, in which case binding to a mixture of parasite carbohydrate was maintained even after sera were absorbed with PC–bovine serum albumin (28). In our study, this was particularly clear for the GlcA-containing structures. Although binding to the α-Gal-containing structure was also detected, it appeared to be less specific for infection; there were no statistical differences for IgG binding, and differences in IgM binding were only observed for the PC-containing structure. This observation might be explained by pre-existing antiglycan antibodies in rhesus macaque sera recognizing the terminal α-Gal motif before *B. malayi* infection. Antibodies to α1–3 Gal are abundantly present in human, apes, and Old World monkeys (84). These species have evolved with inactivation of the α-1,3-galactosyltransferase gene, but they are constantly exposed to terminal α1–3 Gal motifs from food, the microbiome, and possibly pathogens, to which high antibody titers are raised (85). It is highly likely that these antibodies crossreact with the *B. malayi* α1–4 Gal epitope despite the linkage difference, or that α1–4 Gal-directed antibodies are also present in uninfected individuals and animals because of previous crossreactive exposures. Our findings in terms of antiglycan antibody dynamics are similar to observation made during infection with *Schistosoma japonicum* in rhesus macaques (61) where IgM binding to the parasite glycans also appeared very early in the course of infection and lowered progressively, in combination with strong IgG recognition appearing later, coinciding with egg production. In our study, increase of IgG was observed from 5 to 15 wpi with a broader range of *B. malayi* glycans being recognized as well as a higher titer of IgG to many glycan structures, as indicated by increase in MFI values for fractions already significantly recognized at previous time points. This progressive IgG induction correlated with increasing blood microfilaremia (supplemental Table S2*B*). The rhesus macaque model studied here offers valuable insight into the dynamics of antiglycan IgG and IgM responses during establishment and development of LF infection in humans given the similarities to humans in terms of lymphatic pathology and immune responses during the development of Brugian filariasis (86, 87) and the difficulty of gathering such information from human subjects.

When screening our microarrays with plasma from *B. malayi*-infected human individuals, we only observed weak IgM signals, with no significant difference to control plasma from uninfected individuals. Possibly, antiglycan IgM levels are very low during chronic infection, which would match our observation that IgM during the course of infection in macaques is mainly observed at early time points. Aspecificity of IgM binding to *B. malayi* antigens has also been reported previously (48), with significant IgM found in nonendemic sera that was attributed to nonspecific PC reactivity. IgM binding to filarial carbohydrates has also been detected for *W. bancrofti*-infected individuals (28); however, without direct comparison to nonendemic controls. Binding of IgM to the surface of L3 larvae has been reported in *B. malayi* (88) although it is unclear whether this occurs *via* proteins or carbohydrates. Thus, if our study suggests a limited role of IgM in the response to *B. malayi N*-linked and GSL glycans during chronic infection, further work is needed to confirm this finding, and the possibility of an IgM response directed toward other glycan classes such as the O-linked glycans is not excluded. In contrast, strong IgG binding to *B. malayi* glycans was observed for plasma from infected individuals compared with plasma from healthy donors.

We found that antiglycan IgG profiles after anthelminthic treatment highly resembled uninfected donor profiles and suggests that antiglycan IgG could potentially be associated with current or recent infection status. More precisely, plasma was obtained from infected patients who were treated after the first blood sampling in 1990. The second sampling, after DEC treatment, took place almost 2 years later, after confirming amicrofilaremia of each donor. Thus, decrease of antiglycan IgG in plasma after chemotherapy occurred within this time frame, suggesting that antiglycan IgG response is a potential valid tool to discriminate between current/recent infections and past infections, which is particularly useful in low endemic areas and elimination settings. For that reason, it would be of great interest to elucidate the exact timing of plasma IgG decline by studying additional post-treatment time points. Another interesting path to explore further is the implication of the various IgG subclasses in glycan recognition. Previous studies have shown compelling evidence for specific Ig response to *B. malayi* adult somatic extract antigen (BmA) and IgG subclass associations within the subpopulation of *B. malayi*-infected individuals (*i.e.*, asymptomatic microfilariemic carriers, amicrofilariemic individuals,—or so-called “endemic normal”—and elephantiasis/symptomatic patients) (48, 89, 90, 91, 92, 93) as well as the impact of DEC chemotherapy on the BmA-specific Ig titers (49). In this study, a differential decline of IgG subclasses to BmA was observed after treatment, thus, it would be of particular interest to examine how IgG subclasses respond to glycan antigens and to study the association of antiglycan antibodies with the various endemic subpopulations.

The four human IgG subclasses differ with respect to antigen binding, immune complex formation, complement activation, triggering of effector cells, half-life, and placental transport (94). IgG3 in particular is known for having the shortest half-life of all subclasses (95), which might be an asset to detect current infections only. In addition, studies have shown that different levels of sensitivity and specificity were reached when using different IgG subclasses to detect nematode infections (96, 97). Consequently, addressing these questions might be critical for evaluation of the diagnostic potential of host antiglycan IgGs and their use as markers of active infection with *B. malayi*.

Finally, we are aware that our study relies on a limited set of samples (n = 5 individuals for each group), which might contribute to the difficulty in drawing unequivocal conclusions because of the large individual-to-individual variation (supplemental Fig. S6 and supplemental Table S8). Many factors such as the different duration of infection in each individual and variability in IgG titers in the subpopulation types for *B. malayi* infection may contribute to the observed fluctuations. However, we were encouraged that both sets of *B. malayi*-infected donors, obtained from separate studies, yielded very similar results in terms of IgG binding to the parasite glycans. In fact, fluorescence profiles of both sets of infected individuals on the glycan microarray were almost identical (Fig. 7, *A* and *B*) showing the validity of our glycan microarray strategy for comparison of antiglycan antibody recognition from various plasma. It allowed us to assert the antigenicity of the structural features identified during our structural characterization of *B. malayi* glycans by showing a major Ig response from infected hosts. An important aspect to investigate further is the recognition of *B. malayi* glycans by plasma/serum antibodies from individuals infected with other filarial nematodes as some features are shared between filarial nematode species (*i.e.*, major GSL structure, PC, and GlcA-containing glycan) (22, 60, 64). These parasite glycans need to be studied for crossreactivity with other related filarial nematode infection sera to explore specific and shared antiglycan antibody responses. In conclusion, our work paves the way to further study the role of *B. malayi* glycans during infection and suggests that the antiglycan antibody response from the host could be exploited for potential diagnostic markers to detect LF.

## Data Availability

MALDI-TOF–MS and MALDI-TOF–MS/MS spectra supporting our findings have been made available in the supplemental data. In addition, all raw MS data (MALDI-TOF–MS and MALDI-TOF–MS/MS) presented in this study (both from main and supplemental figures) have been deposited in Glycopost (https://glycopost.glycosmos.org/, project ID: GPST000240). Glycan array screening output files can be found in Synapse (https://doi.org/10.7303/syn26570309).

## Supplemental data

This article contains supplemental data (47, 48, 56, 57).

## Conflict of interest

L. M. C. P., C. H. T., and J. M. F. are employees of NEB. This funder is commercial but explicitly states that it played no role in the study design, data collection, and data analysis. All other authors declare no competing interests.
