# Supplementary material for: Mass Spectrometric and Glycan Microarray–Based Characterization of the Filarial Nematode Brugia malayi Glycome Reveals Anionic and Zwitterionic Glycan Antigens
Source: Mol Cell Proteomics. 2022 Jan 20;21(5):100201. doi: 10.1016/j.mcpro.2022.100201 (PMC9046957; doi:10.1016/j.mcpro.2022.100201)

Supplementary material

Mass spectrometric and glycan microarray-based characterization of the filarial nematode *Brugia malayi* glycome reveals anionic and zwitterionic glycan antigens

Laudine M. C. Petralia[1,2], Angela van Diepen[1], Lena A. Lokker[1], D. Linh Nguyen[1], Erliyani Sartono[1], Vishal Khatri[3], Ramaswamy Kalyanasundaram[3], Christopher H. Taron[2], Jeremy M. Foster[2], Cornelis H. Hokke[1]*

[1] Department of Parasitology, Leiden University – Center of Infectious Diseases, Leiden University Medical Center, Leiden, The Netherlands

[2] Division of Protein Expression & Modification, New England Biolabs, Ipswich, MA, USA

[3] Department of Biomedical Sciences, University of Illinois College of Medicine at Rockford, Rockford, IL, USA

* Corresponding author

List of supplementary material

	Page
**Figure S1: MALDI-TOF-MS spectra of PNGaseF and EGCase released N- and GSL glycans from various B. malayi life-stages**	S2 – S6
**Figure S2: Glycan sequencing of selected N-glycans**	S7 – S24
**Figure S3: Glycan sequencing of selected GSL glycans**	S25 – S40
**Figure S4: Glycan array screening with monoclonal antibodies for array validation**	S41 – S43
**Figure S5: Glycan array screening with longitudinal rhesus macaques infection sera**	S44 – S47
**Figure S6: Glycan array screening with human individual plasma**	S48-S52
**Table S1: List of glycosidases used for glycan sequencing**	.xls file
**Table S2: Information on *B. malayi* infected humans and rhesus macaques**	.xls file
**Table S3 : *B. malayi* N-glycans**	.xls file
**Table S4 : *B. malayi* GSL glycans**	.xls file
**Table S5 : Content of glycan fractions printed on microarrays**	.xls file
**Table S6 : Glycan array screening with monoclonal antibodies for array validation**	.xls file
**Table S7 : Glycan array screening with longitudinal rhesus macaque infection sera (Raw MFI data)**	.xls file
**Table S8 : Glycan array screening with human plasma (Raw MFI data)**	.xls file

Figure S1 – MALDI-TOF-MS spectra of PNGaseF and EGCase released N- and GSL glycans from various *B. malayi* life-stages

MALDI-TOF-MS measurements of released AA-labeled glycans were performed in negative-ion reflectron mode and all signals are labeled with monoisotopic masses. Known non-glycan signals are labeled with #. Ions corresponding to characterized glycan structures are labeled with a GP number: N-GP numbers for N-glycans (A), and GSL-GP numbers for GSLs glycans (D). GP numbers corresponding glycan structures can be found in Table S3 for N-glycans and Table S4 for GSL glycans. In order to evaluate whether the characterized glycans were reproducibly observed, we generated three biological replicates from separate batches of adult worms (mixed sex) and microfilariae (3 replicates per life-stage). The highly similar MALDI-TOF-MS spectra obtained are shown in B and C (N-glycans of adult worms and microfilariae, respectively) and in D and E (GSL glycans of adult worms and microfilariae, respectively). All spectra raw data can be found in Table S3 (N-glycans) and Table S4 (GSL glycans), in Tab B of both excel files.

A] *Brugia malayi* N-glycans

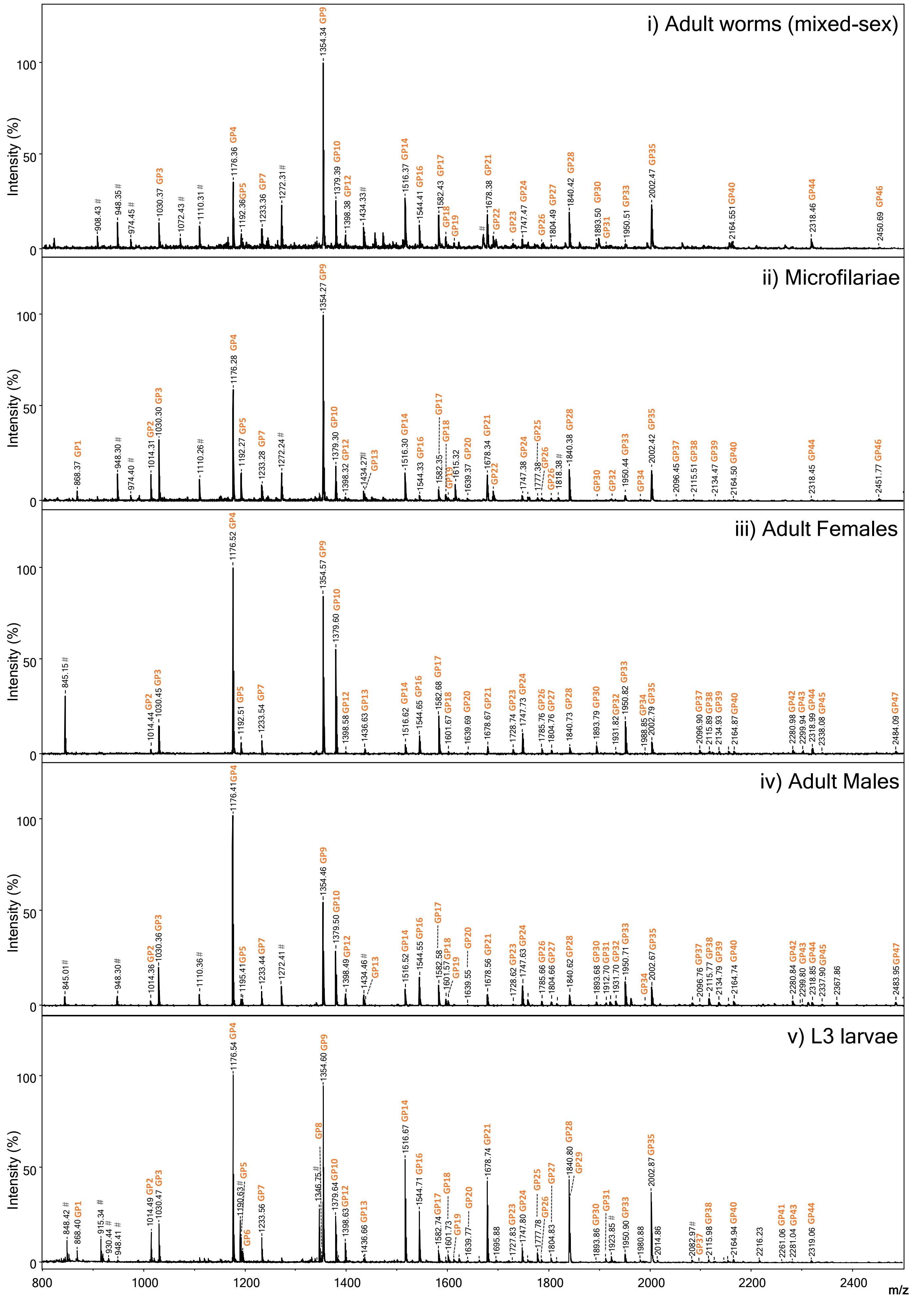

B] *Brugia malayi* adult worms N-glycans - biological replicates

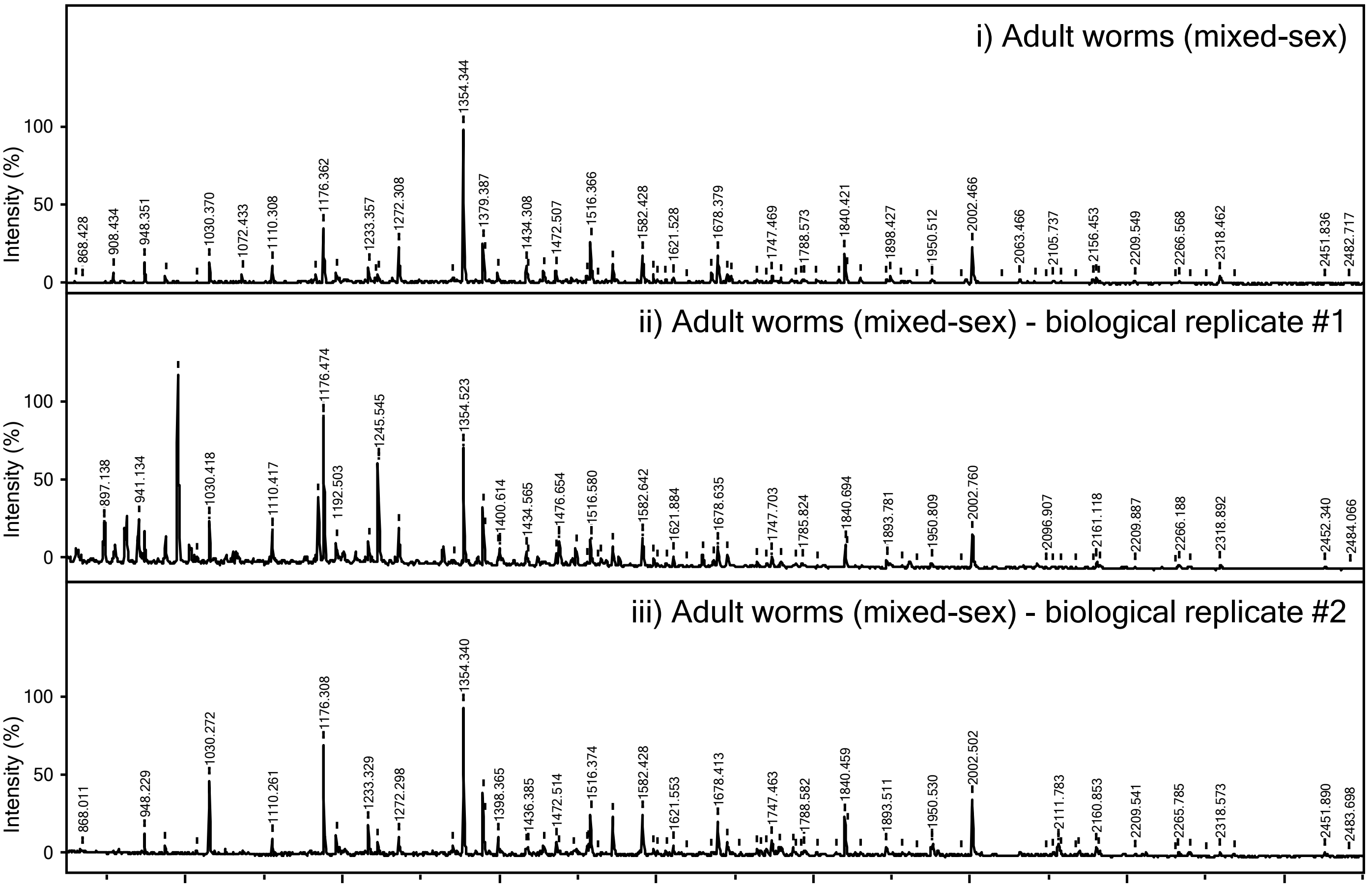

C] *Brugia malayi* microfilariae N-glycans - biological replicates

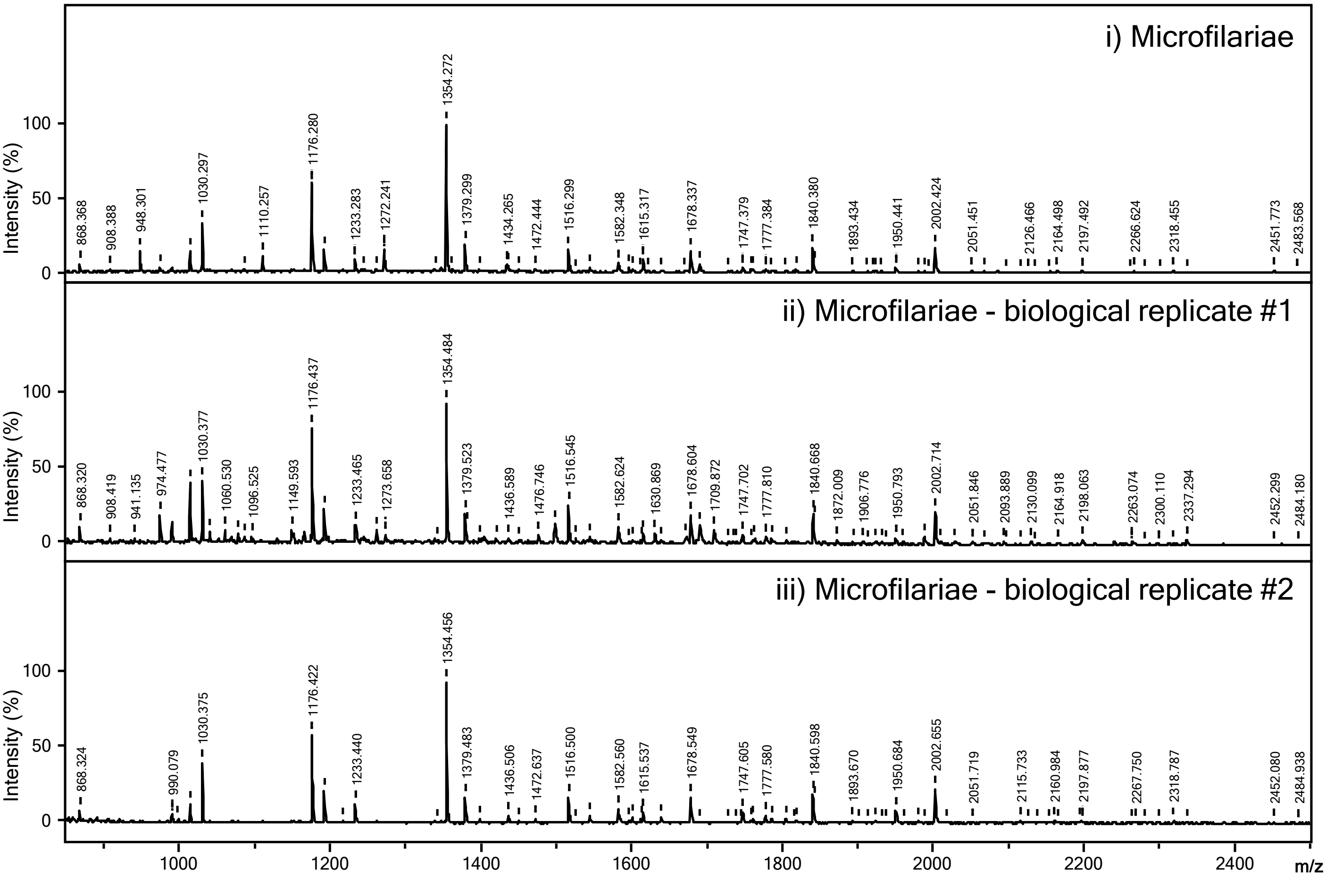

D] *Brugia malayi* GSL glycans

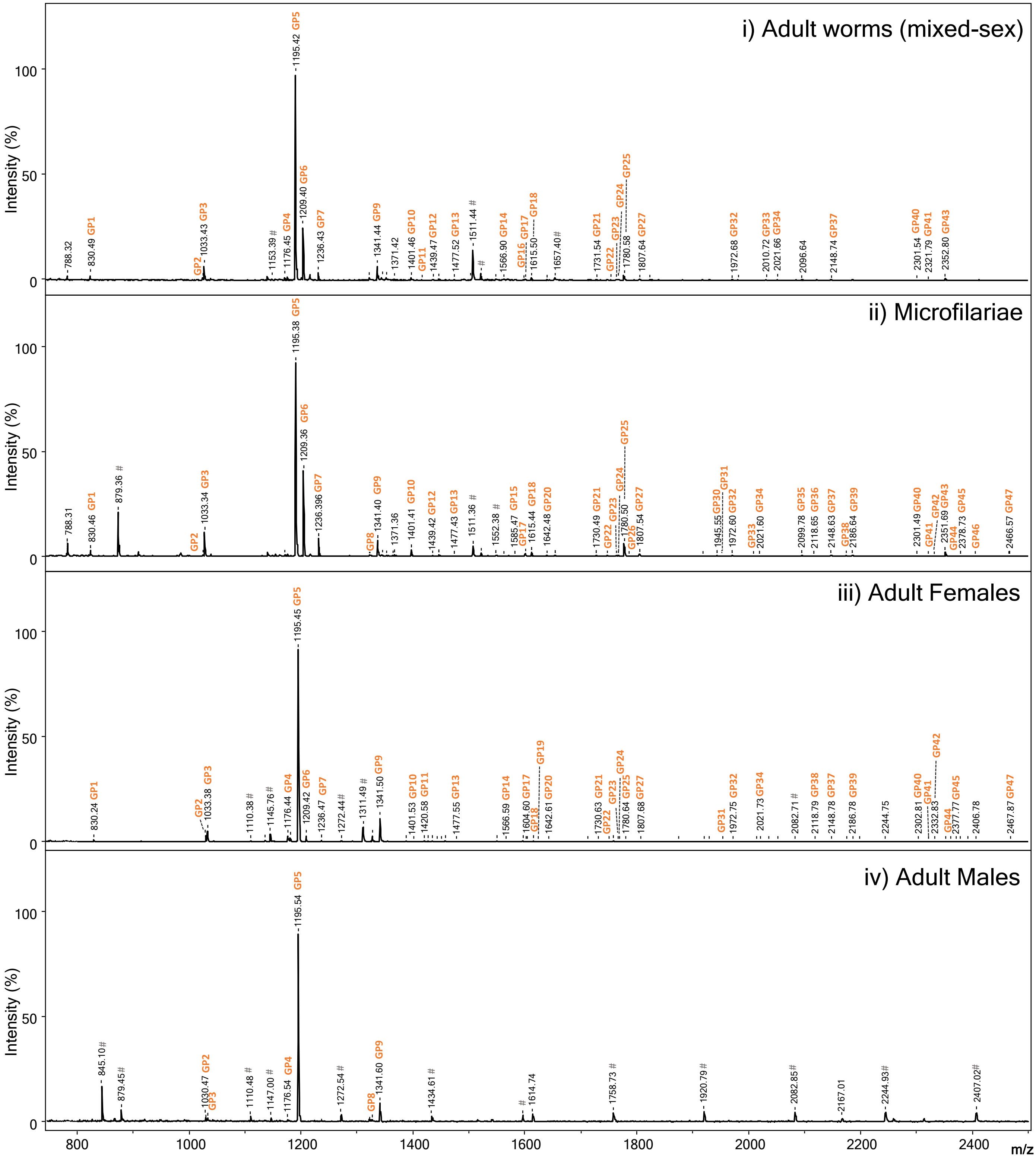

E] *Brugia malayi* adult worms GSL glycans – biological replicates

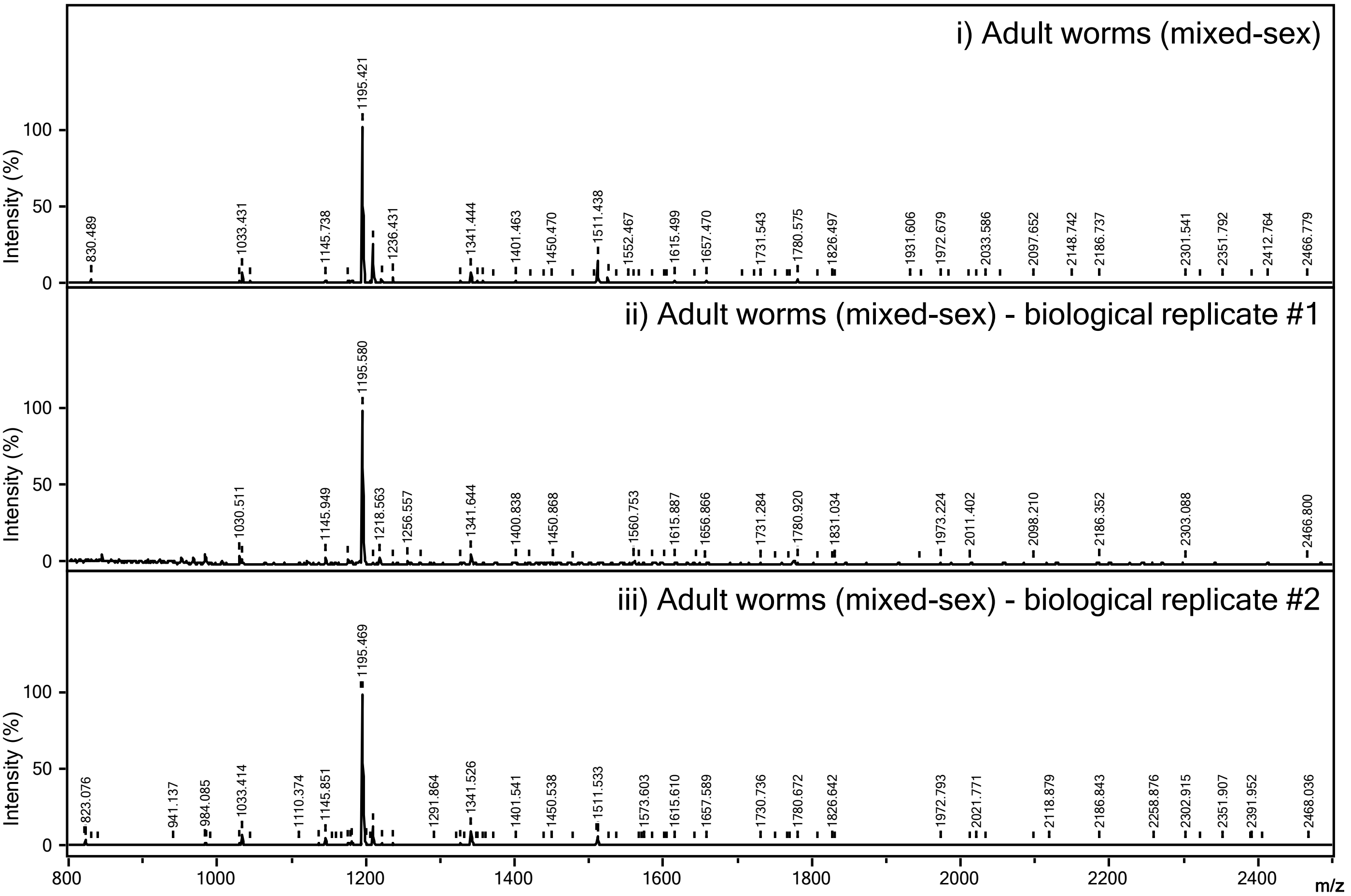

F] *Brugia malayi* microfilariae GSL glycans – biological replicates

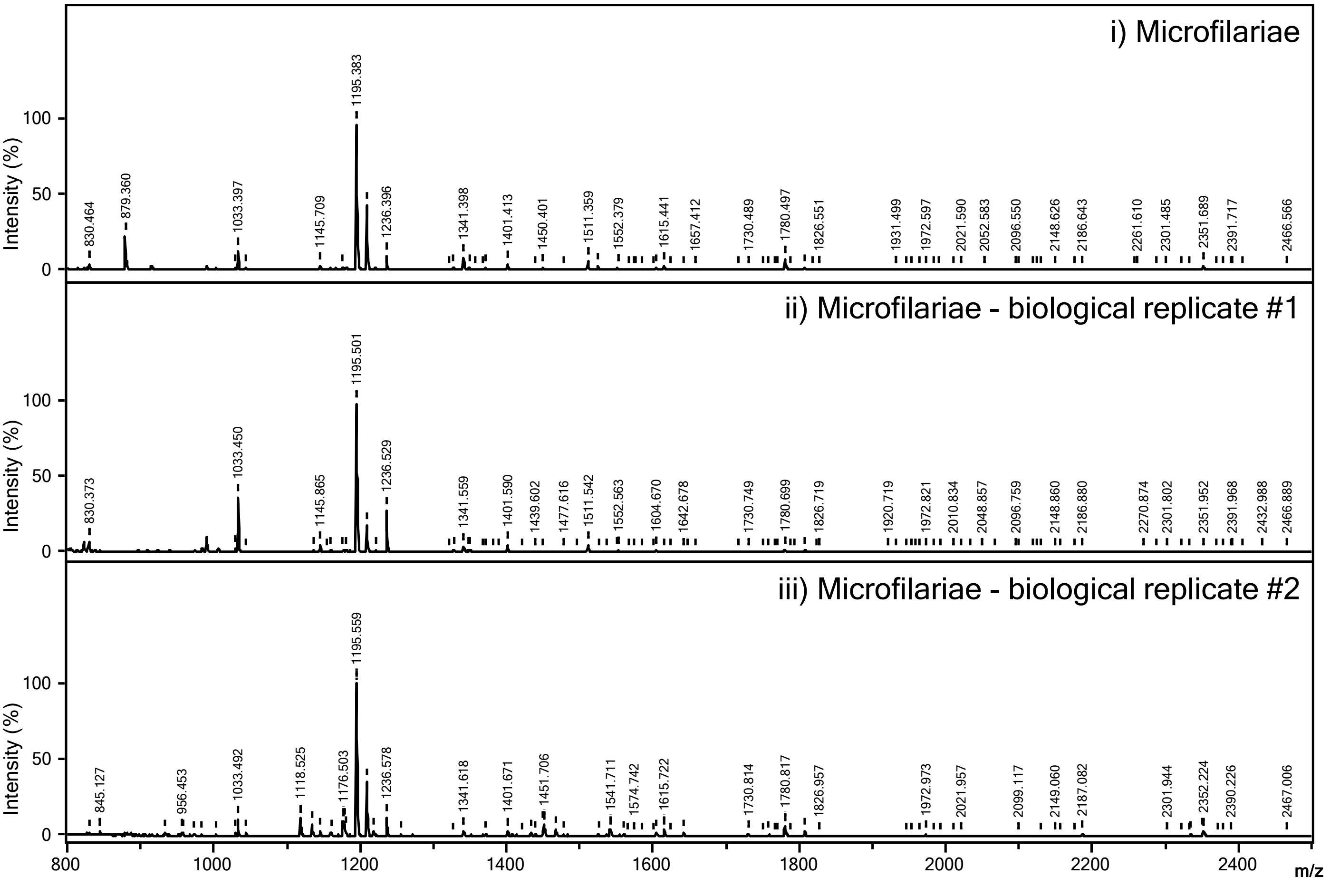

Figure S2 - Glycan sequencing of selected *B. malayi* N-glycans

MALDI-TOF-MS of exoglycosidase and HF treated *B. malayi* N-glycans (A-J) PNGase-F released and AA-labeled total (A-B) and UHPLC-purified (C-J) *B. malayi* N-glycans were subjected to various exoglycosidase digestions and/or incubation with hydrofluoric acid (HF). Protocols are detailed in the Experimental Procedures section and a list of the exoglycosidases used and corresponding reaction conditions is available in **Table S1**. Treatments performed on the N-glycans are indicated at the top of each panel and blue arrows highlight the products resulting from the aforementioned treatments.

MALDI-TOF-MS/MS of selected *B. malayi* N-glycan ions species (K-Q) Selected ions (m/z indicated in upper left corner) were subjected to fragmentation analysis. Resulting spectra are labeled with graphic representation of Y-type ions, unless indicated otherwise (B = B-type, C = C-type, Z = Z-type). Losses of a mass of 59 Da from the parent ion is indicative of loss of a PC (Refs #56, Timm, T. *et al.* and #57, Grabitzki, J. *et al.*) and is highlighted by a blue double arrow when necessary.

For all MALDI-TOF-MS and MS/MS spectra, measurements were acquired in negative-ion reflectron mode and signals are labeled with monoisotopic masses (m/z). AA-labeled glycans are represented using the CFG nomenclature: blue square = N-acetylglucosamine, green circle = mannose, PC = phosphorylcholine, red triangle = fucose, white and blue diamond = glucuronic acid and yellow square = N-acetylgalactosamine.

(A) HF treatment and sequential exoglycosidase digestions of adult female total N-glycan pool

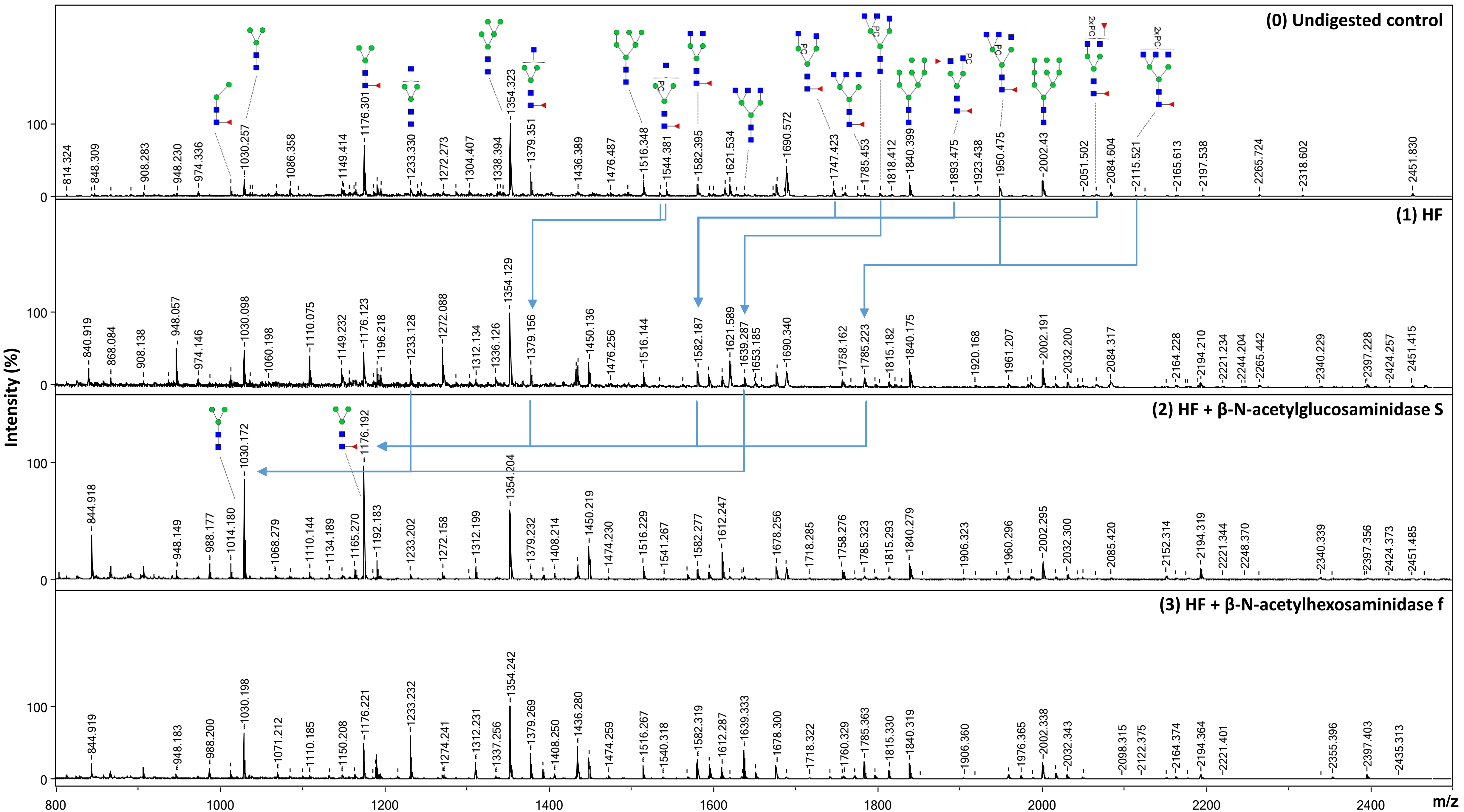

(B) Exoglycosidase digestions of adult female total N-glycan pool

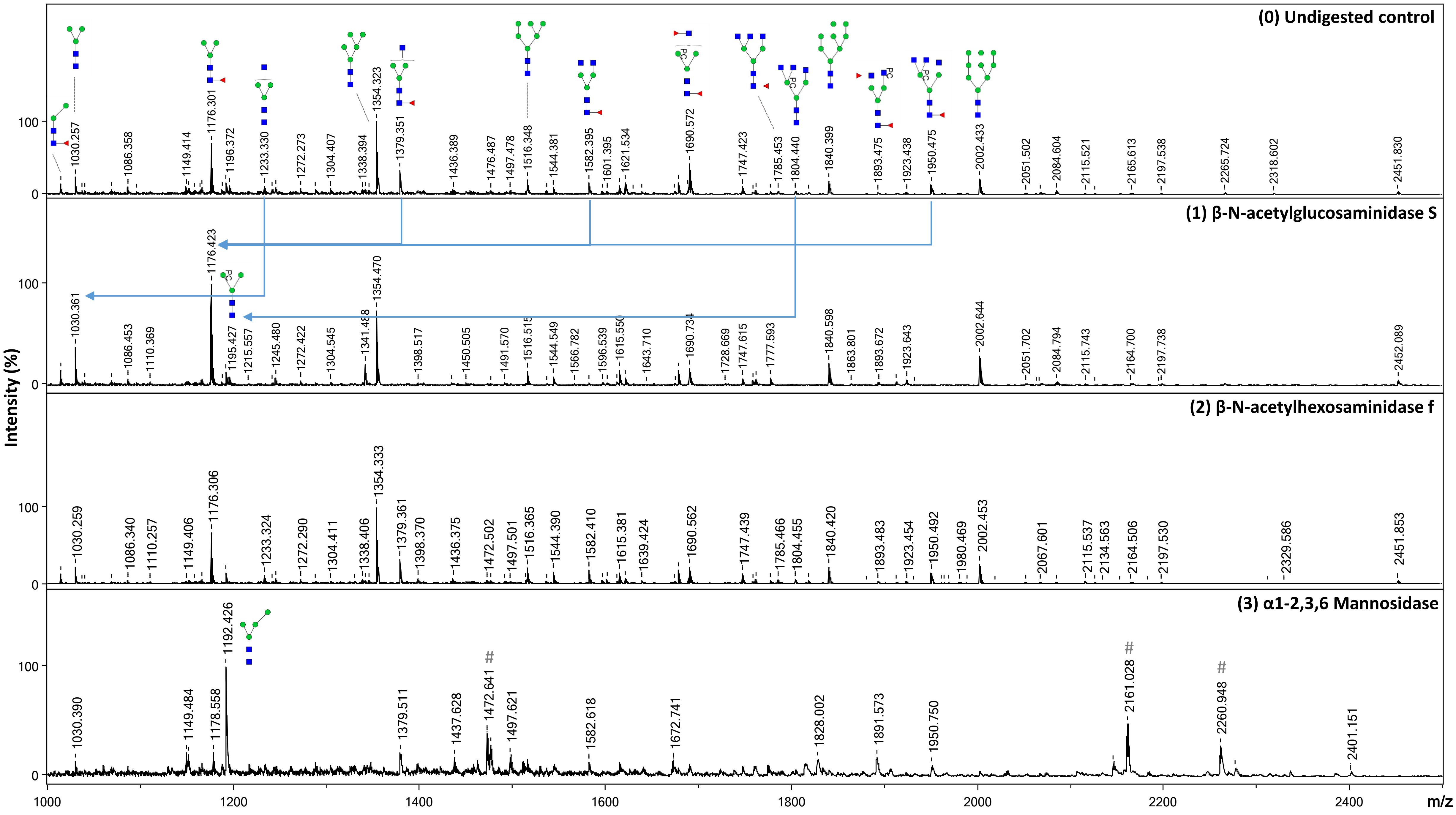

(C) Exoglycosidase digestions of purified fraction Ng10

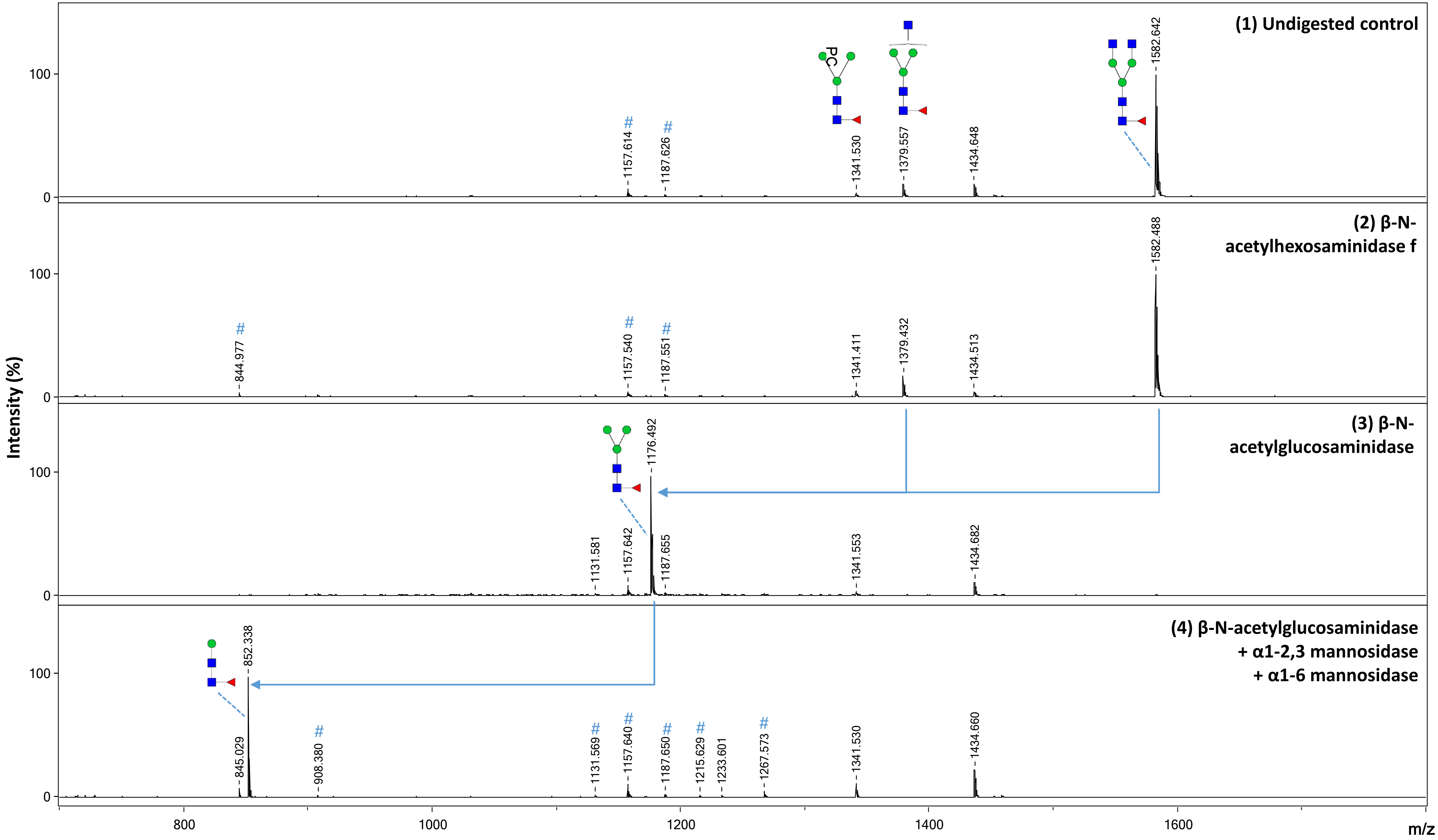

(D) Mannosidase digestion of purified fraction (not selected for array)

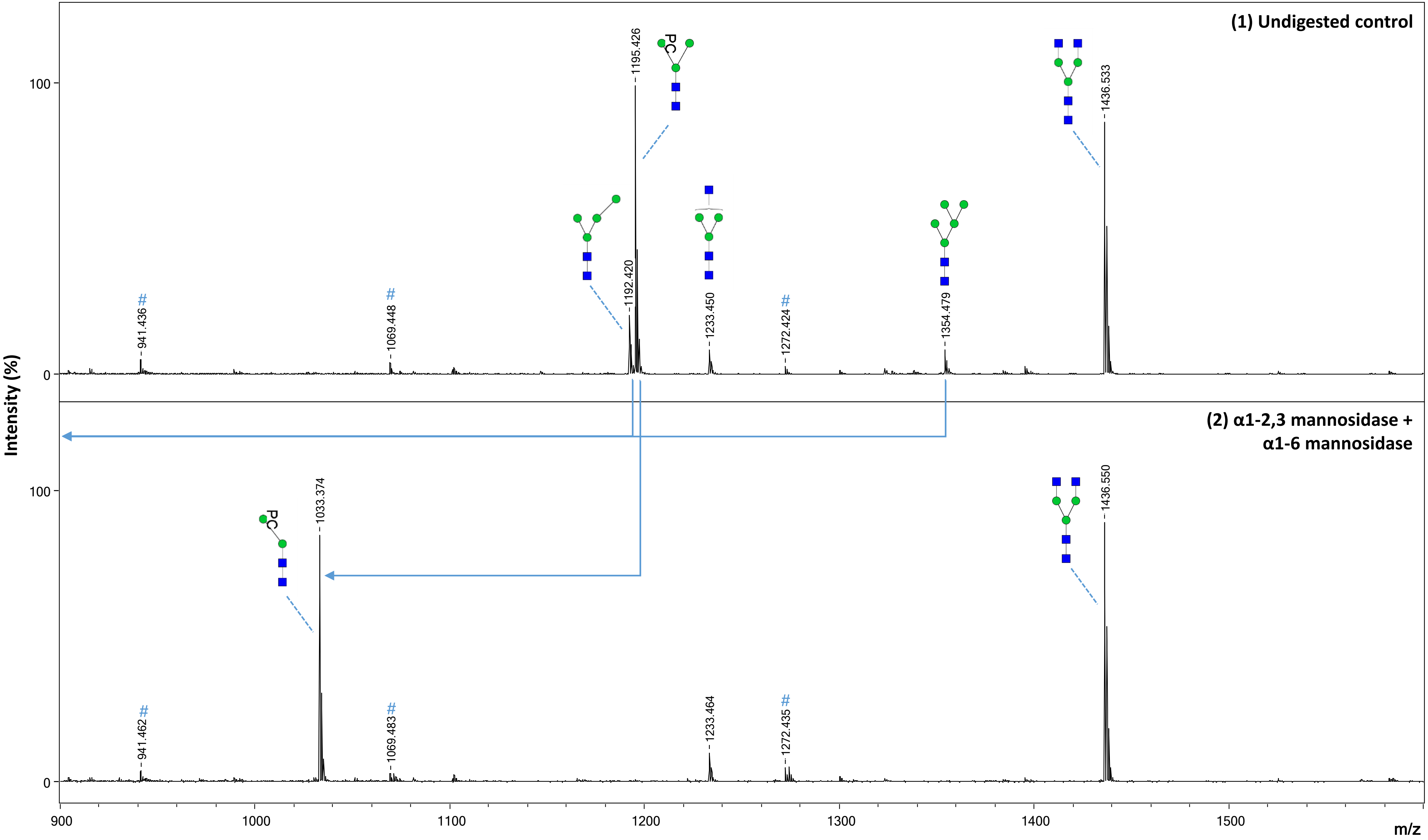

(E) Exoglycosidase digestions of purified fraction Ng30

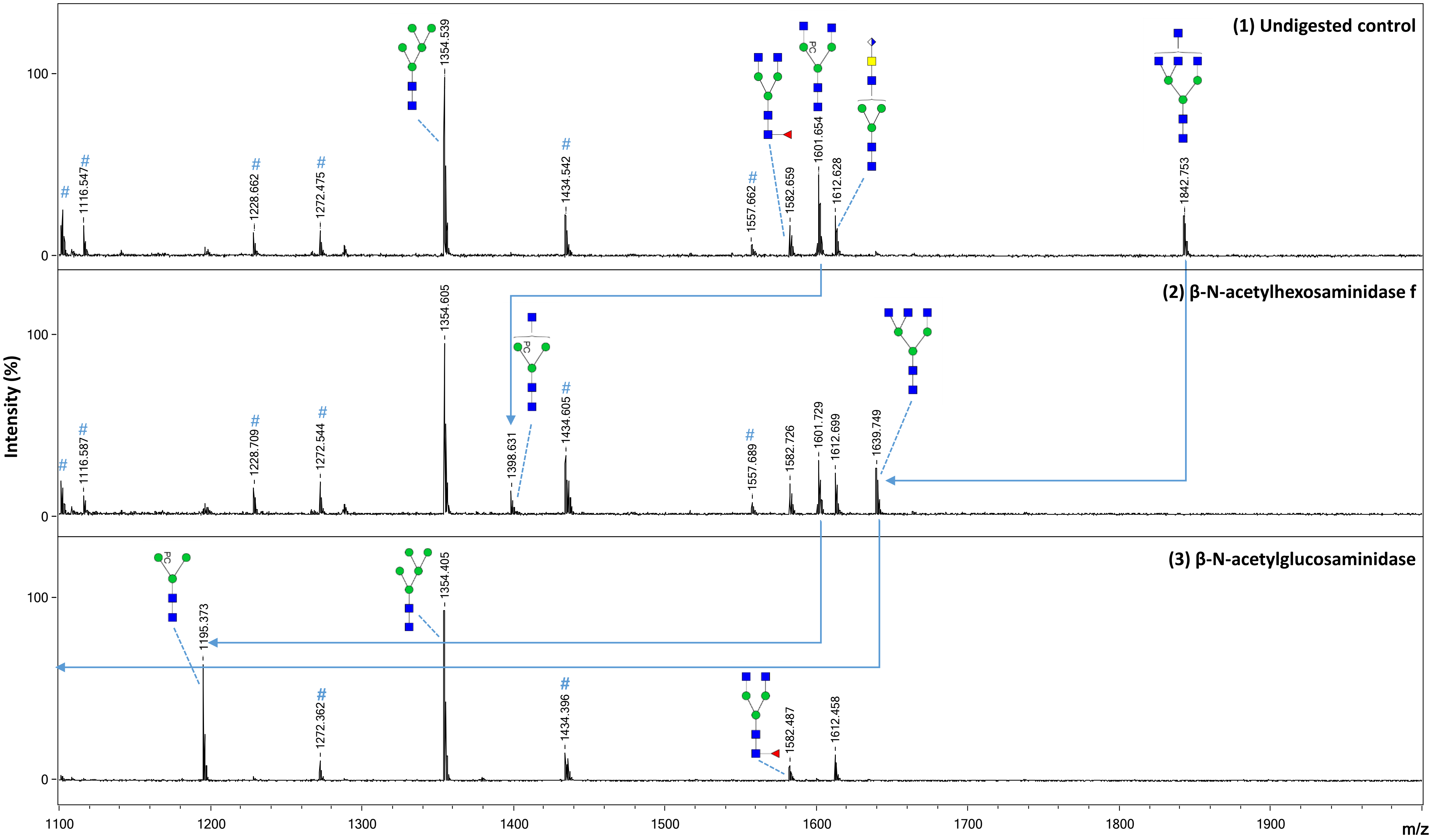

(F) Exoglycosidase digestions of purified fraction Ng7

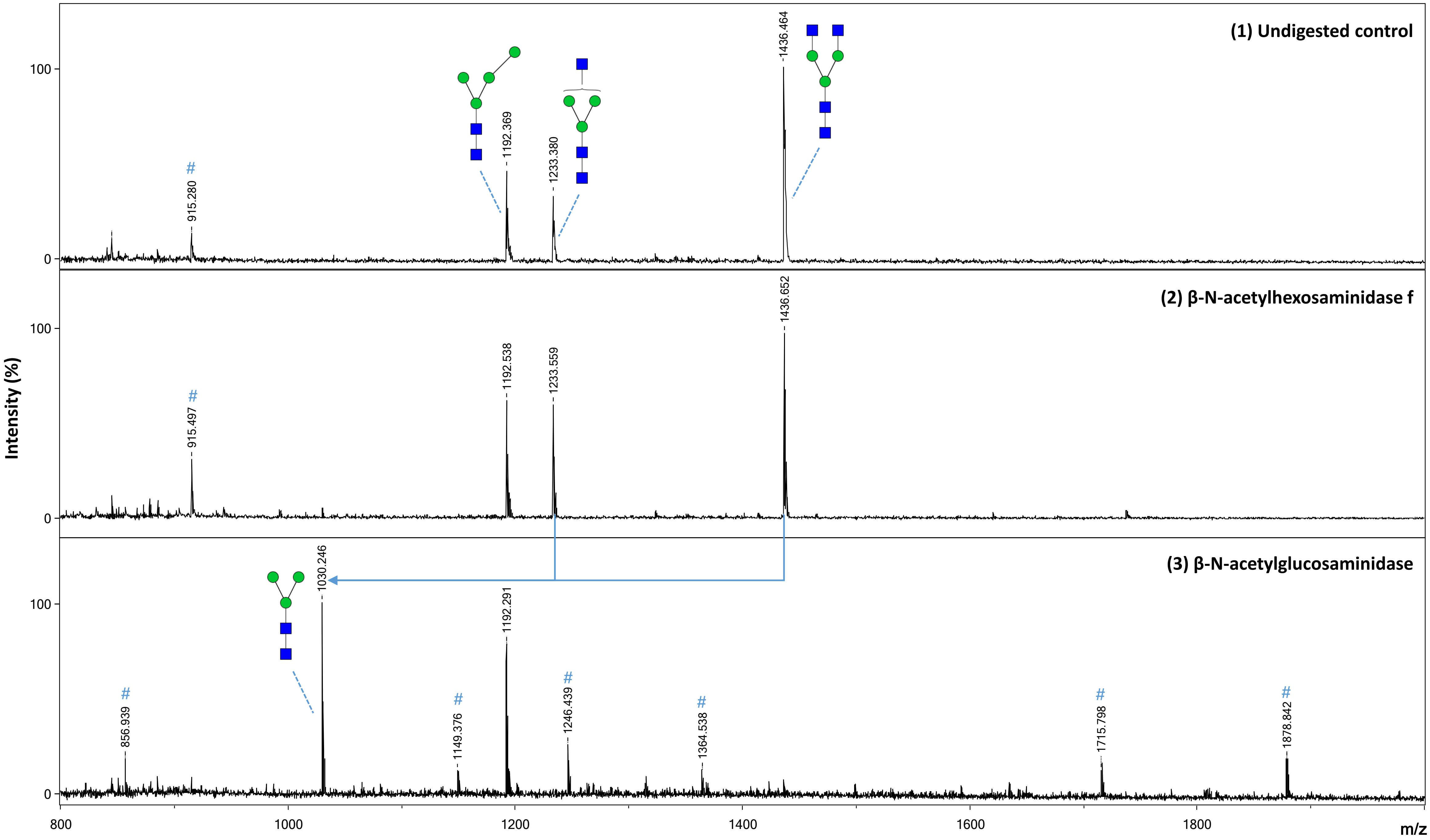

(G) Exoglycosidase digestions of a pool of purified fractions Ng12/Ng29/Ng33

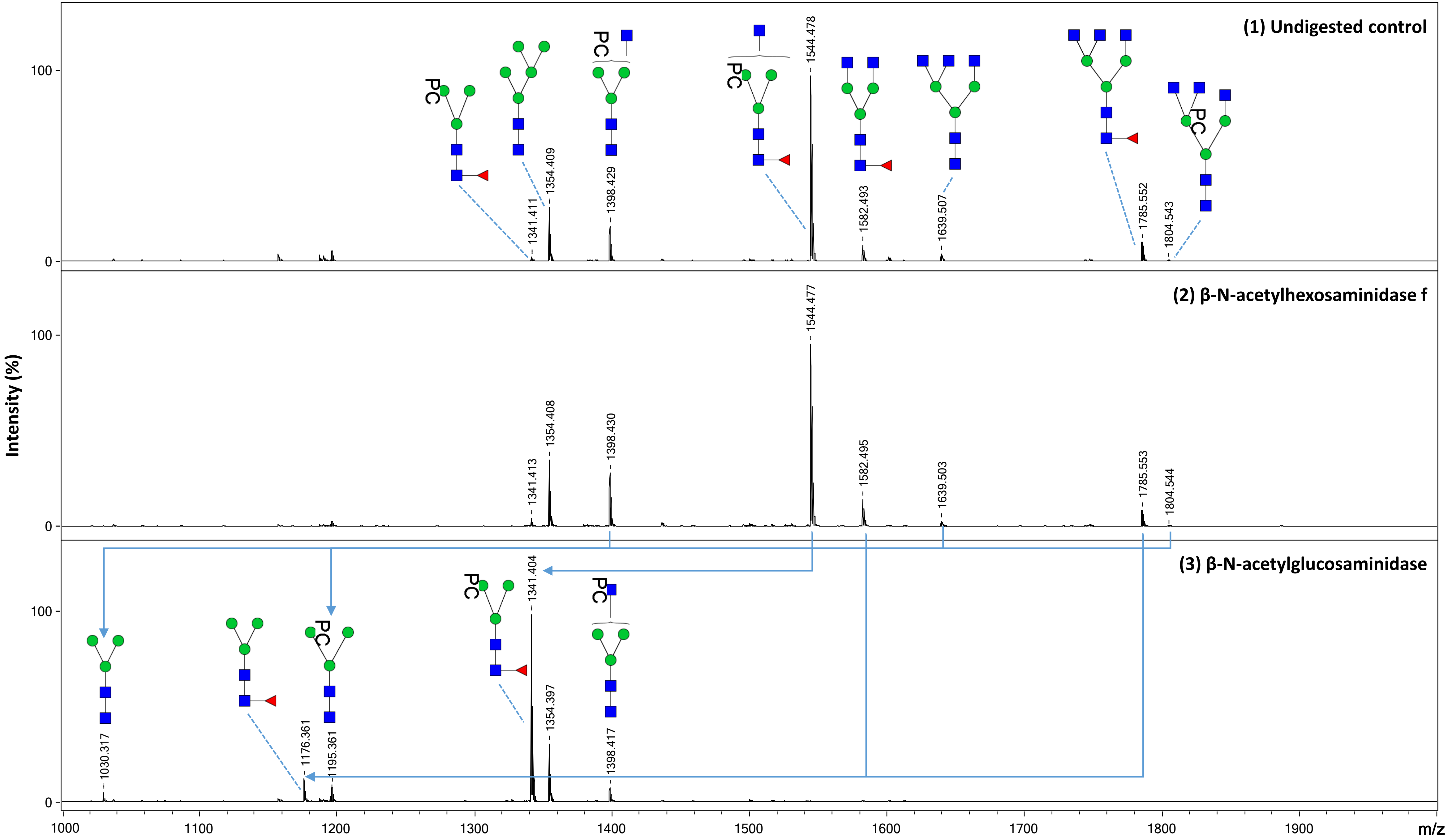

(H) β-N-acetylglucosaminidase digestion of purified fraction Ng24

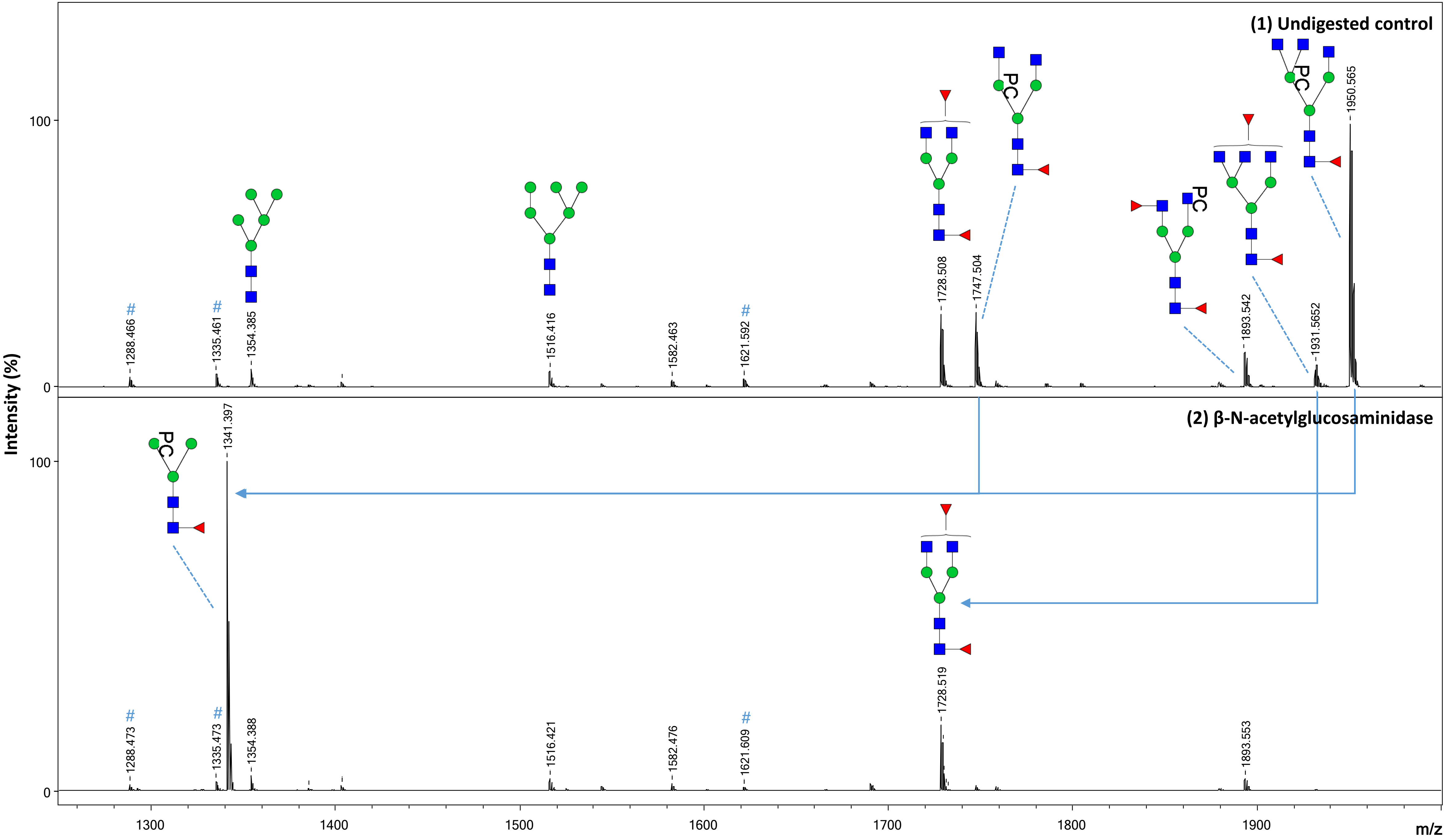

(I) HF treatment and sequential exoglycosidase digestions of purified fraction (not selected for array)

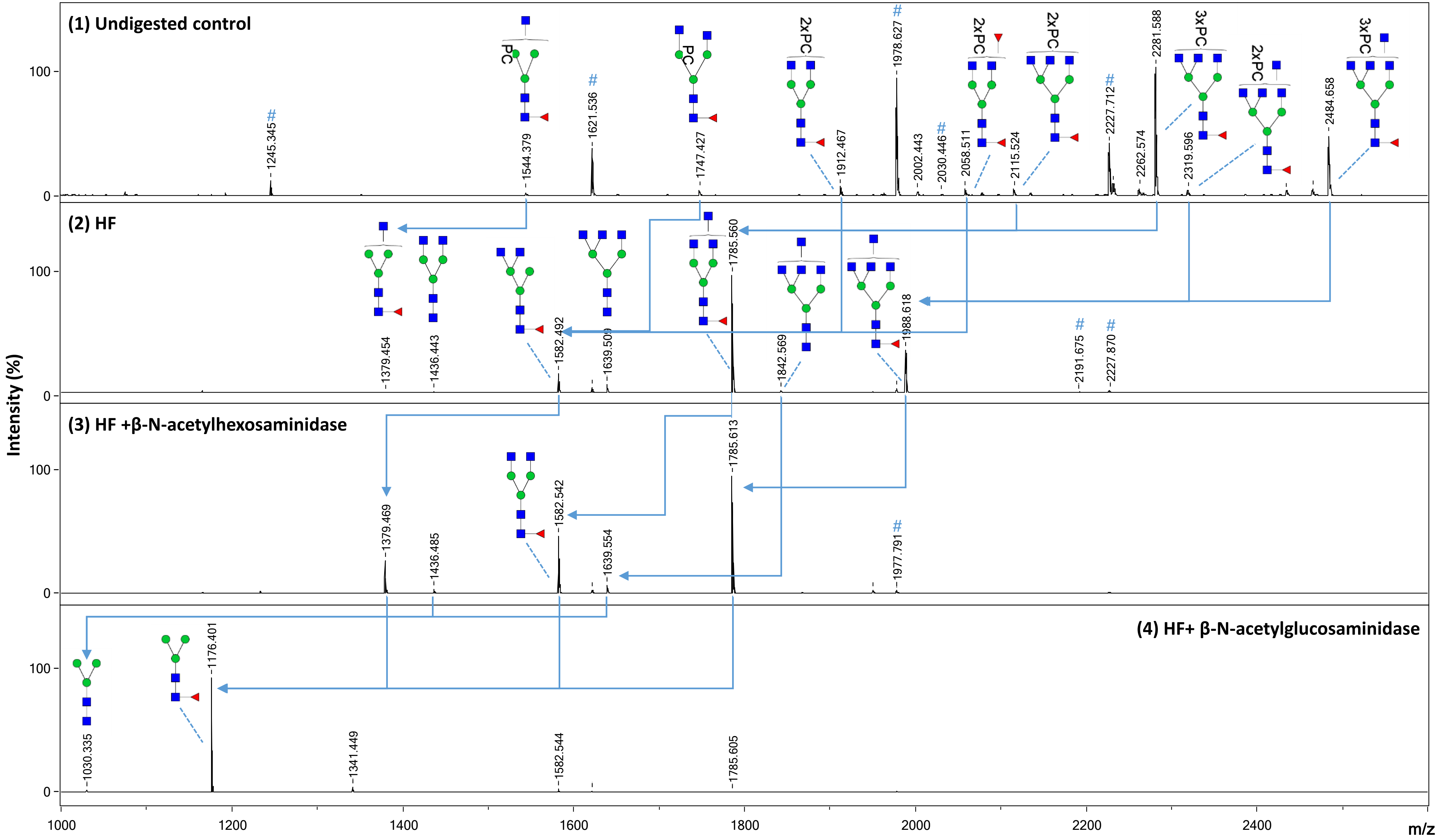

(J) Exoglycosidase digestions of purified fraction Ng32

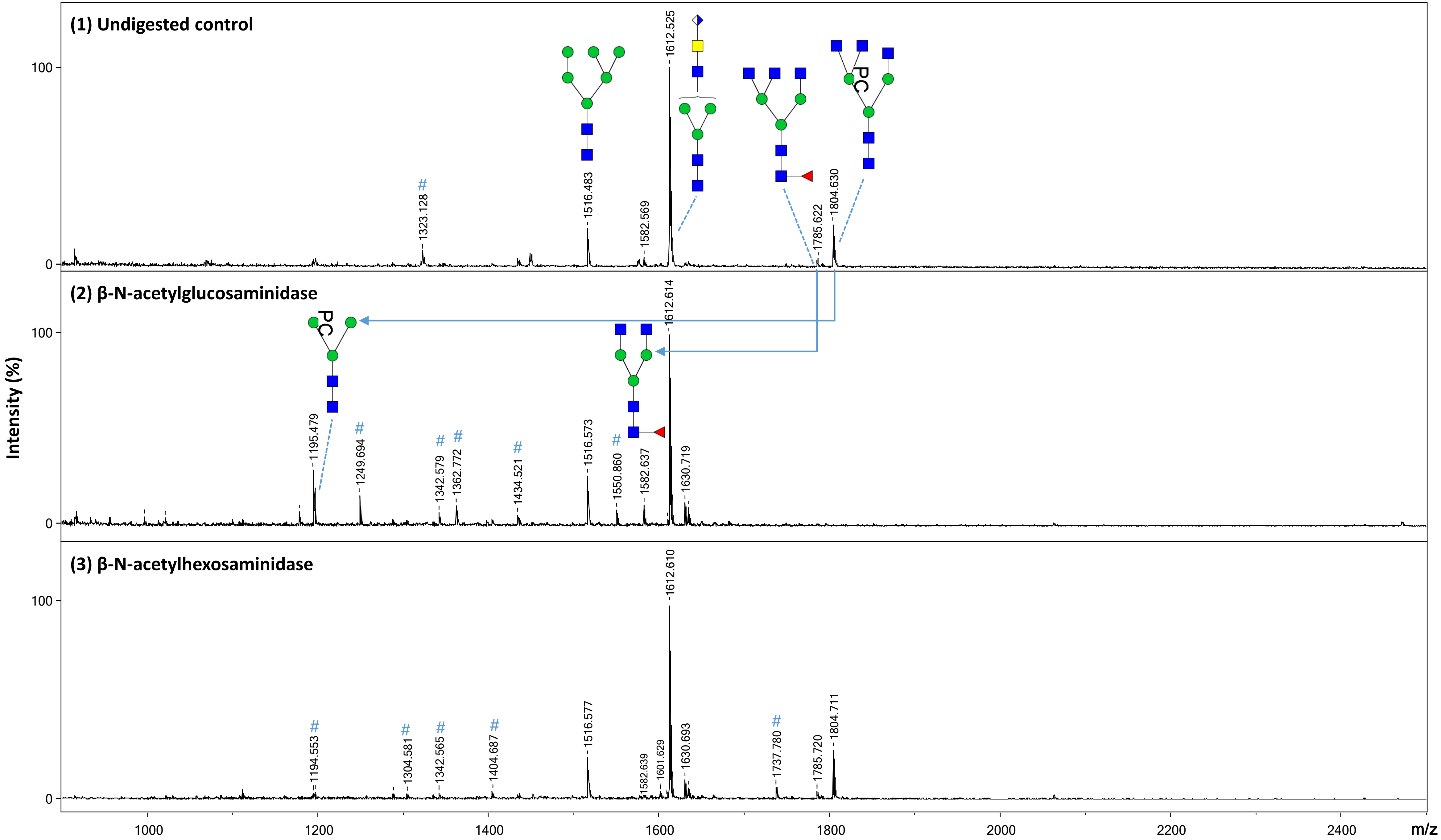

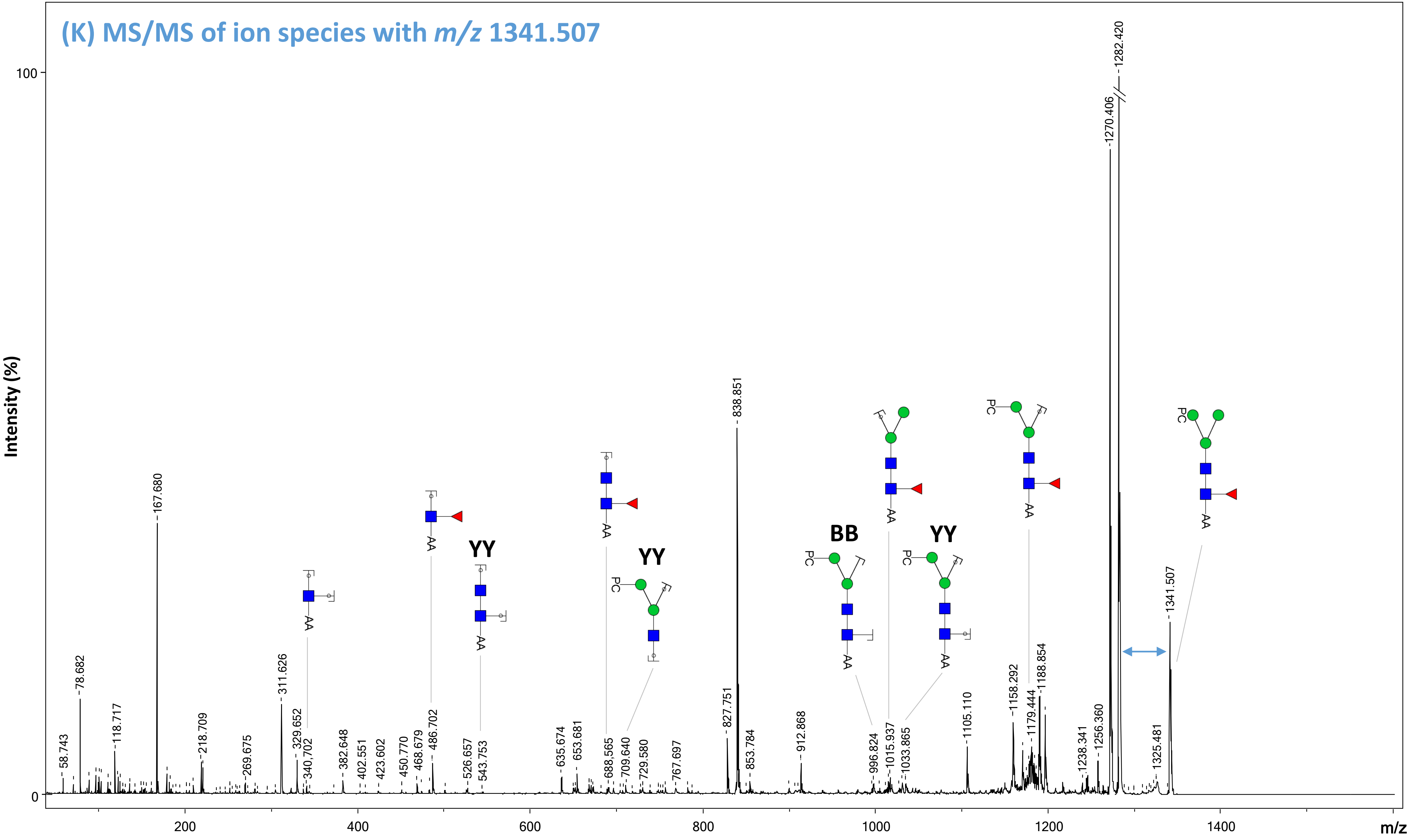
(K) MS/MS of ion species with m/z 1341.507
Intensity (%)
m/z
100
0
200
400
600
800
1000
1200
1400
1282.420
1270.406
838.851
167.680
78.682
118.717
58.743
218.709
269.675
311.626
329.652
340,702
382.648
402.551
423.602
450.770
468.679
486.702
526.657
543.753
635.674
653.681
688.565
709.640
729.580
767.697
827.751
853.784
912.868
996.824
1015.937
1033.865
1105.110
1158.292
1179.444
1188.854
1238.341
1256.360
1325.481
1341.507
YY
YY
BB
YY
AA
PC

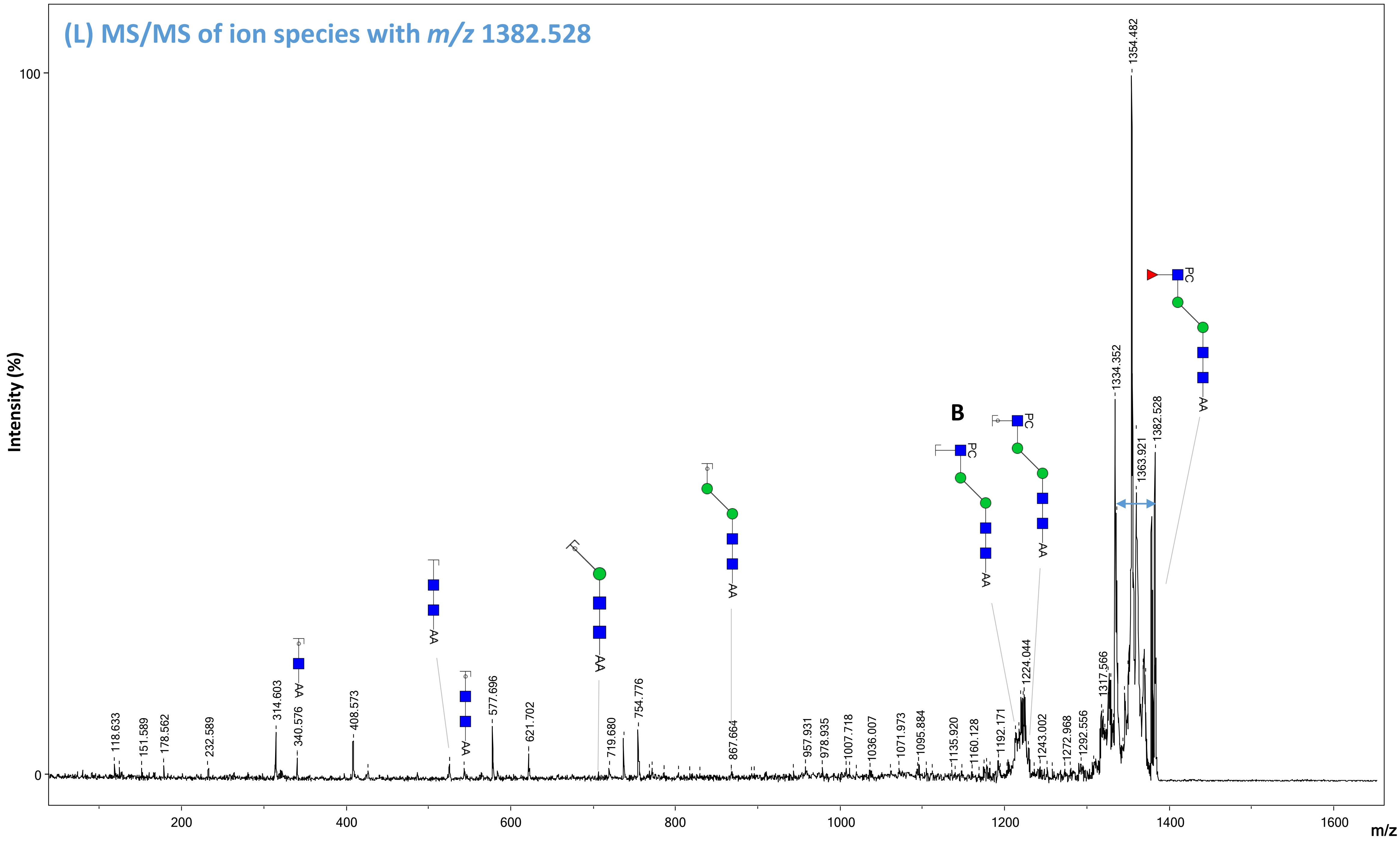
(L) MS/MS of ion species with m/z 1382.528
Intensity (%)
100
0
m/z
200
400
600
800
1000
1200
1400
1600
1354.482
1334.352
1382.528
1363.921
1317.566
1224.044
1243.002
1272.968
1292.556
1192.171
1160.128
1135.920
1095.884
1071.973
1036.007
1007.718
978.935
957.931
867.664
754.776
719.680
621.702
577.696
408.573
340.576
314.603
232.589
178.562
151.589
118.633
B
PC
AA

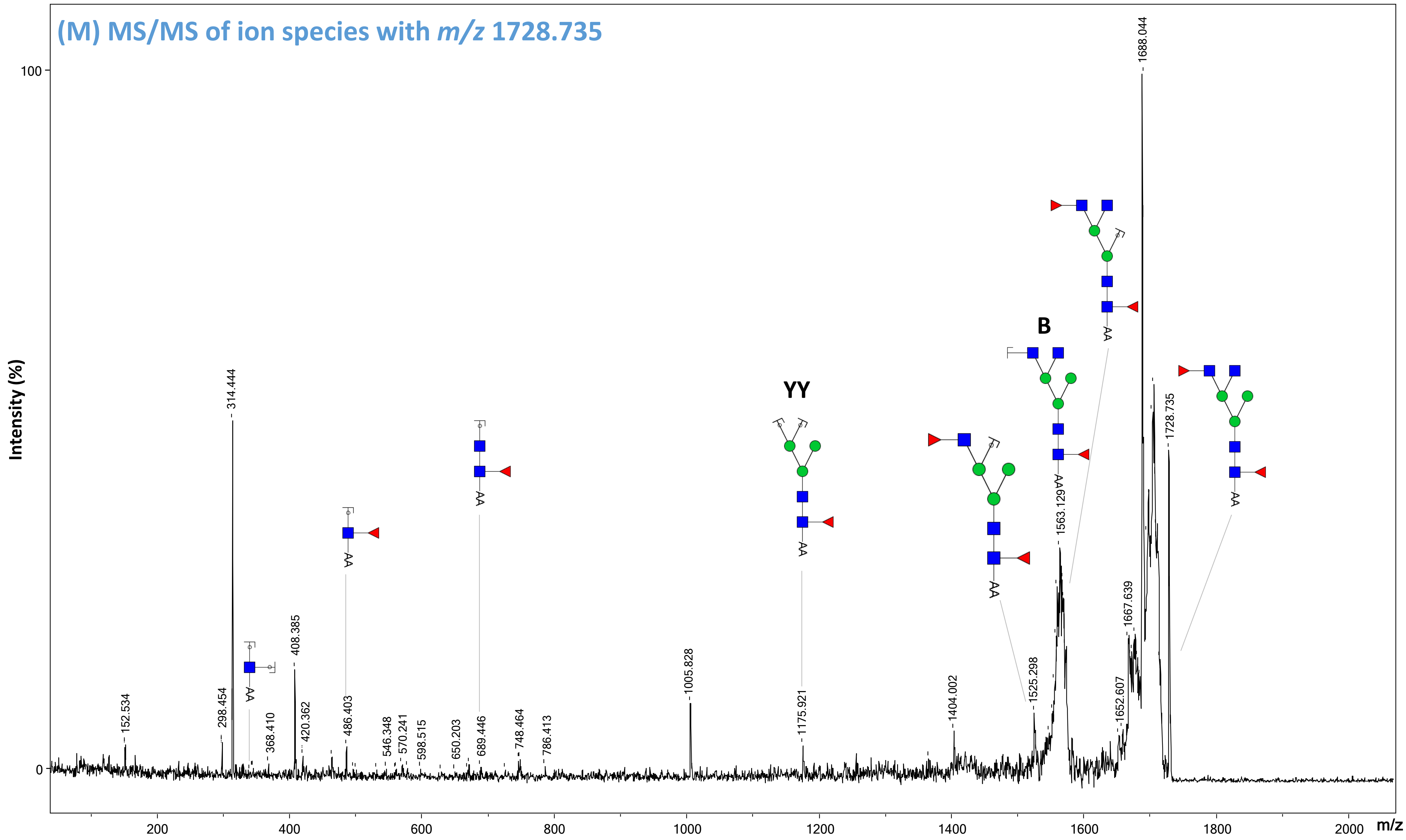
(M) MS/MS of ion species with m/z 1728.735
Intensity (%)
100
0
m/z
200
400
600
800
1000
1200
1400
1600
1800
2000
152.534
298.454
314.444
368.410
408.385
420.362
486.403
546.348
570.241
598.515
650.203
689.446
748.464
786.413
1005.828
1175.921
1404.002
1525.298
1563.129
1652.607
1667.639
1688.044
1728.735
YY
B
AA

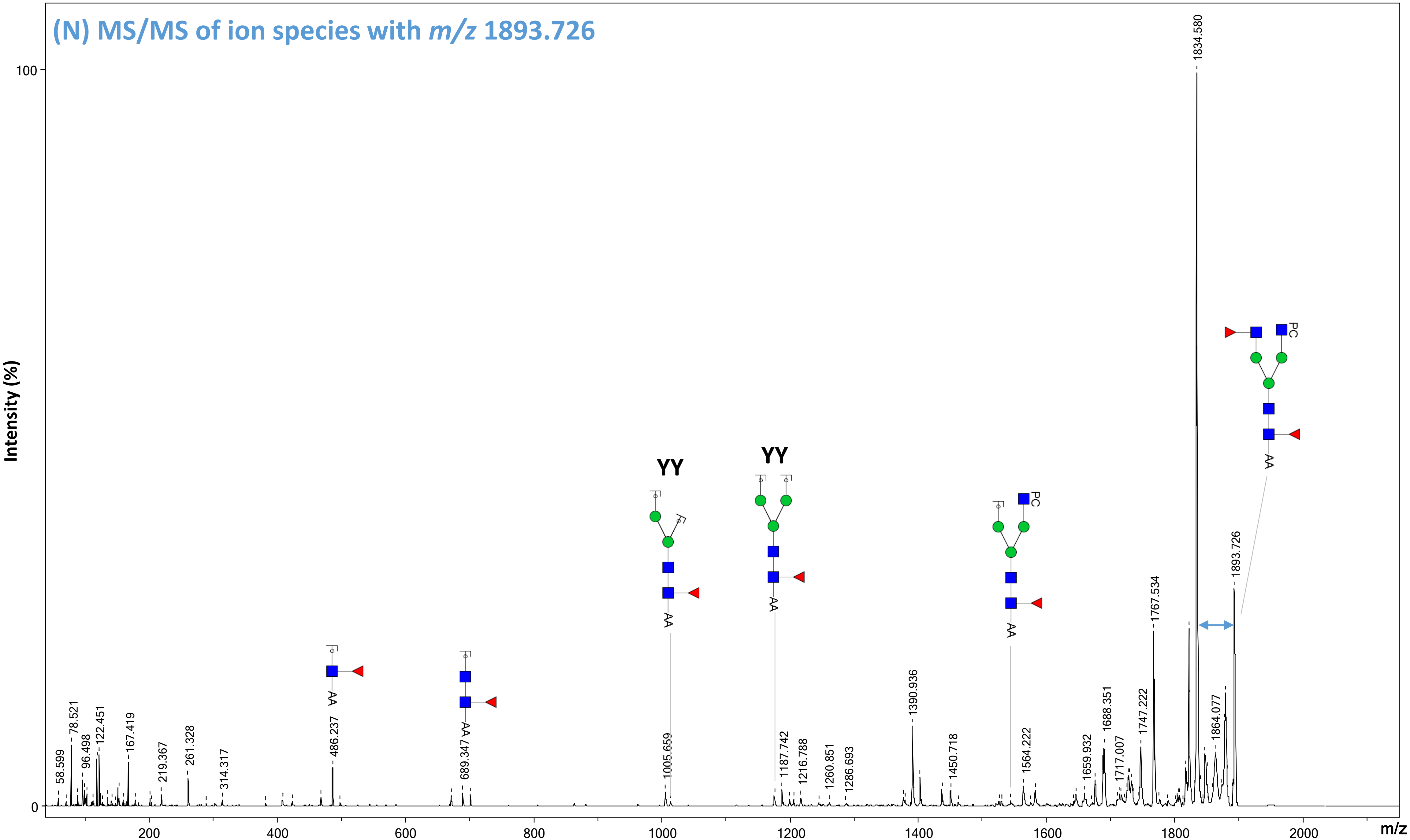
(N) MS/MS of ion species with m/z 1893.726
Intensity (%)
m/z
100
0
200
400
600
800
1000
1200
1400
1600
1800
2000
1834.580
1893.726
1767.534
1747.222
1717.007
1688.351
1659.932
1864.077
1564.222
1450.718
1390.936
1286.693
1260.851
1216.788
1187.742
1005.659
689.347
486.237
314.317
261.328
219.367
167.419
122.451
96.498
78.521
58.599
YY
YY
AA
PC

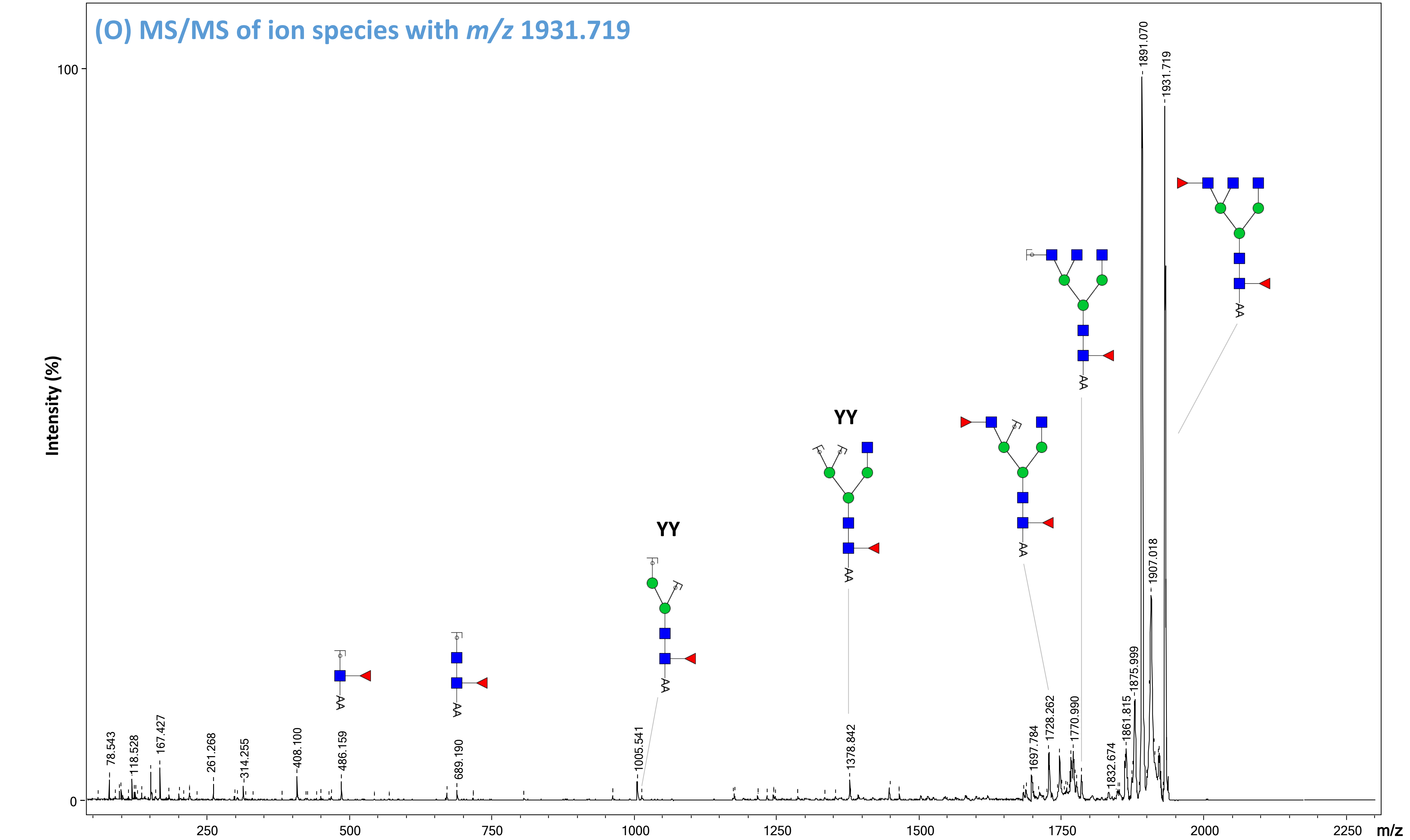
(O) MS/MS of ion species with m/z 1931.719
Intensity (%)
100
0
m/z
250
500
750
1000
1250
1500
1750
2000
2250
YY
YY
AA
78.543
118.528
167.427
261.268
314.255
408.100
486.159
689.190
1005.541
1378.842
1697.784
1728.262
1770.990
1832.674
1861.815
1875.999
1891.070
1907.018
1931.719

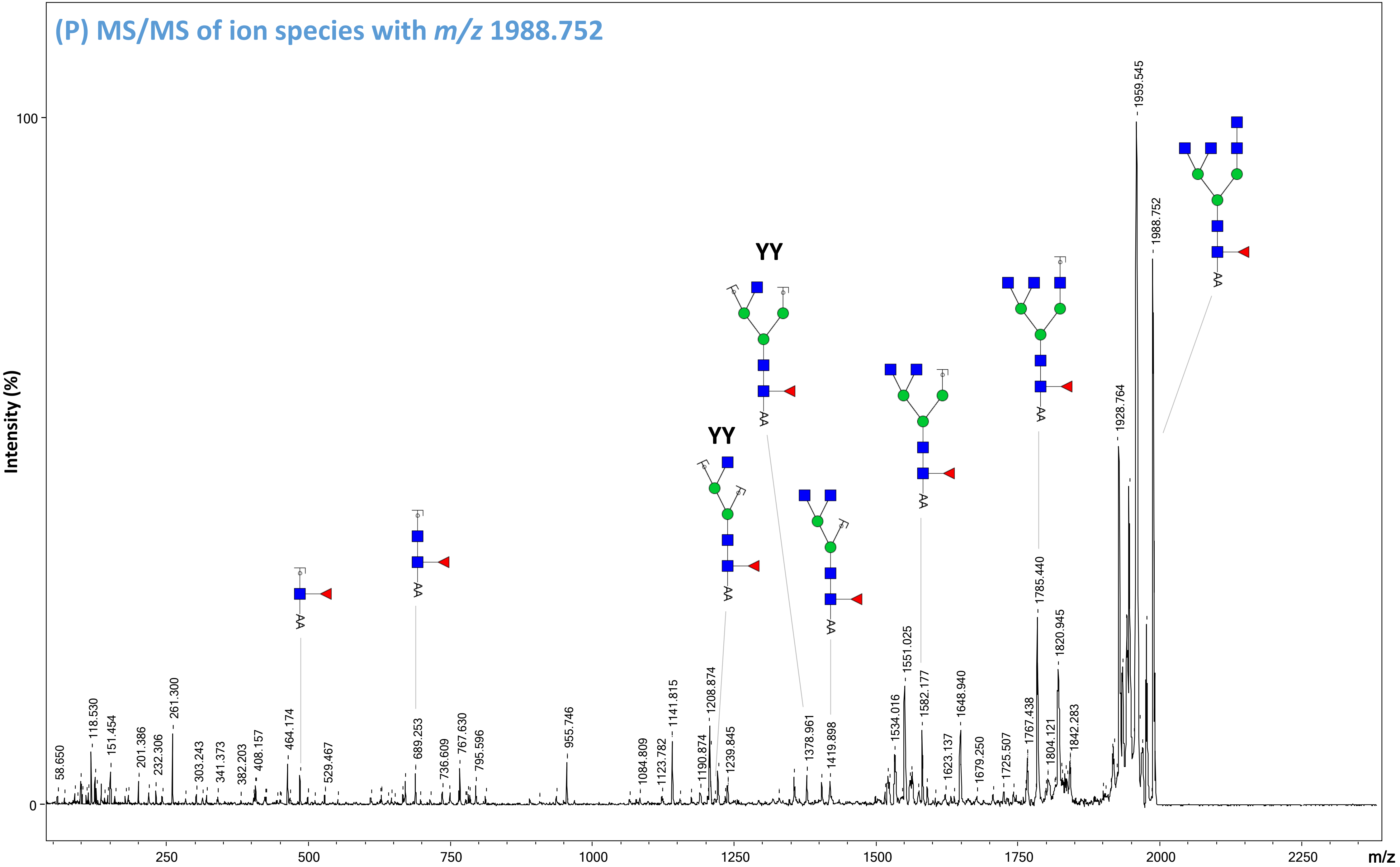

(P) MS/MS of ion species with m/z 1988.752
Intensity (%)
m/z
100
0
250
500
750
1000
1250
1500
1750
2000
2250
YY
YY
AA
58.650
118.530
151.454
201.386
232.306
261.300
303.243
341.373
382.203
408.157
464.174
529.467
689.253
736.609
767.630
795.596
955.746
1084.809
1123.782
1141.815
1190.874
1208.874
1239.845
1378.961
1419.898
1534.016
1551.025
1582.177
1623.137
1648.940
1679.250
1725.507
1767.438
1785.440
1804.121
1820.945
1842.283
1928.764
1959.545
1988.752

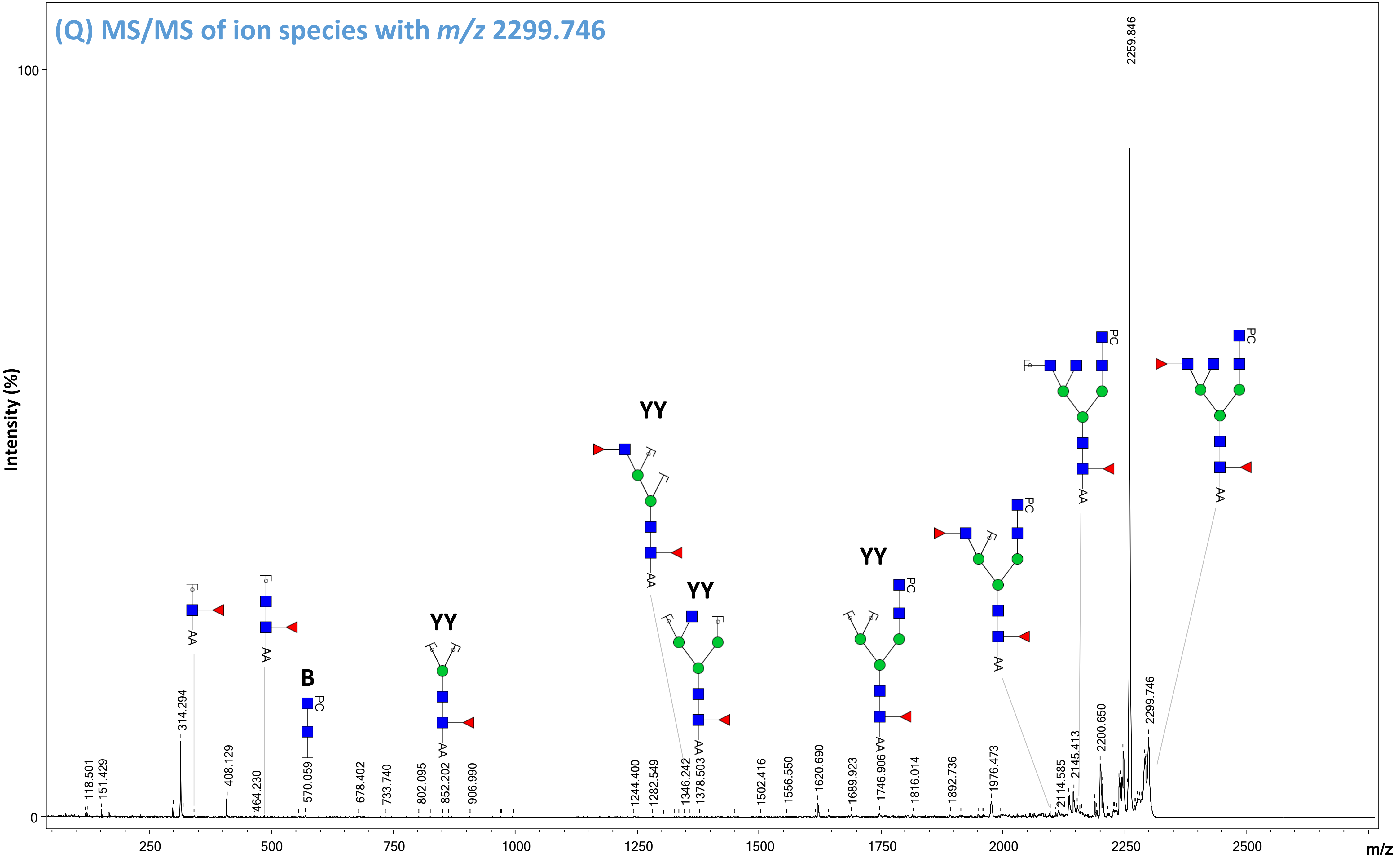
(Q) MS/MS of ion species with m/z 2299.746
Intensity (%)
100
0
m/z
250
500
750
1000
1250
1500
1750
2000
2250
2500
118.501
151.429
314.294
408.129
464.230
570.059
678.402
733.740
802.095
852.202
906.990
1244.400
1282.549
1346.242
1378.503
1502.416
1556.550
1620.690
1689.923
1746.906
1816.014
1892.736
1976.473
2114.585
2145.413
2200.650
2259.846
2299.746
B
YY
YY
YY
YY
AA
PC

Figure S3 - Glycan sequencing of selected *B. malayi* GSL glycans

MALDI-TOF-MS of exoglycosidase and HF treated *B. malayi* GSL glycans (A-J) EGCase-released and AA-labeled total (A-C) and UHPLC-purified (D-J) *B. malayi* GSL glycans were subjected to various exoglycosidase digestions and/or incubation with hydrofluoric acid (HF). Protocols are detailed in the Experimental Procedures section and a list of the exoglycosidases used and corresponding reaction conditions are available in **Table S1**. Treatments performed on the GSL glycans are indicated at the top of each panel and blue arrows highlight the products resulting from the aforementioned treatments.

MALDI-TOF-MS/MS of selected *B. malayi* GSL glycan ions species (K-O) Selected ions (*m/z* indicated in the upper left corner) were subjected to fragmentation analysis. Resulting spectra are labeled with graphic representation of Y-type ions, unless indicated otherwise (B = B-type, C = C-type, Z = Z-type). Losses of a mass of 59 Da from the parent ion is indicative of loss of a PC (Refs #56, Timm, T. *et al.* and #57, Grabitzki, J. *et al.*) and is highlighted by a blue double arrow when necessary.

For all MALDI-TOF-MS and MS/MS spectra, measurements were acquired in negative-ion reflectron mode and signals are labeled with monoisotopic masses (*m/z*). AA-labeled glycans are represented using the CFG nomenclature: blue square = N-acetylglucosamine, green circle = mannose, PC = phosphorylcholine, red triangle = fucose, white and blue diamond = glucuronic acid, yellow circle = galactose and yellow square = N-acetylgalactosamine.

(A) Exoglycosidase digestions of adult female total GSL glycan pool (m/z range = 800 to 1500)

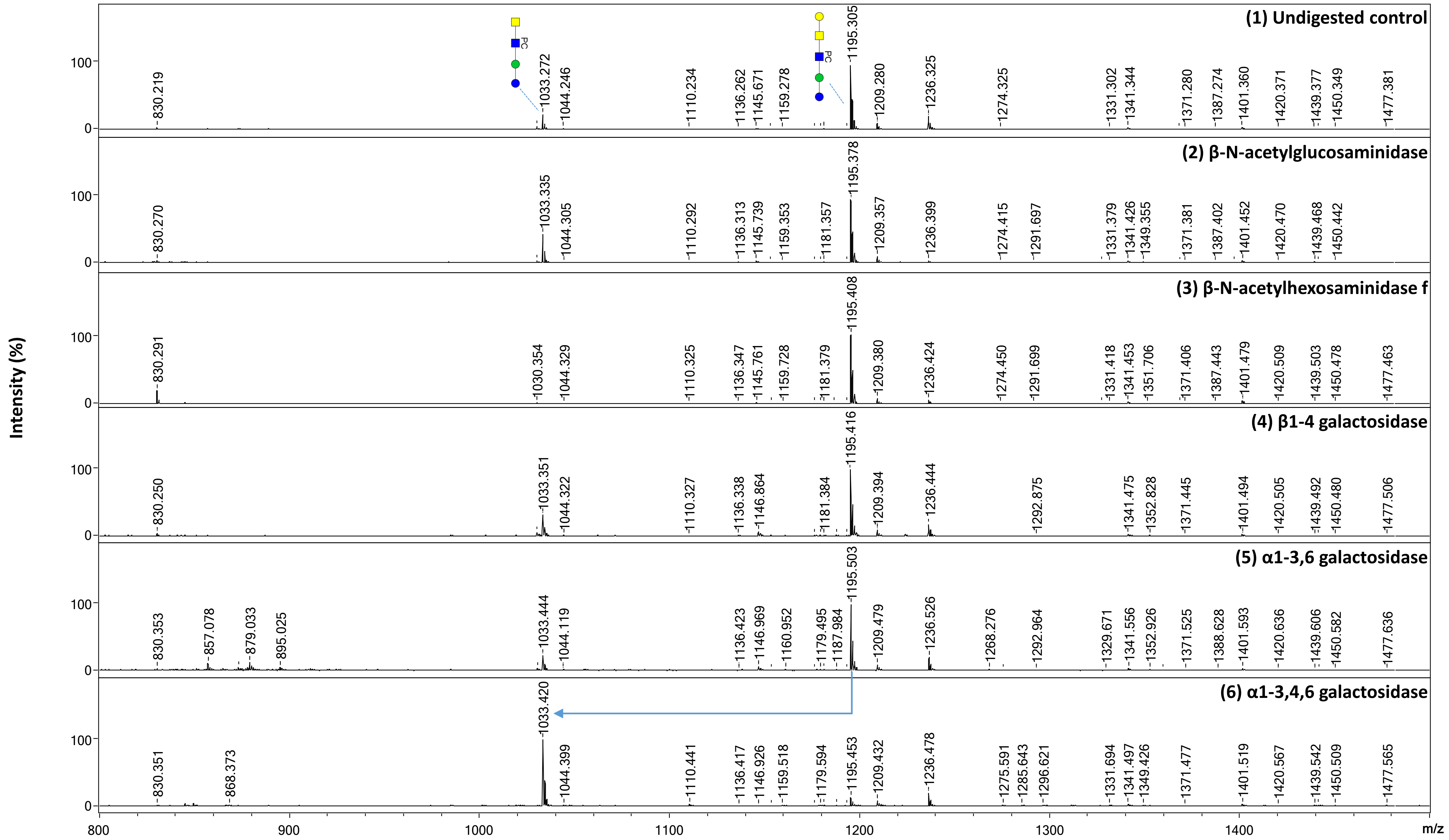

(B) Exoglycosidase digestions of adult female total GSL glycan pool (m/z range = 800 to 1200)

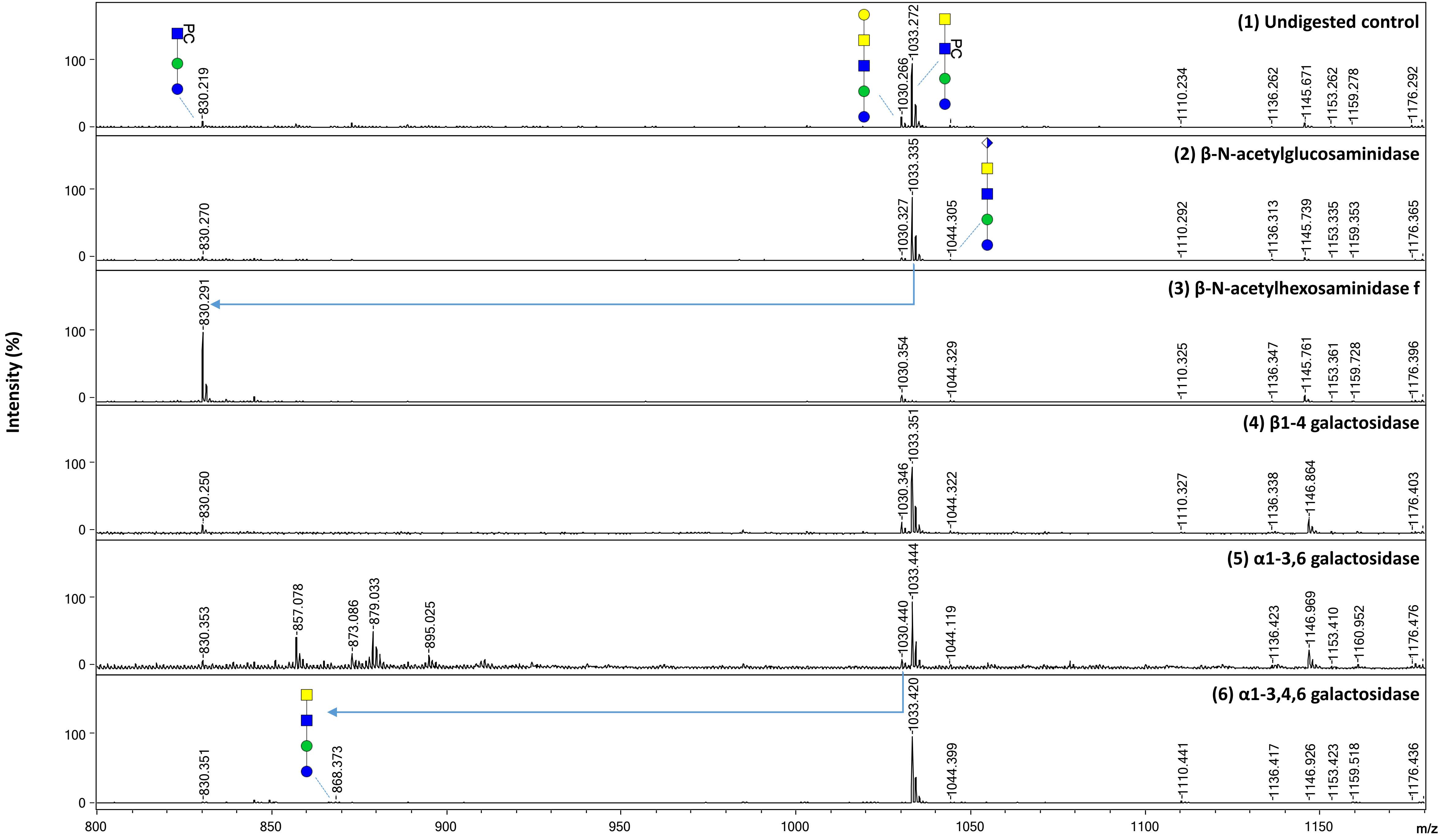

(C) Exoglycosidase digestions of adult female total GSL glycan pool (m/z range = 1200 to 1700)

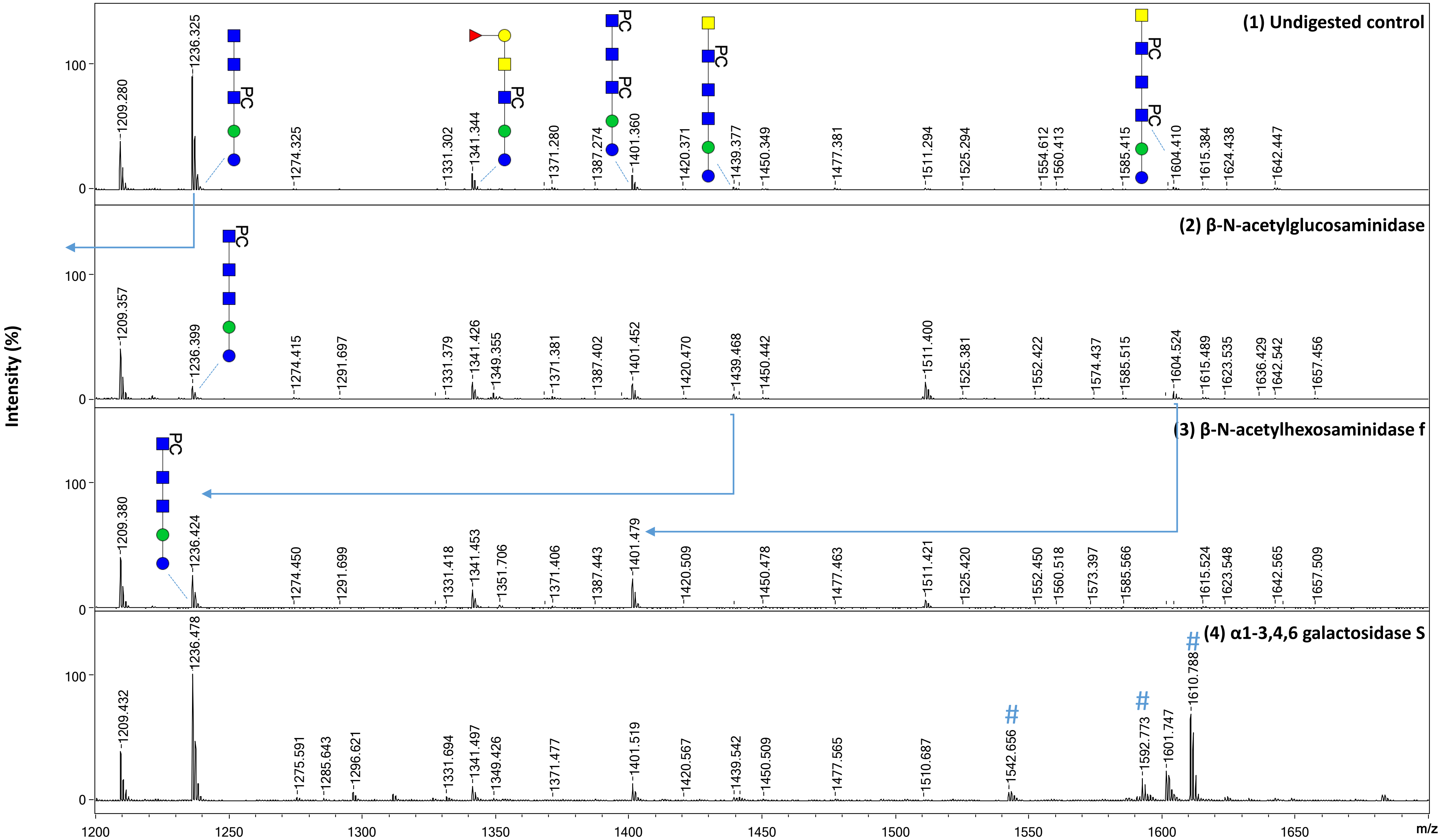

(D) Exoglycosidase digestions of purified fraction Lg5

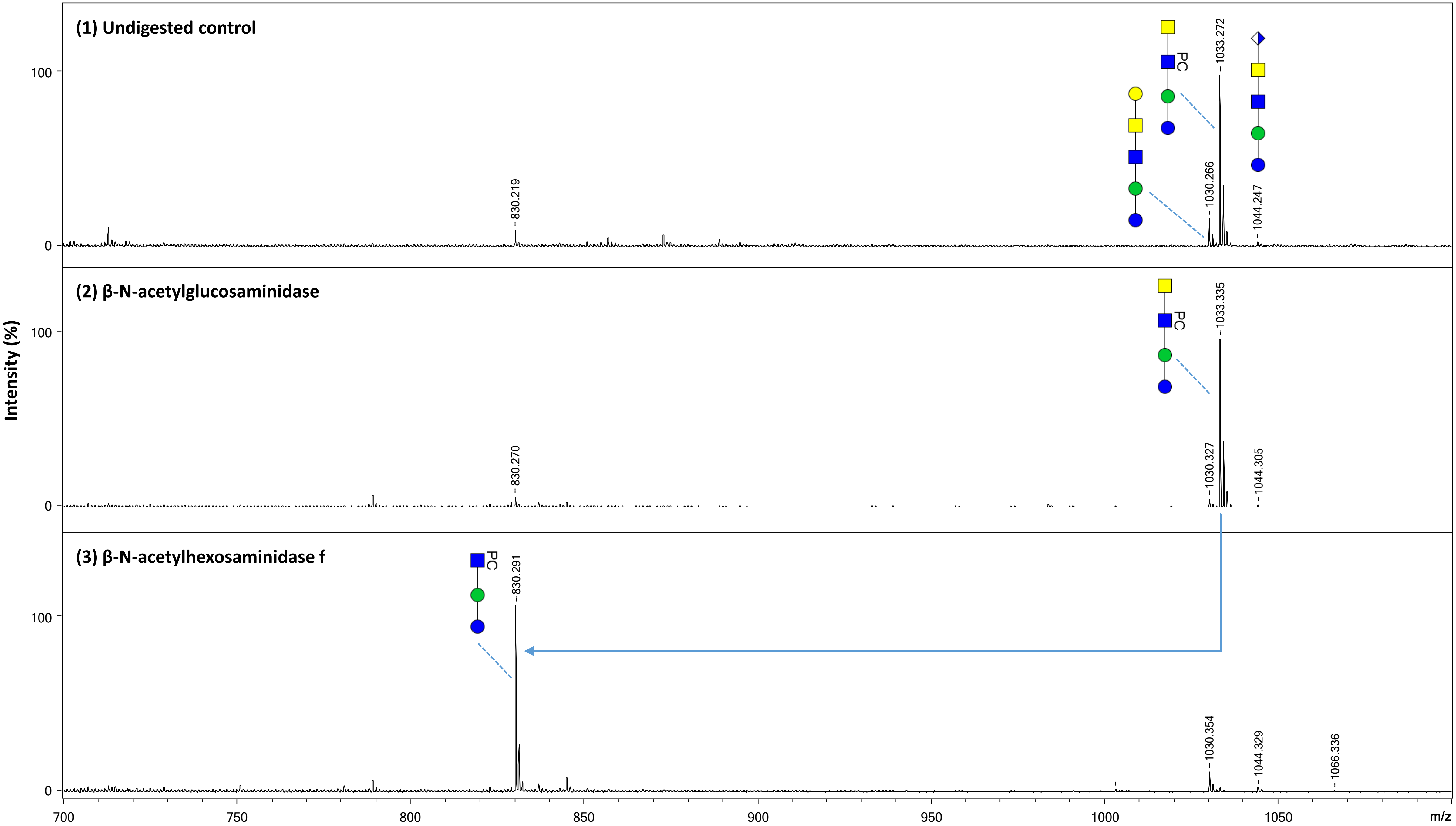

(E) Exoglycosidase digestions of purified fraction Lg2

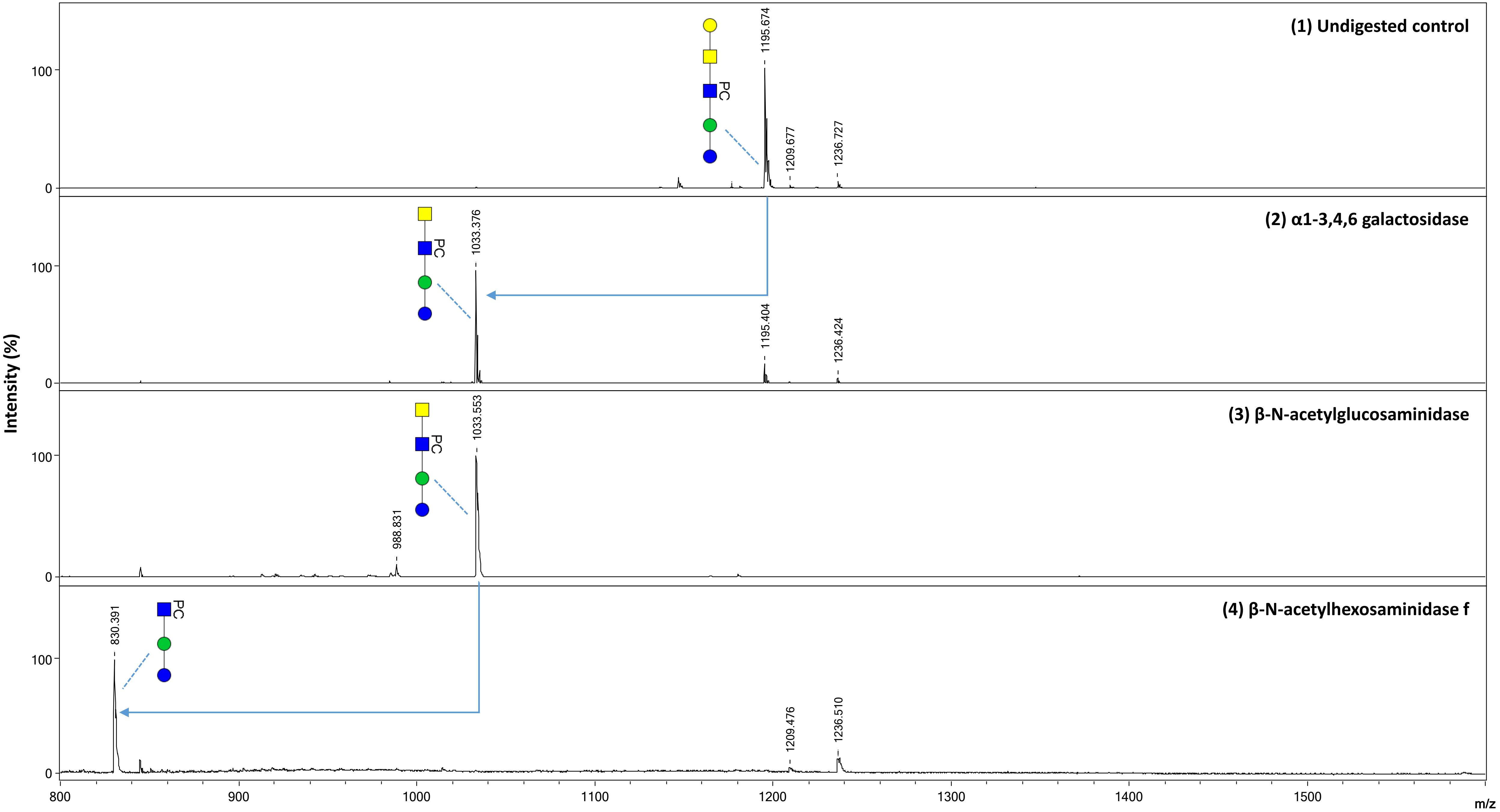

(F) HF treatment and sequential exoglycosidase digestions of purified fraction Lg7

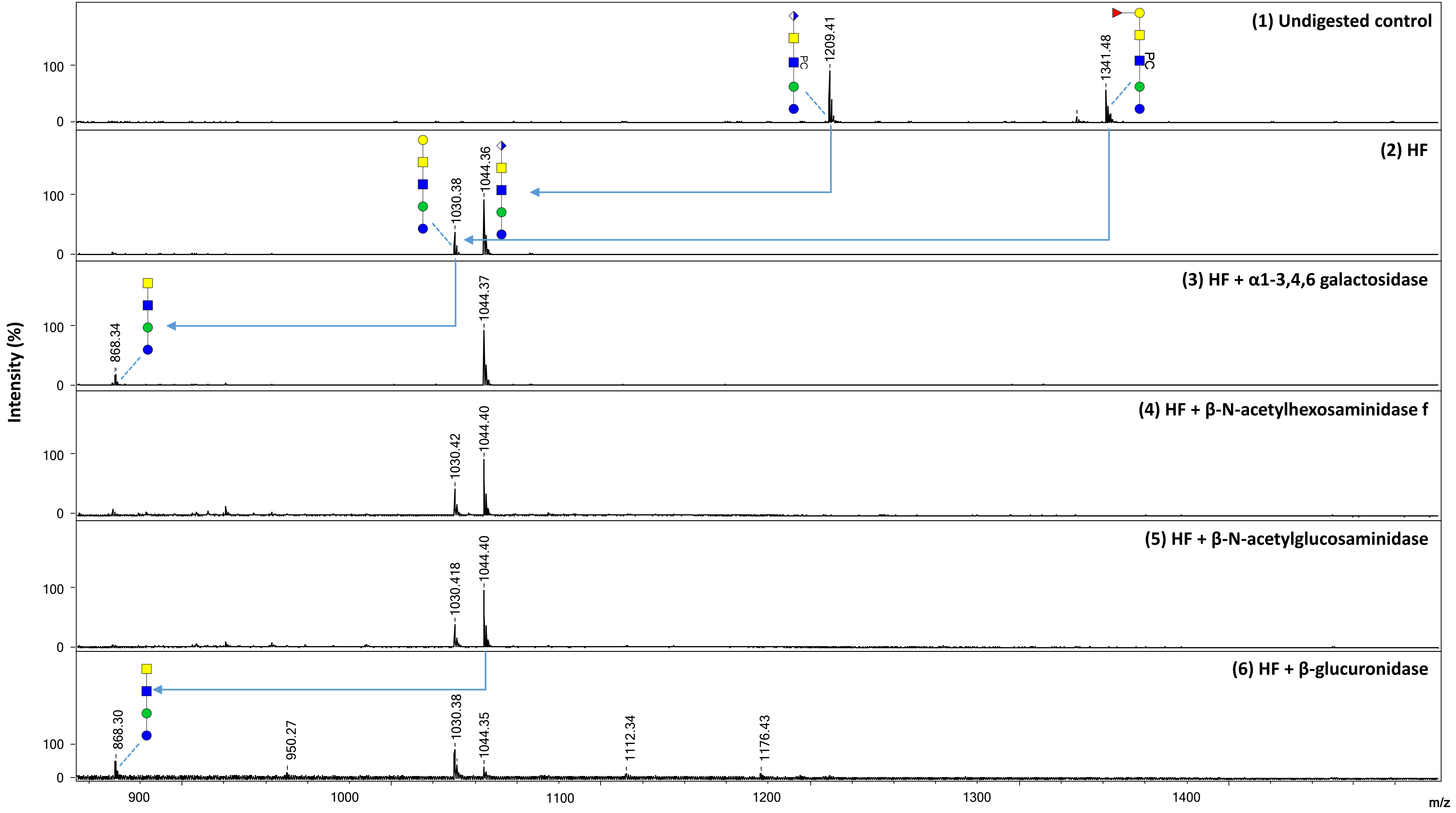

(G) β-N-acetylhexosaminidase digestion of purified fraction (not selected for array)

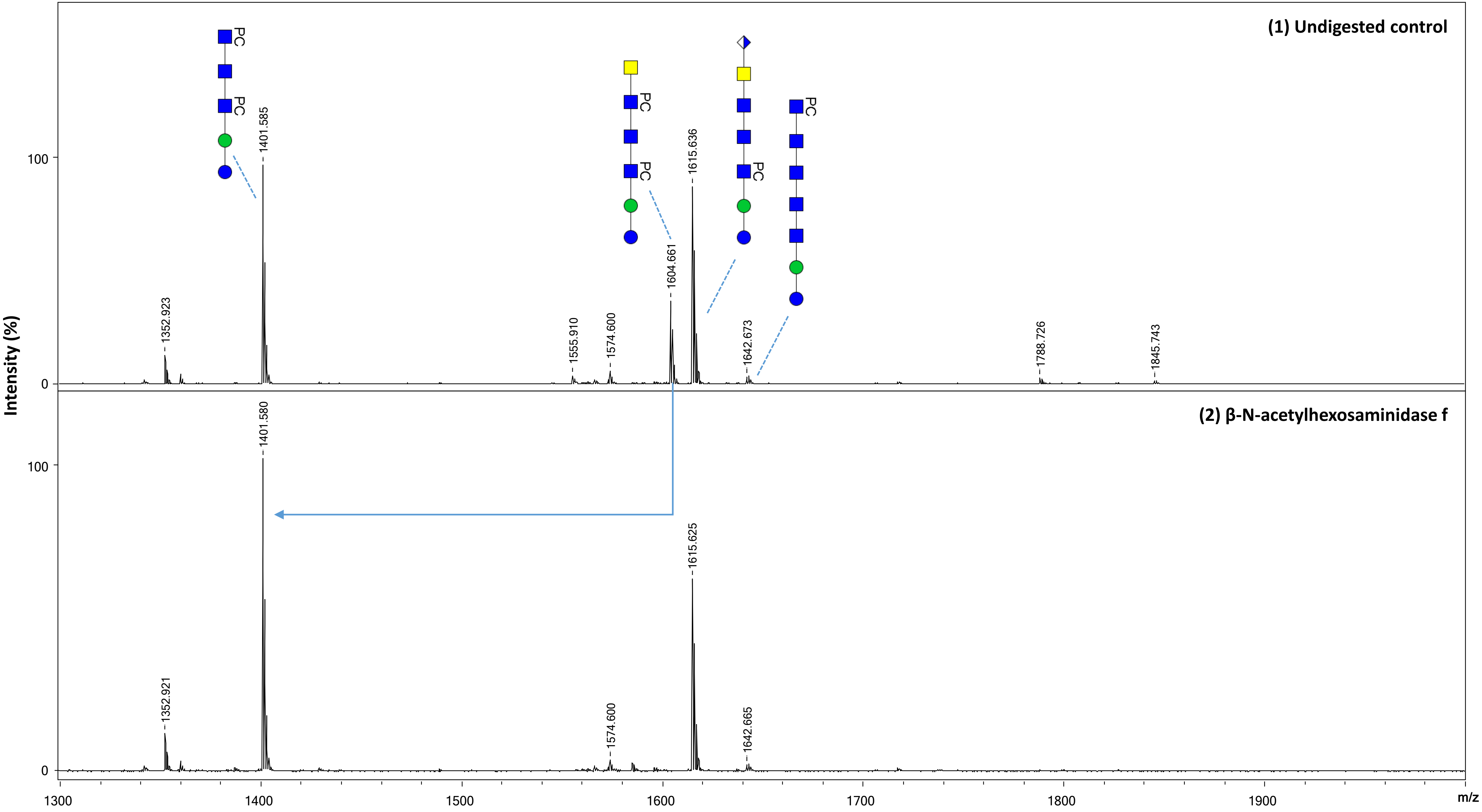

(H) Exoglycosidase digestions of purified fraction Lg8

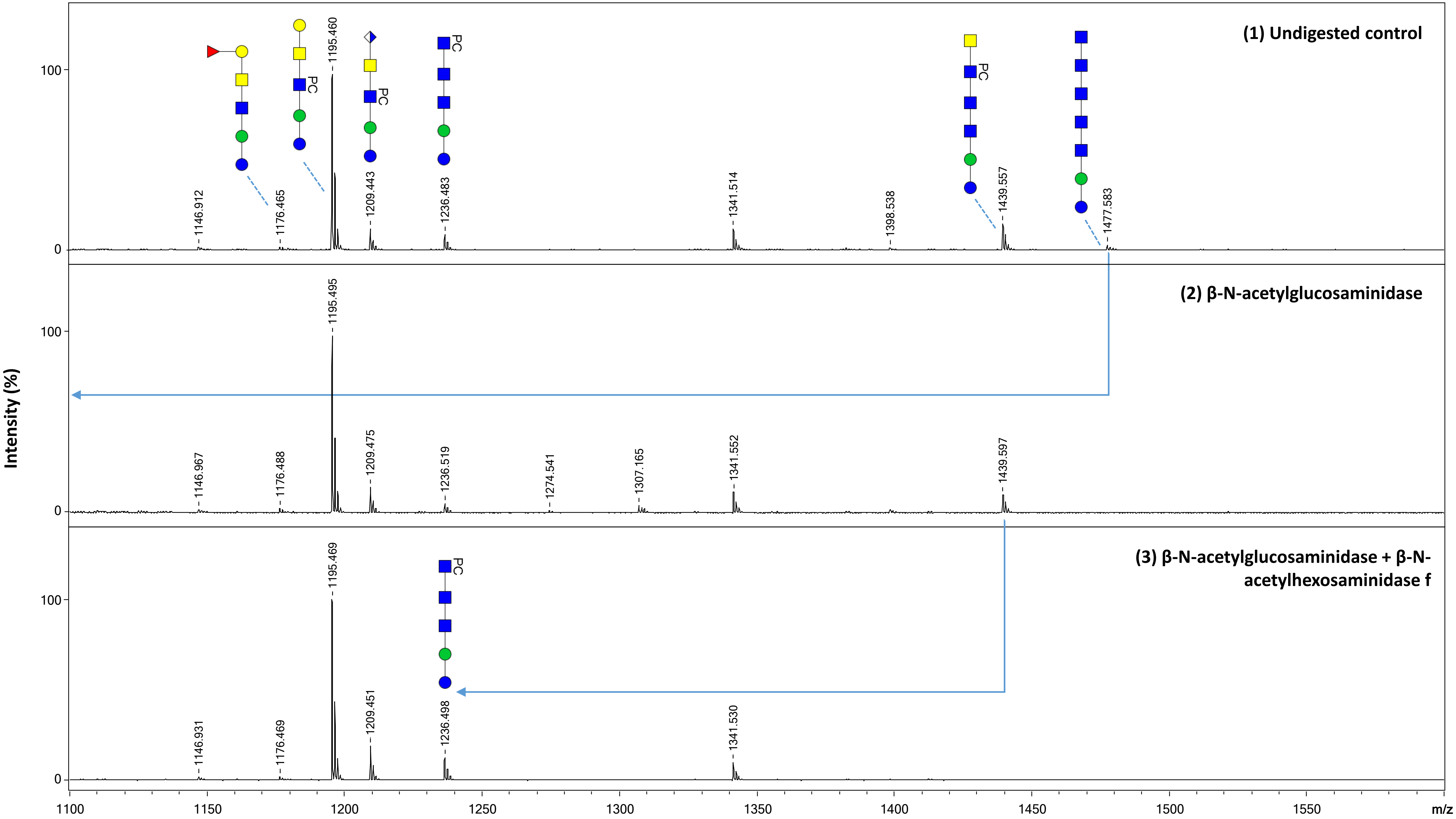

(I) HF treatment of purified fraction Lg11

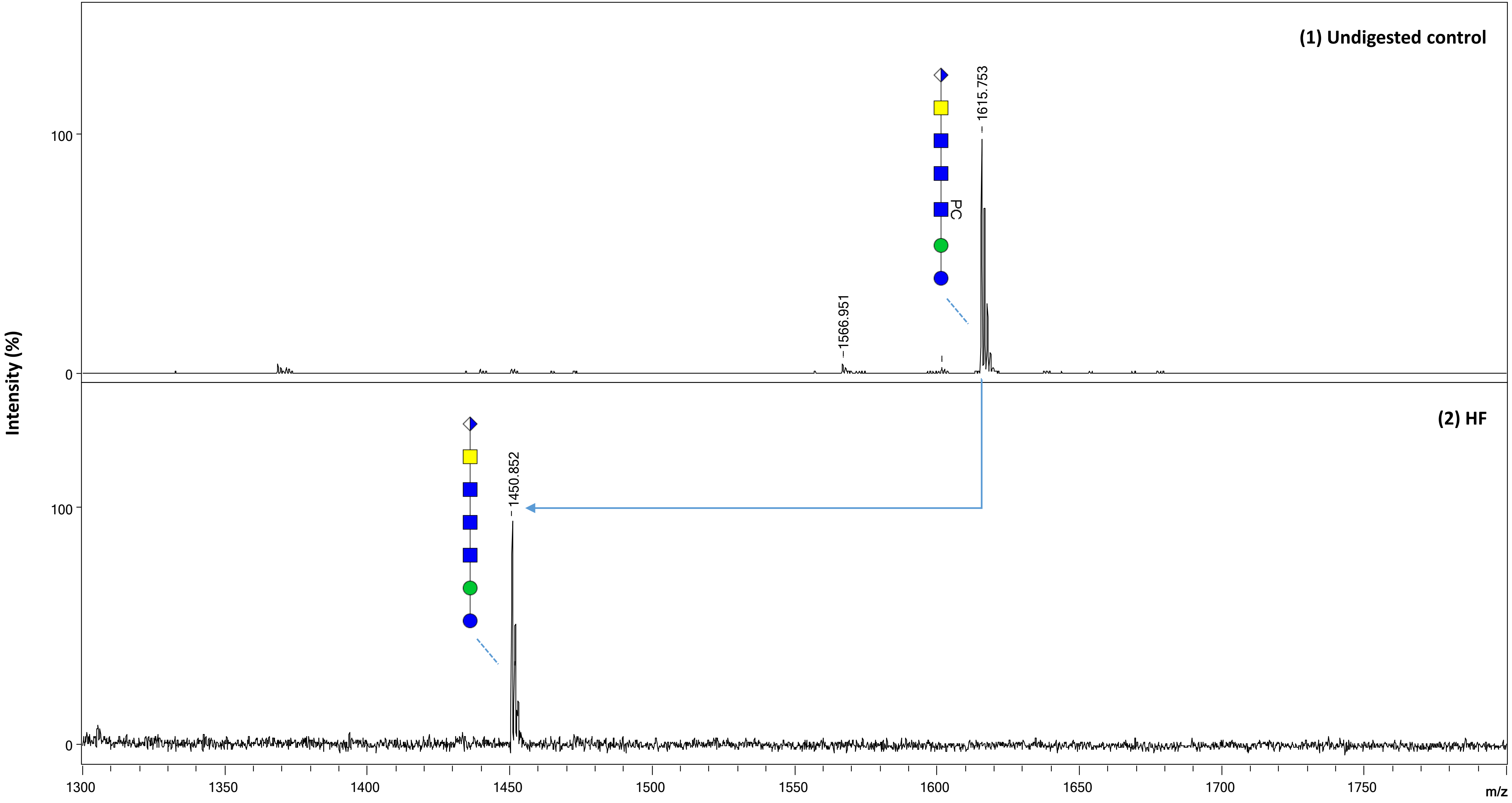

(J) HF treatment and sequential β-glucuronidase digestion of purified fraction Lg12

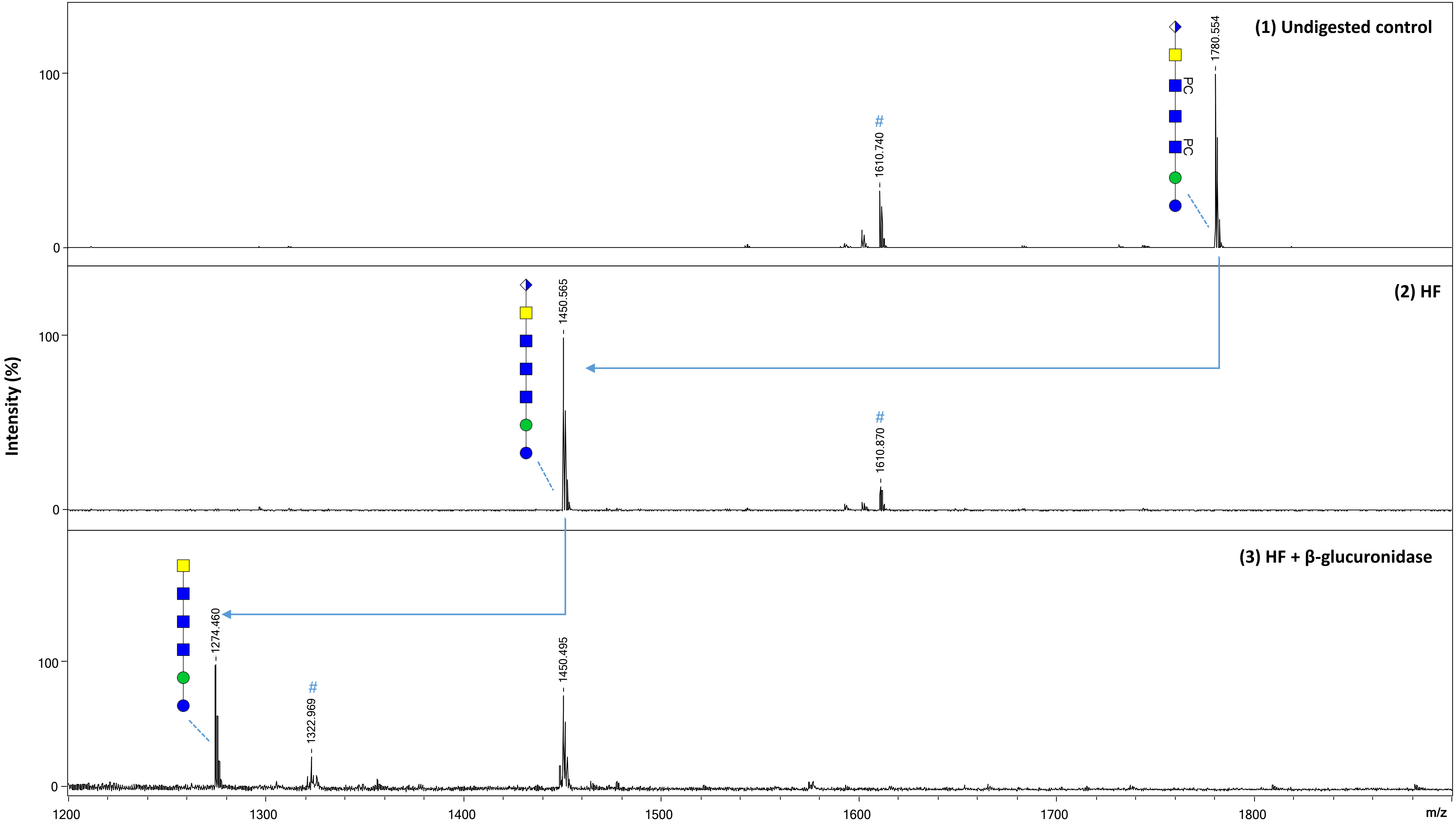

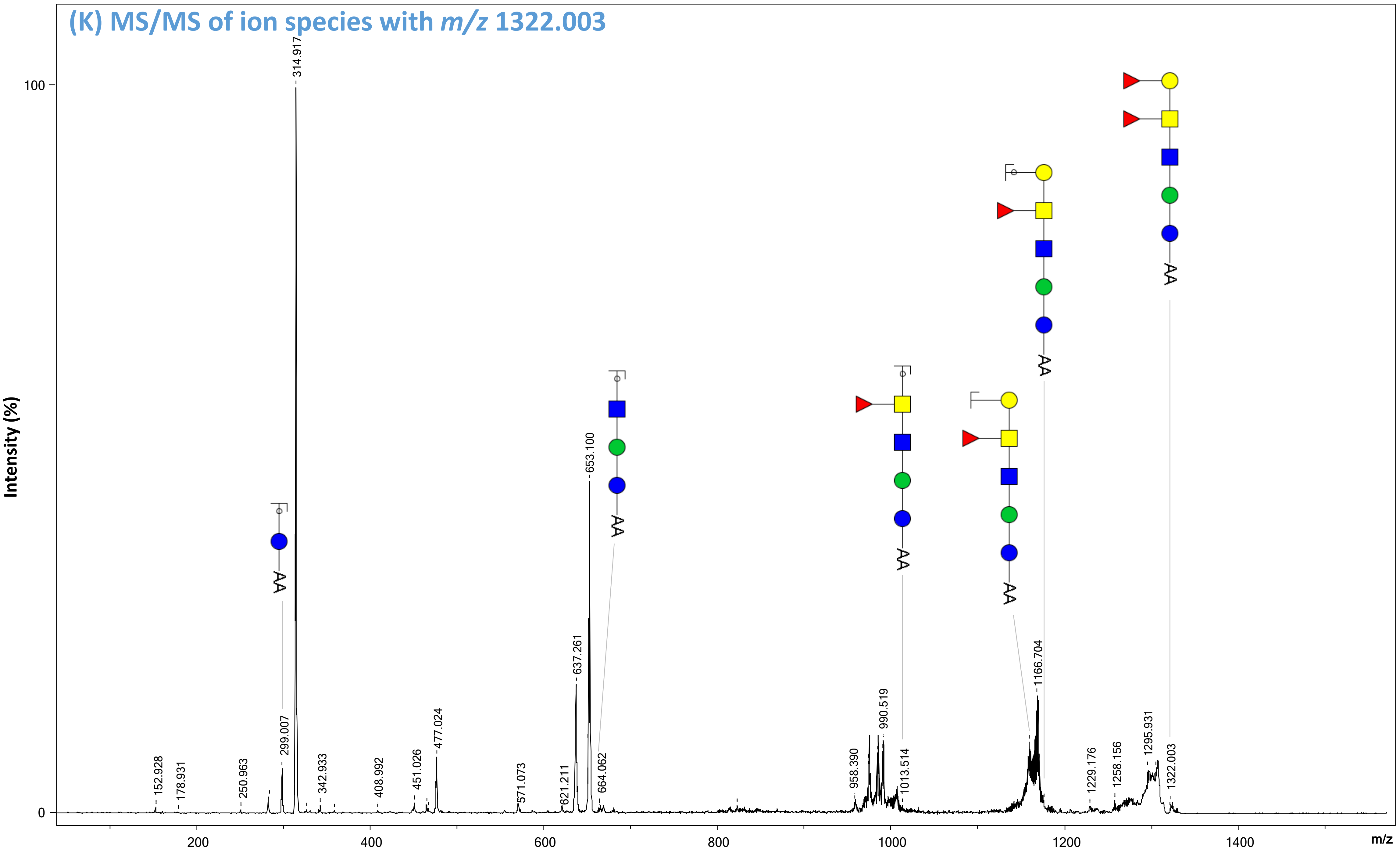
(K) MS/MS of ion species with m/z 1322.003
Intensity (%)
100
0
314.917
653.100
637.261
1166.704
990.519
1295.931
477.024
299.007
1322.003
451.026
1258.156
1229.176
1013.514
958.390
664.062
571.073
621.211
342.933
408.992
250.963
178.931
152.928
AA
200
400
600
800
1000
1200
1400
m/z

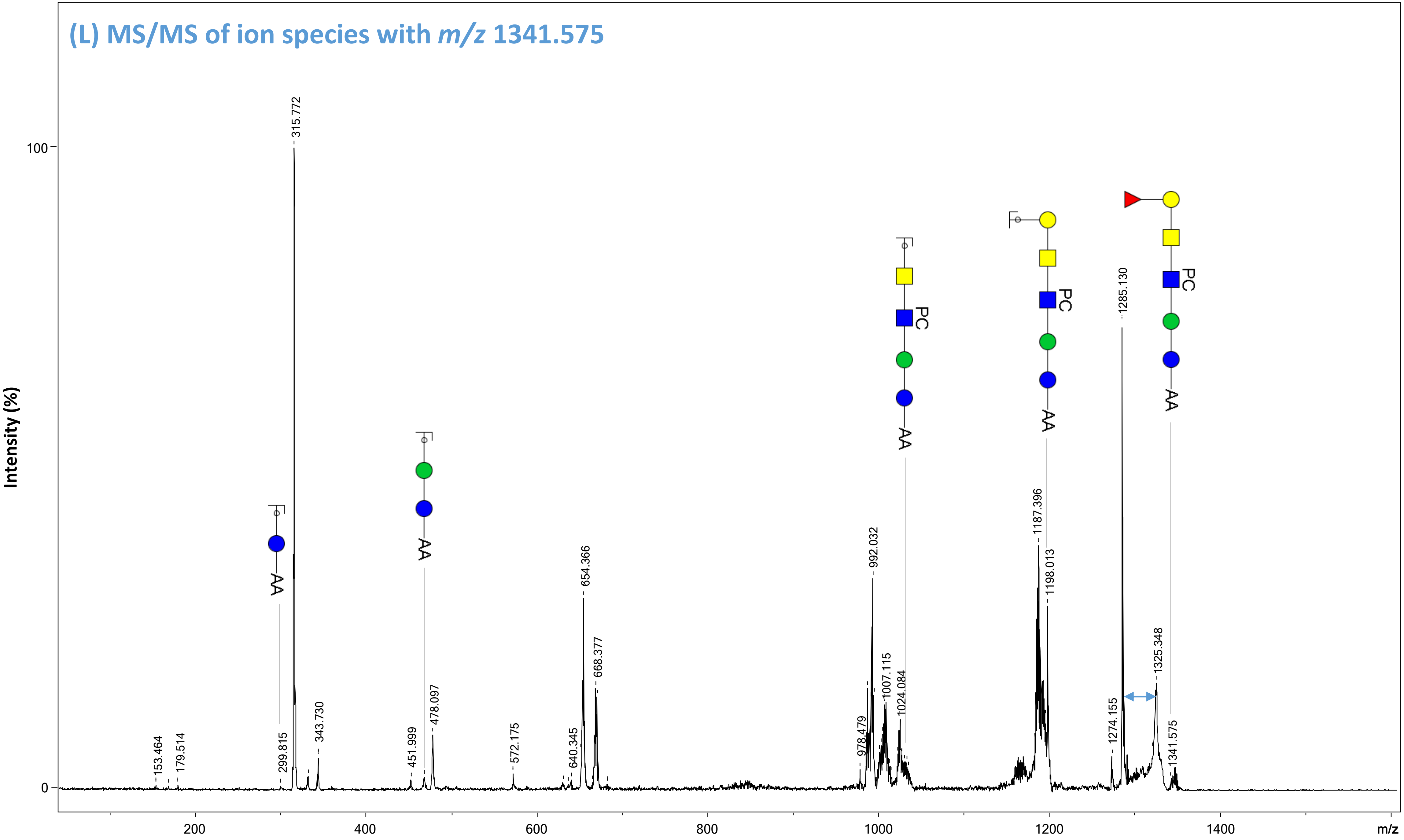
(L) MS/MS of ion species with m/z 1341.575
Intensity (%)
100
0
315.772
153.464
179.514
299.815
343.730
451.999
478.097
572.175
640.345
654.366
668.377
978.479
992.032
1007.115
1024.084
1187.396
1198.013
1274.155
1285.130
1325.348
1341.575
AA
PC
200
400
600
800
1000
1200
1400
m/z

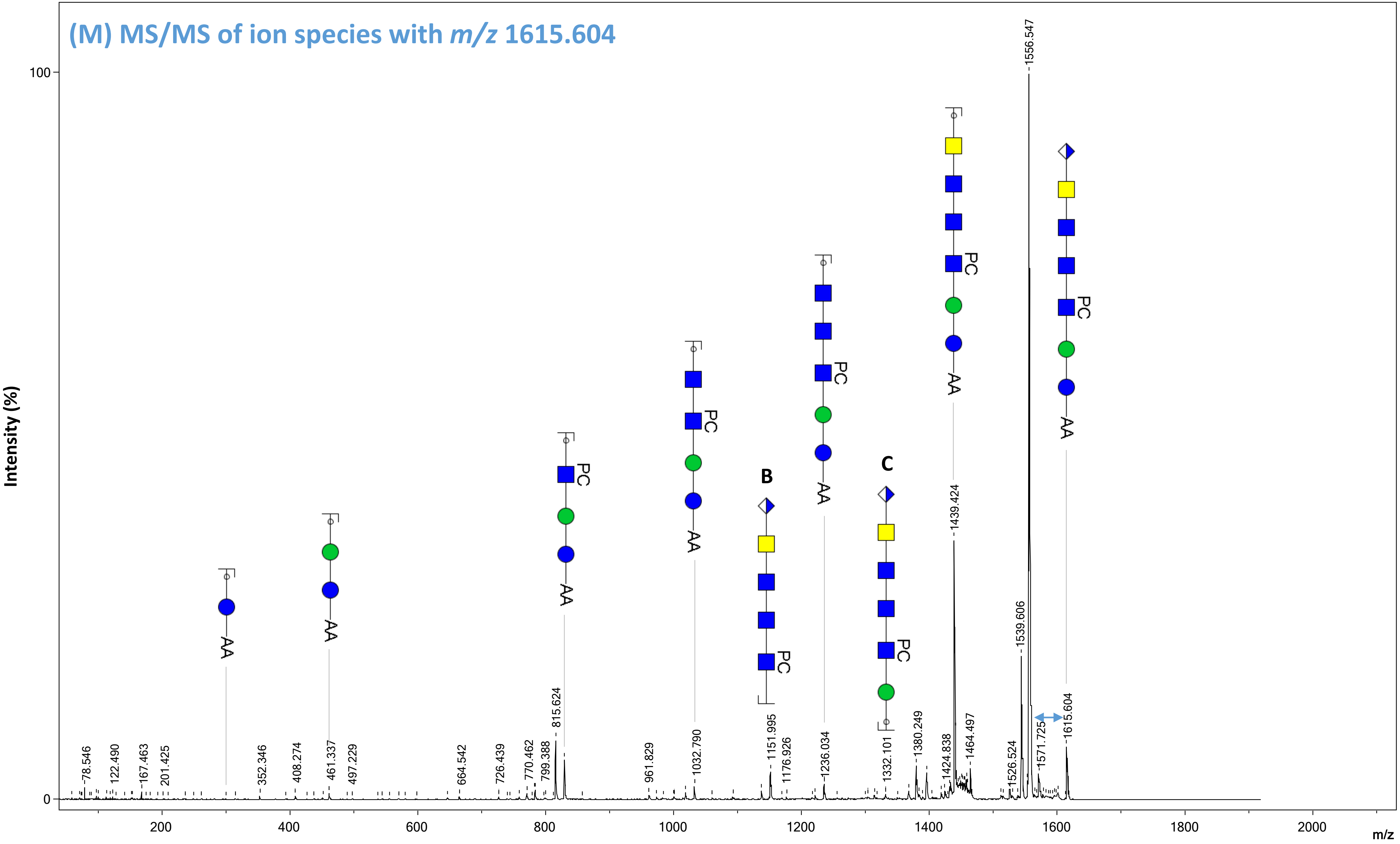
(M) MS/MS of ion species with m/z 1615.604
Intensity (%)
100
0
m/z
200
400
600
800
1000
1200
1400
1600
1800
2000
B
C
PC
AA
78.546
122.490
167.463
201.425
352.346
408.274
461.337
497.229
664.542
726.439
770.462
799.388
815.624
961.829
1032.790
1151.995
1176.926
1236.034
1332.101
1380.249
1424.838
1439.424
1464.497
1526.524
1539.606
1556.547
1571.725
1615.604

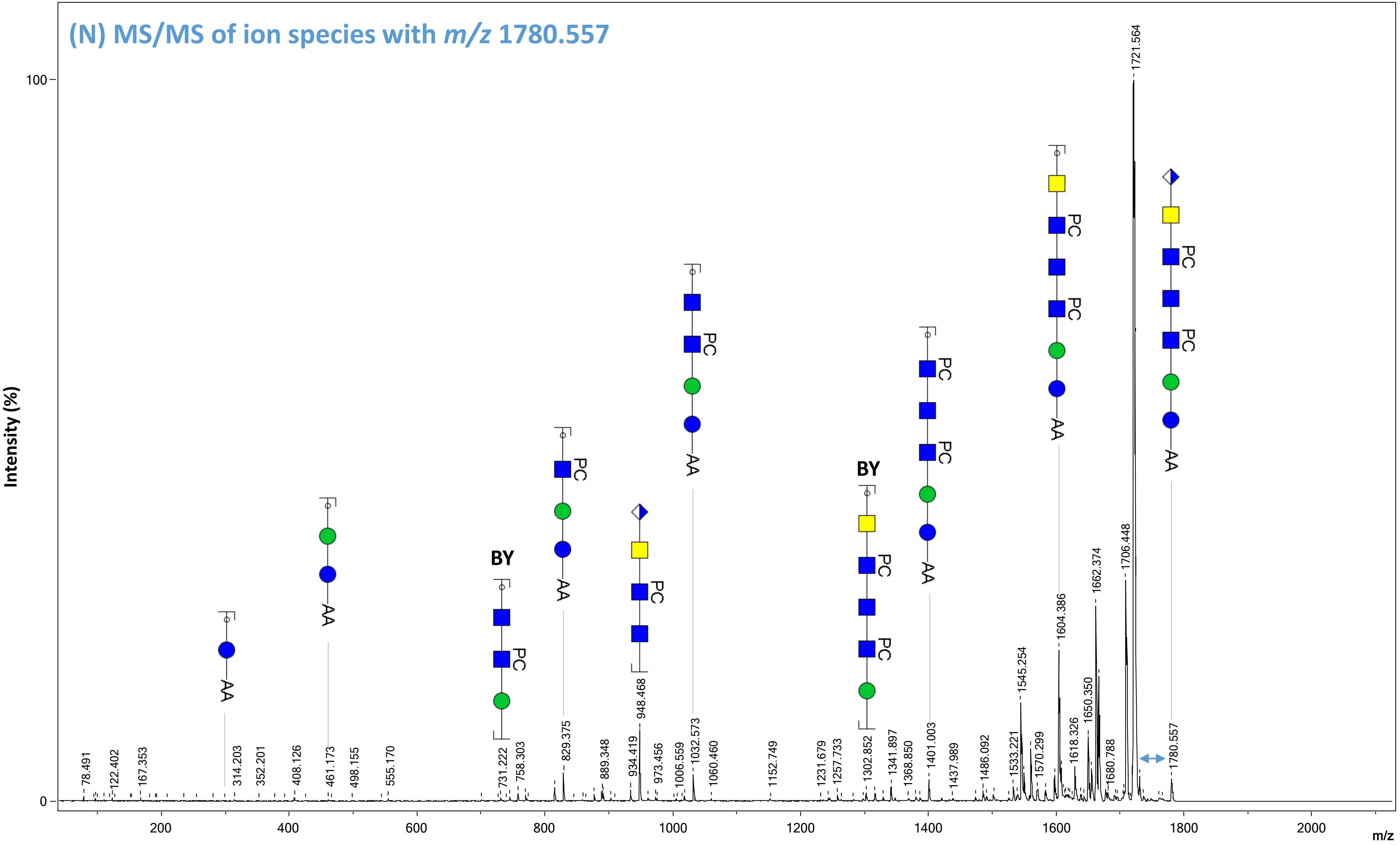
(N) MS/MS of ion species with m/z 1780.557
Intensity (%)
100
0
m/z
200
400
600
800
1000
1200
1400
1600
1800
2000
BY
BY
PC
AA
78.491
122.402
167.353
314.203
352.201
408.126
461.173
498.155
555.170
731.222
758.303
829.375
889.348
934.419
948.468
973.456
1006.559
1032.573
1060.460
1152.749
1231.679
1257.733
1302.852
1341.897
1368.850
1401.003
1437.989
1486.092
1533.221
1545.254
1570.299
1604.386
1618.326
1650.350
1662.374
1680.788
1706.448
1721.564
1780.557

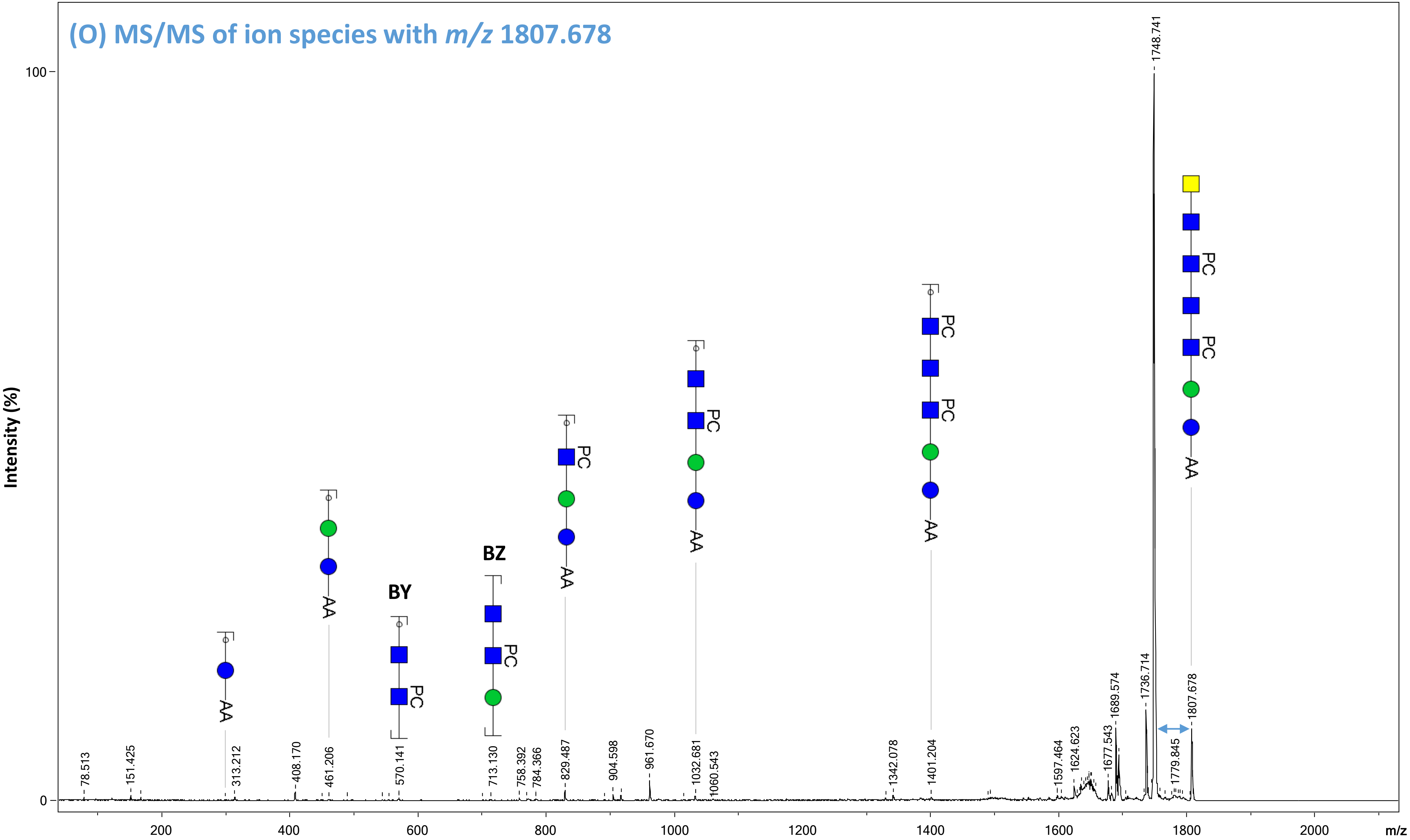
(O) MS/MS of ion species with m/z 1807.678
Intensity (%)
100
0
m/z
200
400
600
800
1000
1200
1400
1600
1800
2000
78.513
151.425
313.212
408.170
461.206
570.141
713.130
758.392
784.366
829.487
904.598
961.670
1032.681
1060.543
1342.078
1401.204
1597.464
1624.623
1677.543
1689.574
1736.714
1748.741
1779.845
1807.678
BY
BZ
AA
PC

Figure S4 - Glycan array screening with monoclonal antibodies for array validation

Binding to the *B. malayi* glycan array was measured for the mAb 100-4G1 recognizing the trimannosyl branching R-α1-6Man(α1-6Man)α1-3Man **(A)** and for the PC-specific mAb M1421 **(B)**. Background corrected median fluorescence intensities (MFIs, y-axis) are shown for each glycan fraction printed on the array (x-axis). Fraction contents relevant to the mAb specificity are indicated below the fraction names on the x-axis. Blanks = no glycan content, print buffer only (negative controls); M3-9 = abbreviation used to designate mannosidic glycan structures of composition $GlcNAc_2Man_{3\text{-}9}$; GSLs + HF = HF treated GSL glycan containing fractions (*i.e.* with PC and fucoses removed); PC-GlcNAc = fractions containing N-glycan structures with PC-substitution(s) of terminal GlcNAcs(s); PC-Man = fractions containing N-glycan structures with PC-substitution of a mannose residue. Graph raw numerical data can be found in **Table S6**.

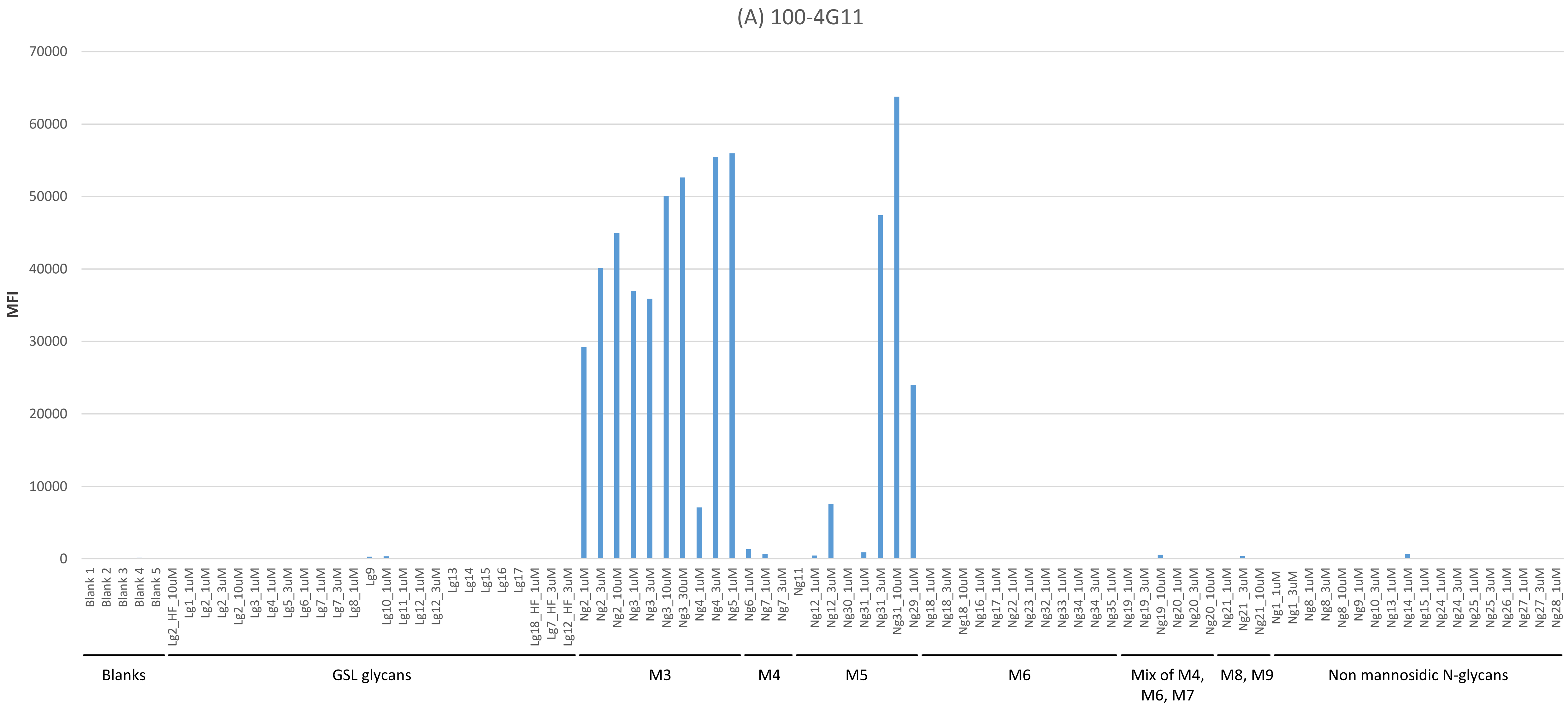
(A) 100-4G11
MFI
70000
60000
50000
40000
30000
20000
10000
0
Blank 1
Blank 2
Blank 3
Blank 4
Blank 5
Lg2_HF_10uM
Lg1_1uM
Lg2_1uM
Lg2_3uM
Lg2_10uM
Lg3_1uM
Lg4_1uM
Lg5_3uM
Lg6_1uM
Lg7_1uM
Lg7_3uM
Lg8_1uM
Lg9
Lg10_1uM
Lg11_1uM
Lg12_1uM
Lg12_3uM
Lg13
Lg14
Lg15
Lg16
Lg17
Lg18_HF_1uM
Lg7_HF_3uM
Lg12_HF_3uM
Ng2_1uM
Ng2_3uM
Ng2_10uM
Ng3_1uM
Ng3_3uM
Ng3_10uM
Ng3_30uM
Ng4_1uM
Ng4_3uM
Ng5_1uM
Ng6_1uM
Ng7_1uM
Ng7_3uM
Ng11
Ng12_1uM
Ng12_3uM
Ng30_1uM
Ng31_1uM
Ng31_3uM
Ng31_10uM
Ng29_1uM
Ng18_1uM
Ng18_3uM
Ng18_10uM
Ng16_1uM
Ng17_1uM
Ng22_1uM
Ng23_1uM
Ng32_1uM
Ng33_1uM
Ng34_1uM
Ng34_3uM
Ng35_1uM
Ng19_1uM
Ng19_3uM
Ng19_10uM
Ng20_1uM
Ng20_3uM
Ng20_10uM
Ng21_1uM
Ng21_3uM
Ng21_10uM
Ng1_1uM
Ng1_3uM
Ng8_1uM
Ng8_3uM
Ng8_10uM
Ng9_1uM
Ng10_3uM
Ng13_1uM
Ng14_1uM
Ng15_1uM
Ng24_1uM
Ng24_3uM
Ng25_1uM
Ng25_3uM
Ng26_1uM
Ng27_1uM
Ng27_3uM
Ng28_1uM
Blanks
GSL glycans
M3
M4
M5
M6
Mix of M4, M6, M7
M8, M9
Non mannosidic N-glycans

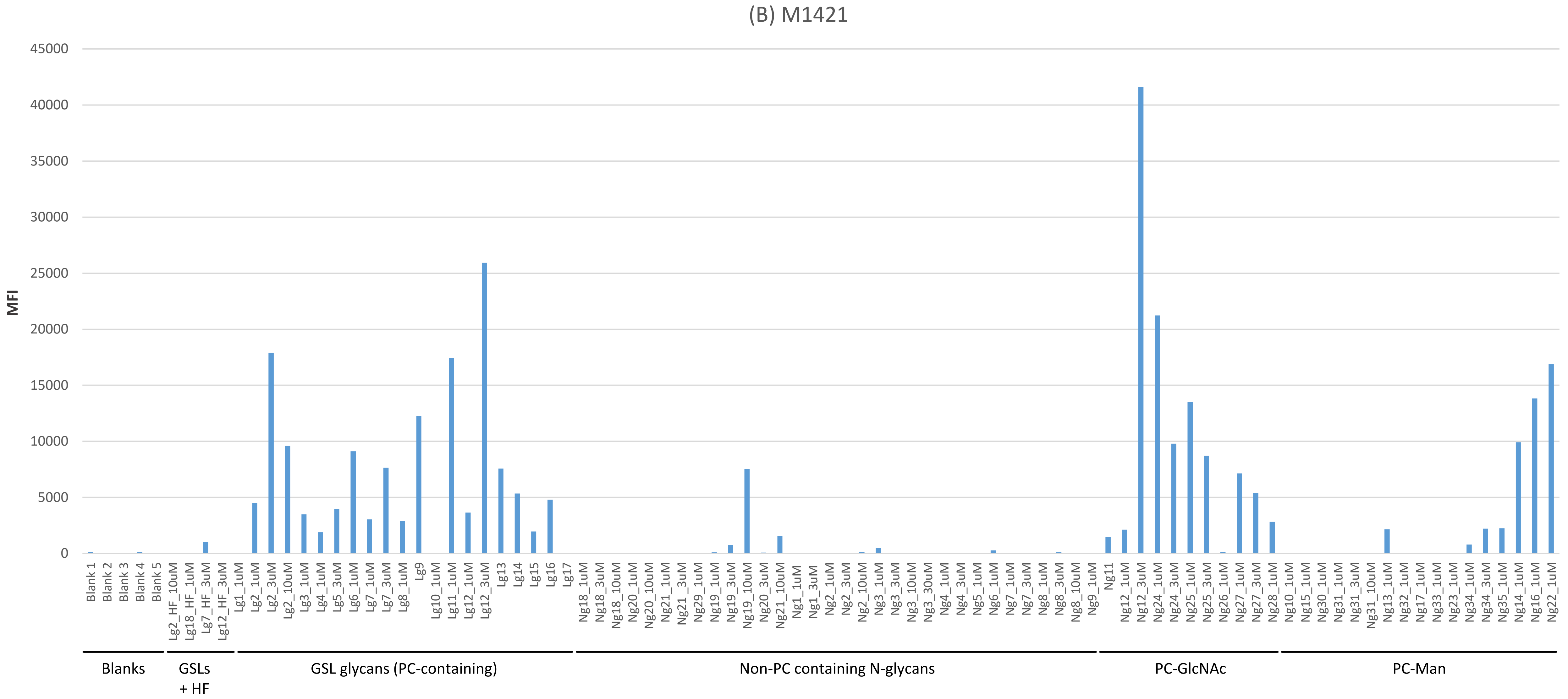
(B) M1421
MFI
45000
40000
35000
30000
25000
20000
15000
10000
5000
0
Blanks
GSLs + HF
GSL glycans (PC-containing)
Non-PC containing N-glycans
PC-GlcNAc
PC-Man

Figure S5 - Glycan array screening with longitudinal rhesus macaque infection sera

IgG and IgM binding to the *B. malayi* glycan array was measured for all animals from a cohort of 4 rhesus macaques at 4 different time-points namely pre-infection (PI), 5 weeks post-infection (wpi), 12 wpi and 15 wpi. **In (A)**, background corrected median fluorescence intensities (MFIs) are shown and each single dot corresponds to the Ig binding to a particular GSL or N-glycan containing fraction at a specific concentration for one animal: n_{dots} = 64 for N-glycans and n_{dots} = 25 for GSL glycans. Each color (orange, blue, green, purple) represents the results obtained for a different animal.

Binding to each fraction printed on the glycan microarray can be observed on the x-axis of the graphs **in (B) and (C)** (see **Table S5** for composition of each individual fractions) for the average of the 4 animals and for each individual animal, as indicated by the graph titles. The type of glycan content in the fractions is indicated below the x-axis: Blanks = negative controls (no glycans), GSL glycans or N-glycans. The 4 time-points are distinguished using the following color code: blue = pre-infection, green = 5 wpi, orange = 12 wpi and red = 15 wpi. Significant differences between time-points were assessed using Bayesian statistics. P-values < 0.05 that indicate a significant difference in fraction MFI values between pre-infection and the other time-points are represented using stars following the color code detailed in the insert on the graph upper right.

Graph raw numerical data can be found in **Table S7**.

Figure S5.A

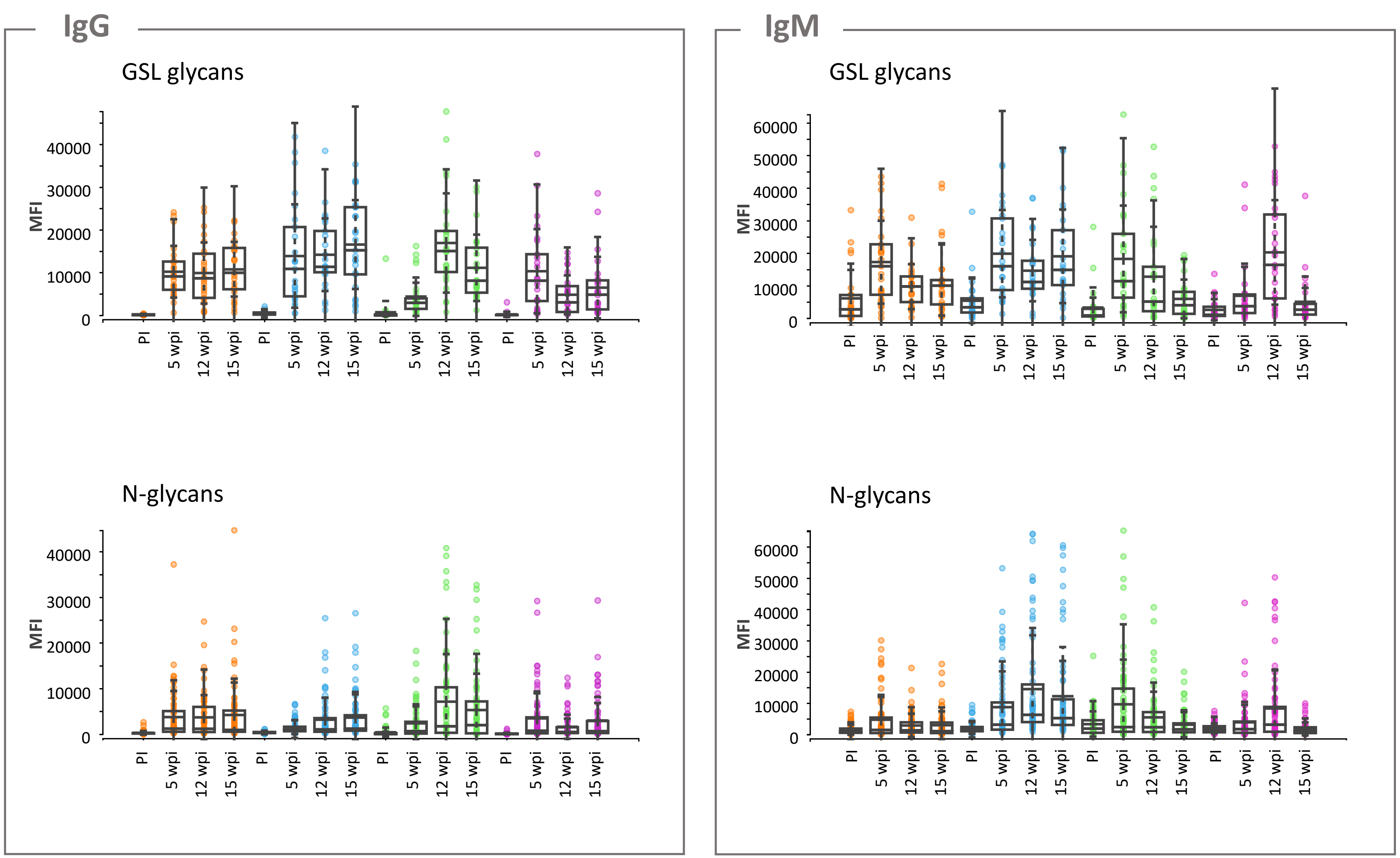
IgG
GSL glycans
N-glycans
IgM
GSL glycans
N-glycans
MFI
0
10000
20000
30000
40000
50000
60000
PI
5 wpi
12 wpi
15 wpi

Figure S5.B - IgG

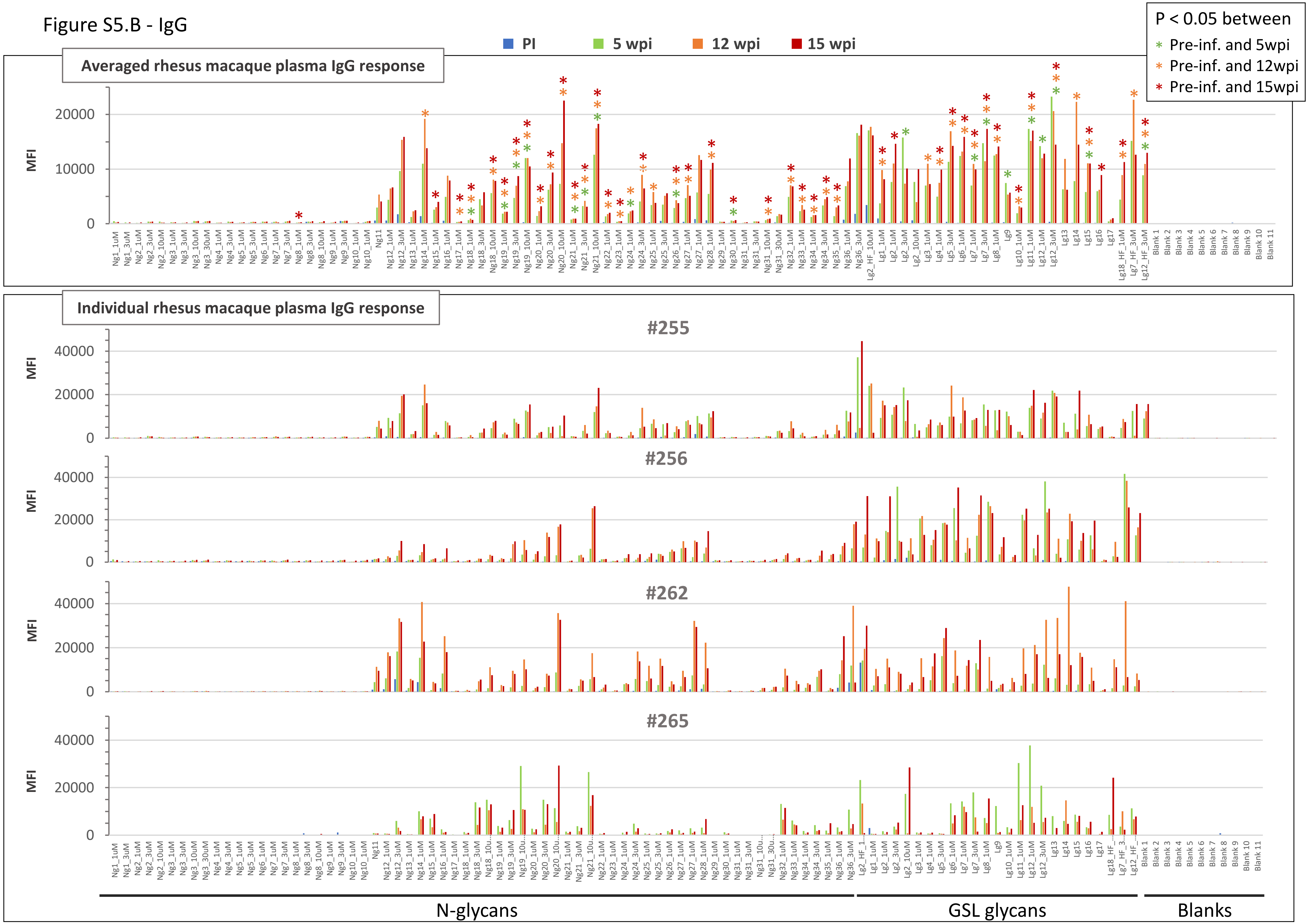

Figure S5.C - IgM

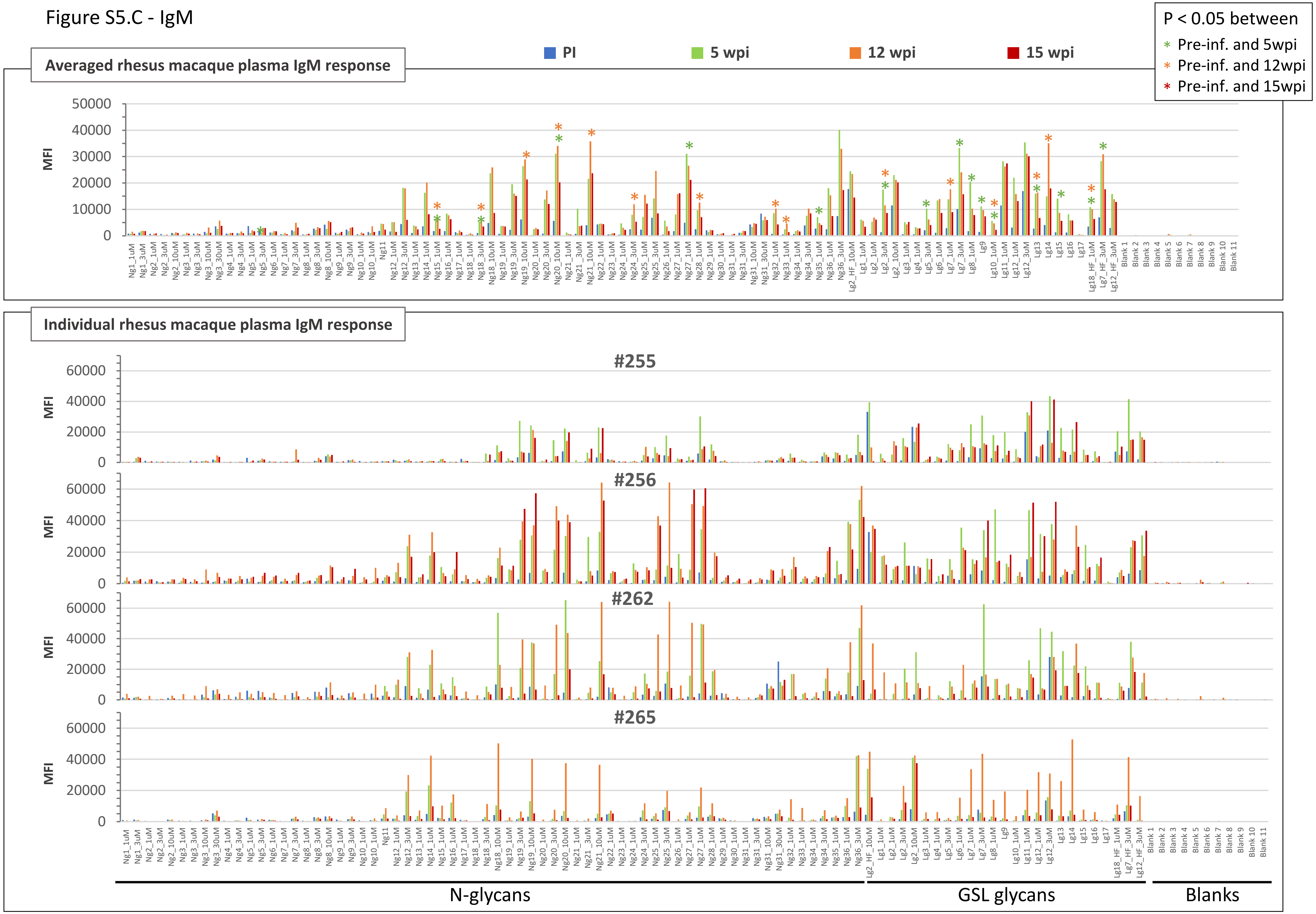

Figure S6 – Glycan array screening with healthy and infected individual plasma

Plasma IgG binding to the *B. malayi* glycan array from uninfected (n=5) and two sets of *B. malayi* infected individuals (n=5 each) was measured. Two different time points, namely pre-treatment and after DEC chemotherapy, were studied for the second set of *B. malayi* infected individuals.

In (A), boxplots of background corrected median fluorescence intensities (MFIs) are shown and each single dot corresponds to the Ig binding to a particular GSL or N-glycan containing fraction at a specific concentration for one individual: n_{dots} = 64 for N-glycans and n_{dots} = 25 for GSL glycans. Boxplots on the right plots represent the MFIs for *B. malayi* infected individuals (orange) and uninfected individuals (blue). Boxplots on the left plots represent the MFIs for *B. malayi* infected individuals pre (orange) and post (blue) DEC treatment.

Details of binding to the various fractions for each individual is provided in graphs **B to D**. Printed fractions are plotted on the x-axis of the graphs (see **Table S5** for fraction compositions) and the type of glycan content in the fractions is indicated below the x-axis: Blanks = negative controls (no glycans), GSL glycans or N-glycans.

Raw numerical data can be found in **Table S8**.

Figure S6.A

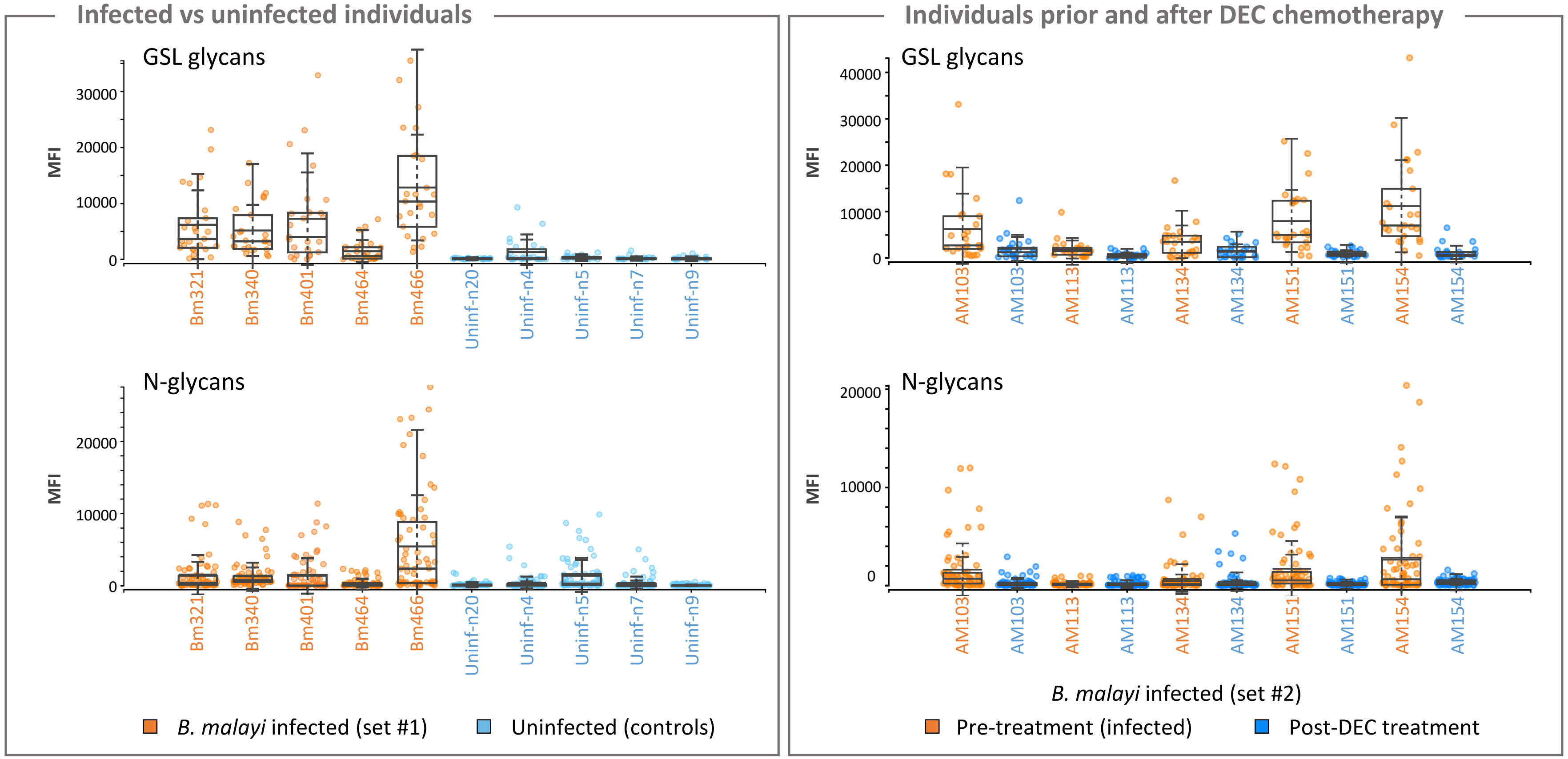
Infected vs uninfected individuals
GSL glycans
MFI
30000
20000
10000
0
Bm321
Bm340
Bm401
Bm464
Bm466
Uninf-n20
Uninf-n4
Uninf-n5
Uninf-n7
Uninf-n9
N-glycans
MFI
20000
10000
0
B. malayi infected (set #1)
Uninfected (controls)
Individuals prior and after DEC chemotherapy
GSL glycans
40000
30000
20000
10000
0
AM103
AM113
AM134
AM151
AM154
N-glycans
20000
10000
0
B. malayi infected (set #2)
Pre-treatment (infected)
Post-DEC treatment

Figure S6.B – *B. malayi* infected donors (Set #1)

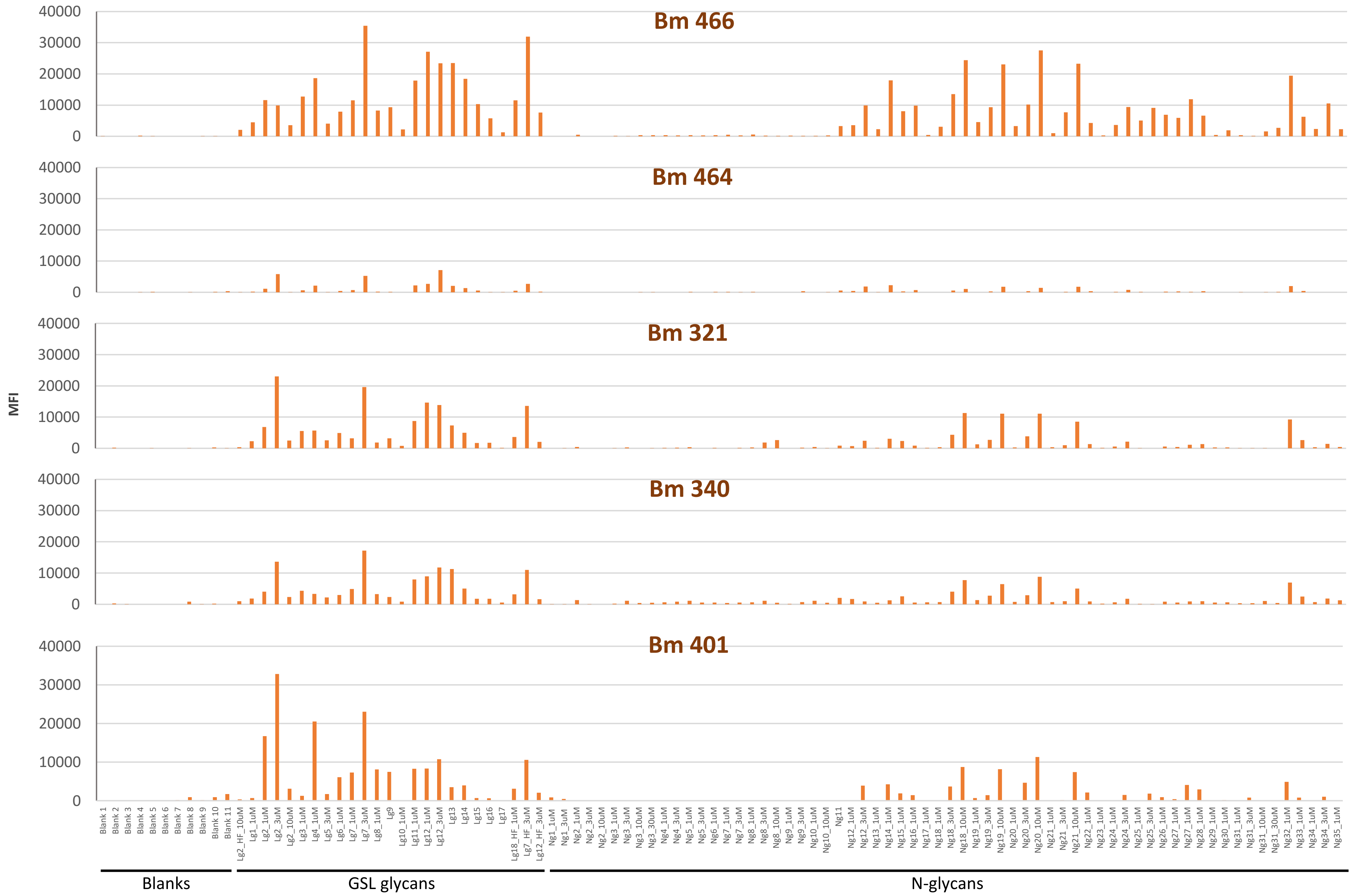

Figure S6.C – Uninfected donors (Controls)

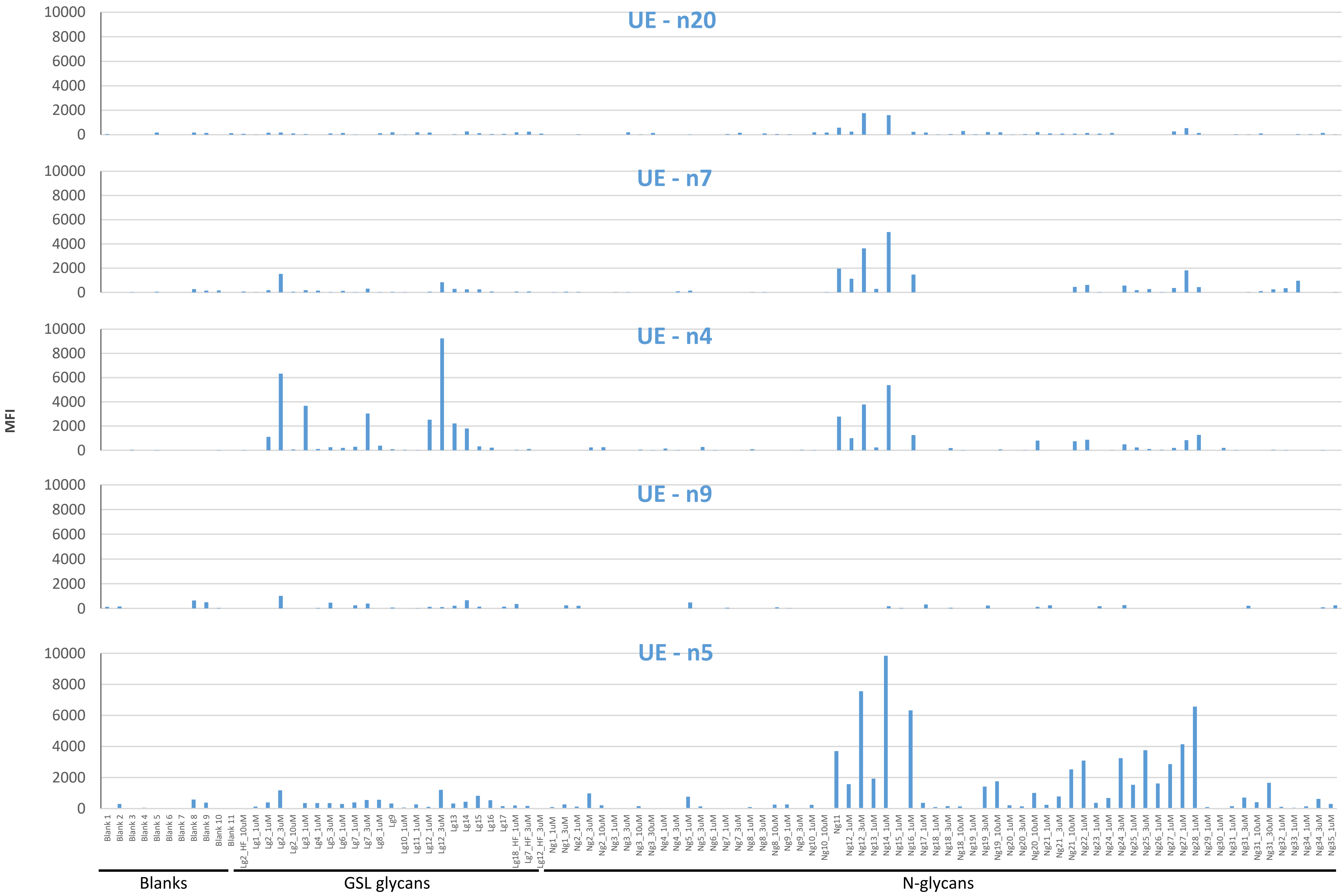

Figure S6.D - *B. malayi* infected donors (Set #2)

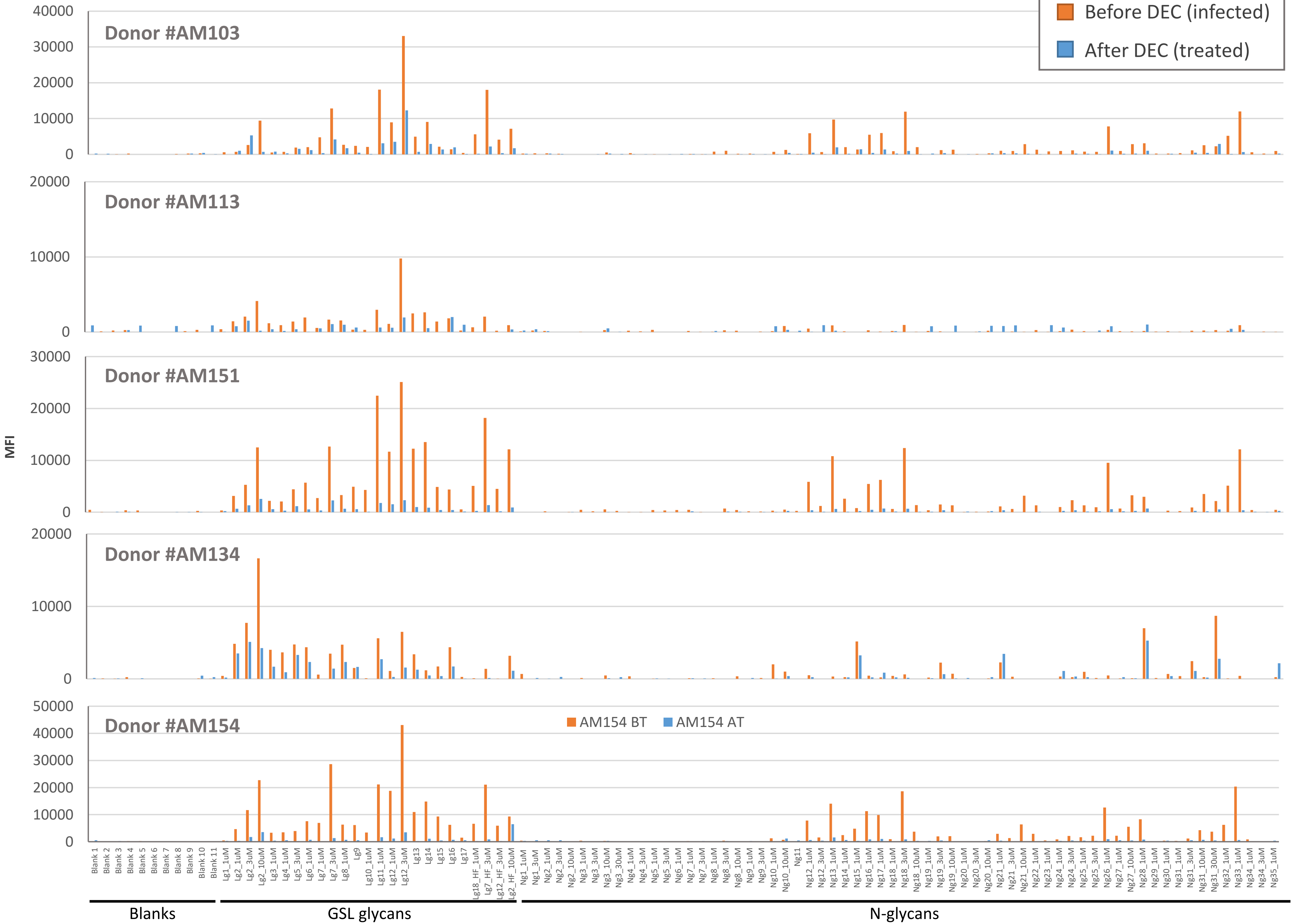

Supplement: Supplemental Figures S1–S6 [file mmc9.pdf]
